# Single-cell machine learning uncovers genetically anchored, cell-type specific programs of Alzheimer’s disease

**DOI:** 10.1038/s41467-026-77651-5

**Published:** 2026-11-09

**Authors:** Adithya V. Madduri, Randall J. Ellis, Chirag M. Lakhani, David A. Bennett, Chirag J. Patel

**Affiliations:** 1 Department of Biomedical Informatics, Harvard Medical School, Boston, MA USA; 2 Harvard College, Cambridge, MA USA; 3 New York Genome Center, New York, NY USA; 4 Rush Alzheimer’s Disease Center, Rush University, Chicago, IL USA

**Keywords:** Alzheimer's disease, Gene expression, Machine learning

## Abstract

Univariate differential expression analysis dominates single-cell transcriptomic studies of Alzheimer’s disease, yet marginal testing alone cannot resolve multivariate disease-associated structure or distinguish clinical from neuropathological signatures. Here we introduce TriSCOPE, a framework integrating multivariate predictive modeling, donor-aware differential expression, and pseudotime inference, applied to six Alzheimer’s disease snRNA-seq datasets comprising ~4.2 million nuclei from 627 donors. Across six major brain cell types and 41 subclusters, we identify cell-type-specific predictively expressed genes that are largely non-overlapping with differentially expressed genes, revealing disease-relevant signals invisible to univariate approaches. Conditional GWAS–eQTL colocalization anchors a subset of these predictively expressed genes to inherited Alzheimer’s disease risk loci, including 27 genetically supported gene–cell type pairs (among them *ARL17B*, *JAZF1*) with no significant differential expression. Integration across all three analytical branches nominates high-confidence convergent programs including astrocytic stress markers (such as *GFAP*, *MT2A*), neuronal signaling genes (like *RASGEF1B*, *LINGO1*, *NRG1*), and microglial and oligodendrocyte regulators (including *SYTL3*, *SPP1*, *APOD*). Comparison of clinical and neuropathological models reveals cell-type-specific divergence between cognitive and pathological Alzheimer’s disease signatures. Key predictively expressed genes including *ARL17B*, *LINGO1*, and *APOE* replicate across independent cohorts, with cross-cohort generalizability confirmed across all three analytical branches of TriSCOPE.

## Abstract (web summary)

Univariate analyses alone cannot resolve multivariate disease-associated structure. Here, the authors develop TriSCOPE, a machine learning approach that identifies gene programs specific to individual brain cell types in Alzheimer’s disease.

## Introduction

Alzheimer’s disease (AD) is a progressive neurodegenerative disorder marked by the accumulation of amyloid-β plaques, intracellular neurofibrillary tangles (NFTs) in neurons, and cognitive decline, and is inextricably linked with age^1–4^. Despite decades of research, AD continues to affect millions worldwide and remains a major public health challenge^5,6^. Single-nucleus RNA sequencing (snRNA-seq) has revealed cell-type-specific transcriptional alterations in AD^7,8^, but most studies rely on univariate differential expression (DE) analyses, which identify hundreds to thousands of marginally associated genes per cell type without resolving redundancy among correlated transcripts or distinguishing which genes remain informative when modeled jointly. As a result, DE alone cannot capture the multivariate structure of disease-associated transcriptional programs or identify disease-relevant genes whose signal emerges only when modeled jointly with covariates.

Multivariate machine learning offers a complementary approach: by introducing implicit regularization and competition among correlated features, supervised models prioritize genes that contribute independent predictive information beyond other transcripts and known covariates such as age, sex, and APOE genotype^9–11^. Rather than serving as a prognostic tool, machine learning in this context functions as a hypothesis-sharpening instrument, compressing thousands of candidate genes into a focused, multivariately informative subset that can then be interrogated through orthogonal genetic and trajectory-based analyses.

Here we introduce TriSCOPE, an integrative machine learning framework that combines: (i) multivariate predictive modeling using AutoML, (ii) differential expression analysis, and (iii) pseudotime-based trajectory inference. By incorporating orthogonal evidence beyond univariate fold change, TriSCOPE enables convergent validation of disease-relevant genes and reduces false positives^12^. Here we applied TriSCOPE across six snRNA-seq datasets comprising ~4.2 million nuclei from 627 donors (Table 1)^7,13–15^, all from the Dorsolateral prefrontal cortex (PFC), a region centrally involved in the executive dysfunction characteristic of clinically manifest AD^16^.

**Table 1:** Data summary

COHORT	SHORT NAME	CITATION	N	NUCLEI	REGION	PREDICTIVE MODELING	DIFFERENTIAL EXPRESSION	PSEUDOTIME
ROSMAP 2019 Release	Mathys-48	Mathys et al.	48	70,634	DLPFC	✓	✓	✓
Lau et al. SWDBB	Lau-21	Lau et al.	21	172,514	PFC	✓	—	—
ROSMAP 2023 Release	ROSMAP-427	Mathys et al. 2023	427	~2,300,000	DLPFC	✓	✓	—
Murphy/Skene (Reprocessed Mathys 2019)	Murphy-48	Murphy et al.	48*	50,381*	DLPFC	✓ QC validation	—	—
ROSMAP 2024 Release	ROSMAP-48b	Mathys et al. 2024	48	254,721	DLPFC	—	✓	—
SEA-AD	SEA-AD	Gabitto et al.	83	1,395,601	PFC	✓	✓	✓

^*^Not counted in totals (reprocessed Mathys 2019 data). Total unique: 627 donors · ~4,193,470 nuclei.

Summary of single-nucleus RNA-seq datasets analyzed in this study, indicating which TriSCOPE analytical branches were applied to each cohort.

Across six major brain cell types and 41 subclusters, we show that predictively expressed genes (PEGs) are largely non-overlapping with DEGs, and that conditional GWAS–eQTL colocalization anchors a subset of these PEGs to inherited AD risk loci, including 27 genetically supported genes whose disease relevance is entirely missed by differential expression. Integration across all three branches of TriSCOPE nominates convergent, cell-type-resolved programs, including candidates such as HS3ST4 and SYTL3 in microglia, NRG1 in neurons, and metallothioneins MT2A and MT3 in astrocytes. We then demonstrate that clinical and neuropathological models reveal divergent molecular signatures across cell types^17–19^, and that these findings replicate in independent cohorts. Together, our results establish that jointly modeling genetic, demographic, and transcriptomic axes uncovers genetically anchored molecular programs of AD that are invisible to univariate transcriptomic analyses alone.

## Results

### Predicting AD using demographics, APOE genotype, and gene expression

**Fig. 1:**
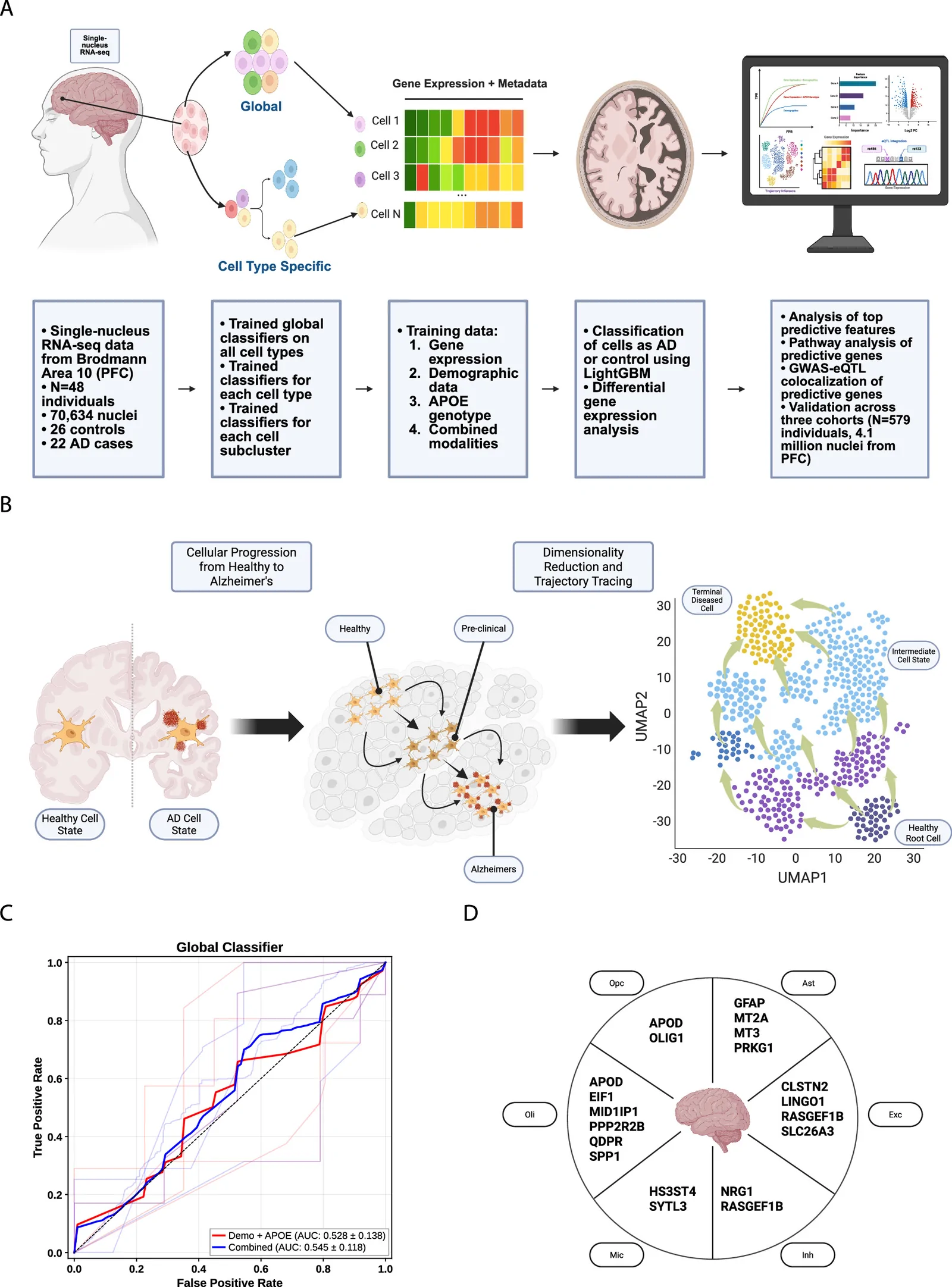
Overview of the TriSCOPE framework for integrative single-cell predictive and trajectory analysis in Alzheimer’s disease. **A** Outline of the predictive modeling component of our TriSCOPE approach. Including cell-specific disease classification and downstream feature importance analysis. Created in BioRender. M, A. https://BioRender.com/xvqsmfa. **B** Outline of the pseudotime modeling component of TriSCOPE. We choose cells based on healthy phenotype: no Amyloid pathology (CERAD), and no cognitive impairment (COGDX). Setting the transcriptional state of this healthy cell as our root, we then trace the transcriptional progression away from this cell in order to map the progression of disease. We perform this at the cell subcluster level to avoid confounding with transcriptional differences between cell types. Created in BioRender. M, A. https://BioRender.com/4tdsbmf. **C** A predictive classifier trained across five splits on a global scale, no cell type differentiation, on Demographics and APOE data. The second curve shows a predictive classifier trained across five splits on a global scale, no cell type differentiation, on Demographics, APOE, and Gene Expression data. **D** Circle plot summarizing a high-confidence set of gene features jointly supported across the three branches of the TriSCOPE framework. Genes shown were selected based on concordant evidence from predictive modeling, donor-aware differential expression, and pseudotime analysis: specifically, genes that were (i) predictive in at least two independent model splits, (ii) differentially expressed with FDR-adjusted *P* < 0.05 and |log₂ fold change | > 0.25, and (iii) ranked within the top 1% of trajectory-associated genes based on pseudotime influence. Genes are displayed by major brain cell type, including Microglia (Mic), Astrocytes (Ast), Excitatory Neurons (Exc), Inhibitory Neurons (Inh), Oligodendrocytes (Oli), and Oligodendrocyte Progenitor Cells (Opc), highlighting convergent molecular candidates implicated in Alzheimer’s disease vulnerability. No statistical test was applied to the intersection itself; genes were selected by jointly applying the three criteria above. Differential expression significance was determined using a donor-aware Poisson generalized linear mixed model (two-sided Wald test, Benjamini–Hochberg corrected). Created in BioRender. M, A. (https://BioRender.com/gsdej2o). Source data are provided as a Source Data file.

We first applied the predictive modeling branch of TriSCOPE to identify molecular signatures of clinical Alzheimer’s disease (AD) that extend beyond known risk factors, developing an interpretable machine learning framework that prioritizes gene expression features predictive of clinical AD. Clinical AD was defined by the diagnosis rendered at the last visit while alive and was adjudicated by a panel of neurologists, neuropsychologists, and other clinicians. Our goal was to use machine learning to uncover combinations of demographic, genetic, and transcriptomic features that confer cellular vulnerability on a phenotype specific basis. We applied this approach to 70,634 single-nucleus transcriptomes from human postmortem PFC (Mathys-48^7^), with validation in two independent cohorts across 1.5 million nuclei (SEA-AD^15^, Lau-21^13^) (Fig. 1A and Table 1).

We first trained a baseline model using only demographic features (age at death, sex, and years of education) and APOE genotype to predict clinical AD diagnosis. This model performed only slightly above chance (mean AUC = 0.528 ± 0.138), highlighting the limited explanatory power of demographics and APOE genotype at single-cell resolution (Fig. 1C). Incorporating gene expression features alongside demographics and APOE genotype modestly improved classification performance (mean AUC = 0.545 ± 0.118) (Supplementary Data 4), suggesting that transcriptional profiles contain additional predictive information not captured by demographics and APOE genotype (Fig. 1C). This modest gain in predictive power motivated examining gene expression within individual cell types to identify genes that drive disease classification and the predictive performance of these models beyond known risk factors.

### Cell type-specific biomarker discovery beyond demographics and APOE genotype

**Fig. 2:**
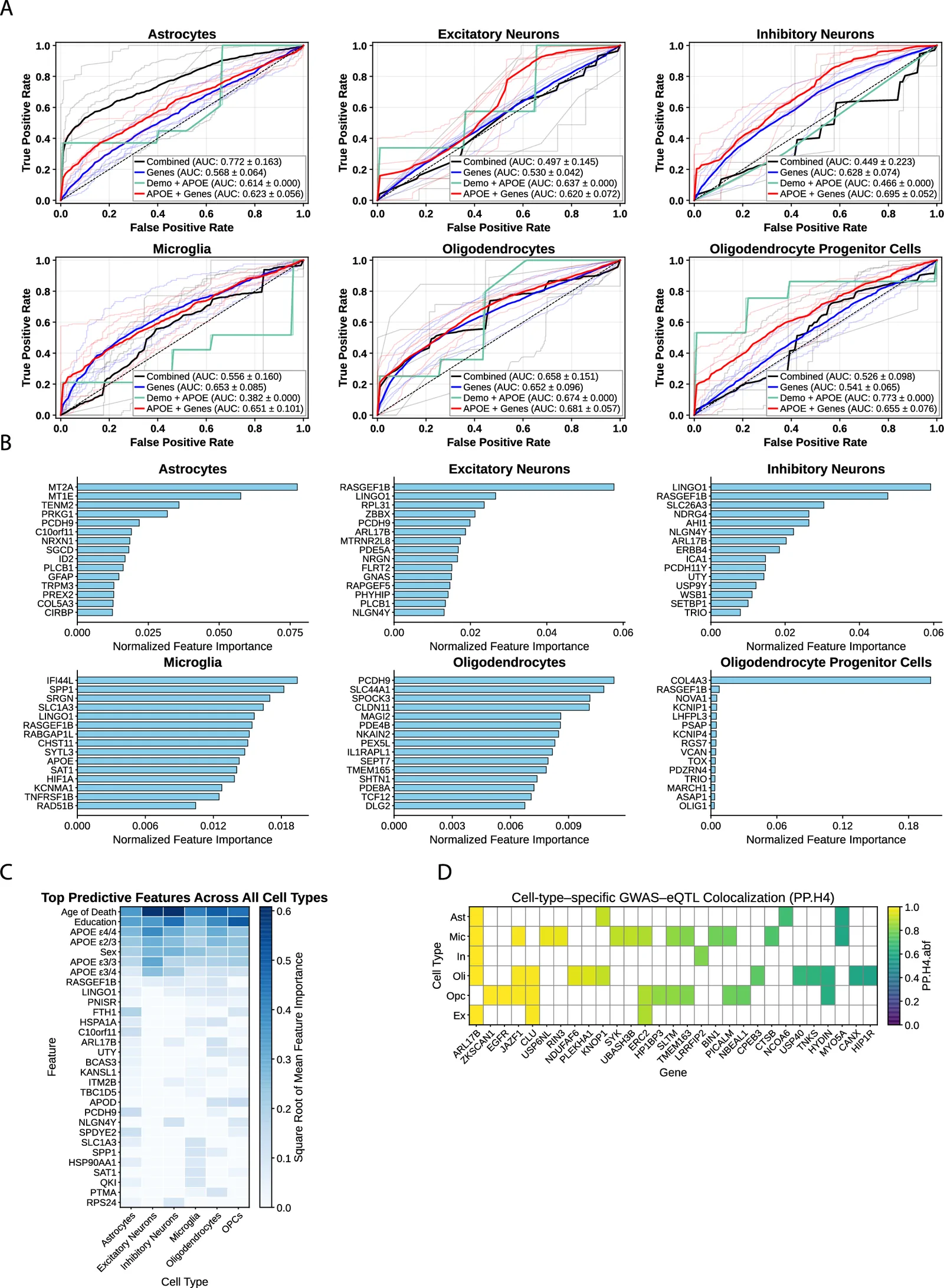
Cell-type-specific predictive modeling reveals diverse transcriptional determinants of clinical Alzheimer’s disease status. **A** Receiver operating characteristic (ROC) curves summarizing the results from five independent runs of classification experiments performed on six major brain cell types: Astrocytes, Excitatory Neurons, Inhibitory Neurons, Microglia, Oligodendrocytes, and Oligodendrocyte Progenitor Cells. For each cell type, classifiers were trained using four feature sets: (1) a combined set including demographic variables (age at death, sex, education), APOE genotype, and gene expression data (black curves); (2) demographic variables and APOE genotype only (gray curves); (3) gene expression plus APOE genotype (red curves); and (4) gene expression only (blue curves). Mean area under the curve (AUC) values ± standard deviation are reported in the figure legends for each curve. Lighter lines represent individual splits; bold lines indicate mean performance. Higher AUC values reflect better discrimination between Alzheimer’s disease and control cells. **B** Top predictive genes identified from gene expression–based classifiers across major brain cell types. Normalized feature importance scores for the top 15 gene features used to classify Alzheimer’s disease versus control cells, shown separately for each major brain cell type: Astrocytes, Excitatory Neurons, Inhibitory Neurons, Microglia, Oligodendrocytes, and Oligodendrocyte Progenitor Cells. Feature importance was computed for each gene as the raw importance assigned by the classifier in each of five independent train-test splits, divided by the sum of all raw importances in that split to obtain a normalized score. Mean normalized importance across five splits was then calculated for each gene, and the 15 genes with the highest mean scores per cell type are displayed. Standard deviations are reported in Supplementary Data 2. Bars represent mean normalized importance, highlighting genes most strongly contributing to Alzheimer’s disease prediction in each cell type. **C** Heatmap showing the top 30 most predictive features from the combined classifiers trained on demographic variables (age at death, sex, education), APOE genotype, and gene expression data, separately for each major cell type: Astrocytes, Excitatory Neurons, Inhibitory Neurons, Microglia, Oligodendrocytes, and Oligodendrocyte Progenitor Cells OPCs. Feature importance for each feature was computed as the raw importance assigned by the classifier in each of five independent splits, normalized by the total importance within each split. The square root of the mean normalized importance across splits is shown for visualization. Rows represent features (genes and demographic variables), and columns represent cell types. Color intensity indicates relative feature importance, highlighting which features most strongly contributed to Alzheimer’s disease versus control classification in each cell type. **D** Heatmap summarizing GWAS–eQTL colocalization results for TriSCOPE-prioritized predictive genes, with shading indicating the posterior probability of a shared causal variant (PP.H4) under a Bayesian colocalization model. Source data are provided as a Source Data file.

To investigate how transcriptional vulnerability to Alzheimer’s disease varies across brain cell types, we trained separate models for each major population: Astrocytes, Excitatory Neurons, Inhibitory Neurons, Microglia, Oligodendrocytes, and Oligodendrocyte Progenitor Cells (OPCs). Specifically, within each cell type, we compared the performance of models with the following combinations of predictor variables 1) Demographics and APOE genotype, 2) Gene expression alone, 3) APOE genotype and Gene expression alone, and 4) the Combination of demographics, APOE genotype, and gene expression (Fig. 2A).

Our AD classifiers all demonstrated consistent performance across cell types (mean AUC: 0.620 ± 0.072 to 0.695 ± 0.052) using gene expression and APOE genotype alone relative to demographics and APOE genotype (Supplementary Data 4 and Fig. 2A). To assess the robustness of these findings to model choice, we benchmarked four commonly used tabular classifiers—gradient boosted decision trees (GBM)^20^, LASSO-regularized logistic regression^21^, elastic net^22^, and random forest^23^ across all six cell types (Supplementary Data 16). Across cell types, all tested architectures achieved relatively concordant performance above chance, while donor-level label permutation controls consistently yielded AUCs below 0.50, confirming that the predictive signal was not driven by data leakage or overfitting. GBM models exceeded permuted controls by a mean ΔAUC of 0.114 ± 0.056 across cell types and achieved the highest performance of the four benchmarked models in two of six populations (Supplementary Data 16). We focus on GBM models in all downstream analyses, as they combine competitive predictive performance with the ability to model nonlinear feature interactions and yield interpretable feature importance estimates suitable for biological interpretation.

To identify the molecular signatures underlying each model’s performance, we next ranked feature importance scores across all cell types, both in our combined data modality models and gene expression-only models. In the combined-modality model, age at death, education, APOE genotype, sex, and a small set of genes including ARL17B, HSPA1A, RASGEF1B, and LINGO1 consistently ranked among the most predictive features across cell types (Fig. 2C), indicating the presence of shared transcriptional signals associated with Alzheimer’s disease beyond known risk factors.

While some PEGs were shared across cell types, others were highly specific (Supplementary Data 2). For example, excitatory neuron models prioritized mitochondrial and synaptic genes^24^, inhibitory neuron models highlighted ion transport and sex-linked transcripts^25^, microglial models emphasized immune and stress-response genes (e.g., *IFI44L*, *SRGN*, *HIF1A*)^26–28^, and oligodendrocyte models preferentially selected genes involved in myelin integrity and proteostasis (e.g., *CRYAB*, *CLDN11*, *APOD*)^29,30^. Astrocyte models similarly revealed *ID2* and *FTH1*^31^ while OPC models revealed *COL4A3*, underscoring the heterogeneity of transcriptional programs associated with disease across cell types (Fig. 2B and Supplementary Data 2)^32,33^. When predictive modeling was scaled to the full ROSMAP-427 cohort (427 donors), significant PEG overlap with the Mathys-48 discovery set was observed across all six cell types (fold enrichment 2.77–32.89, all FDR-corrected *p* < 0.006), including key genes such as *APOE*, *IFI44L*, and *HS3ST4* in Microglia and *ARL17B*, *CRYAB*, and *LINGO1* in Oligodendrocytes (Supplementary Results).

The recovery of established Alzheimer’s disease–related genes among these cell-type–specific PEGs supports the biological validity of our modeling approach and suggests that PEGs with limited prior characterization may also represent meaningful disease-associated programs (Fig. 2B). Together, these results demonstrate that multivariate, cell-type–resolved modeling identifies a focused set of pan-cell-type and cell-type–specific biomarkers that are not apparent from demographic risk factors alone.

### Donor-aware differential expression contextualizes cell-type-specific predictors of AD

**Fig. 3:**
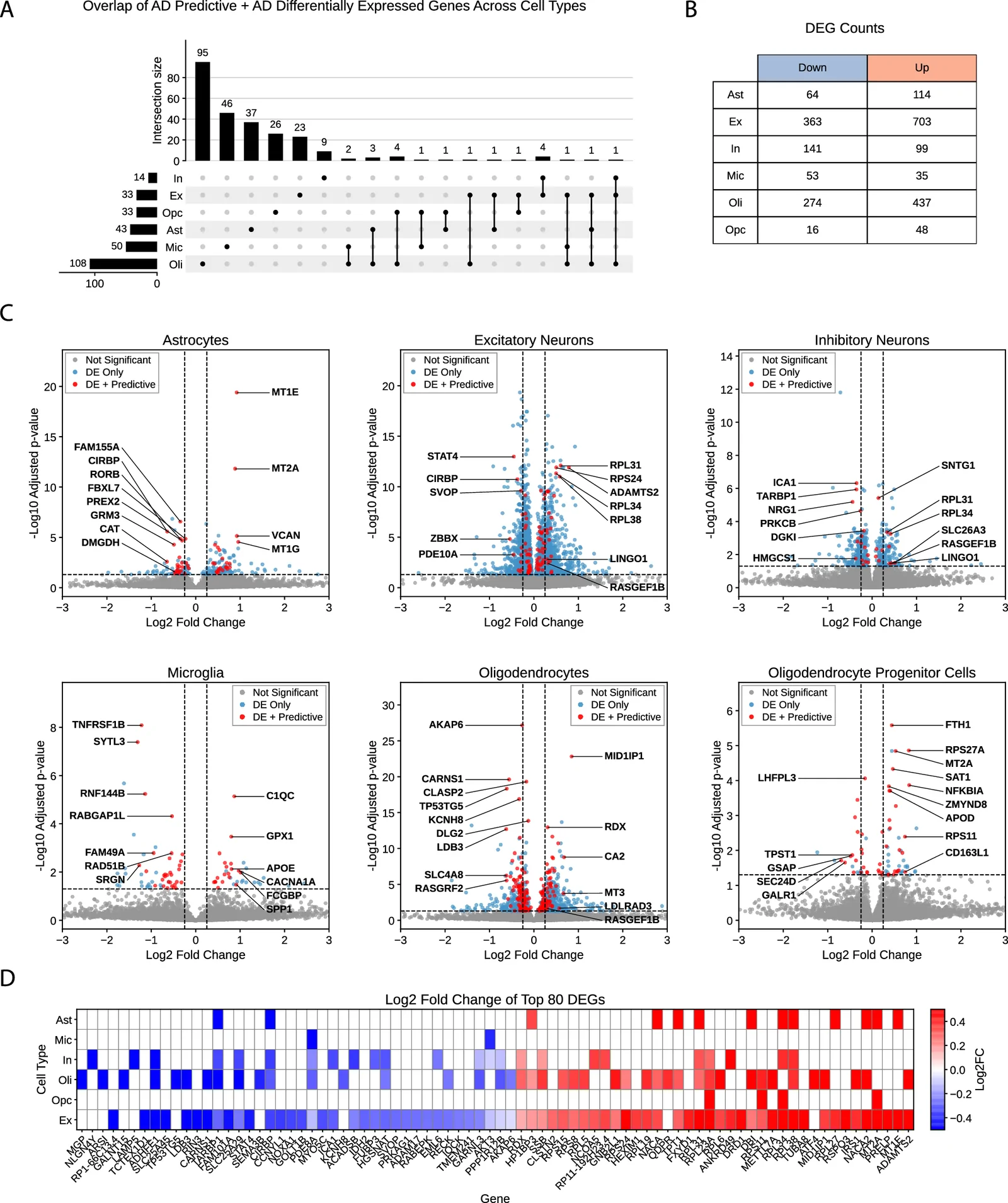
Integration of predictive and differential expression signatures identifies convergent disease markers across cell types. **A** Upset plot illustrating the overlap between genes identified as predictive (assigned nonzero feature importance in at least two out of five independent classifier splits) and genes identified as differentially expressed (FDR-adjusted *P* < 0.05 and |log₂ fold change | > 0.25) in Alzheimer’s disease versus control cells, analyzed separately for each major brain cell type: Astrocytes Ast, Excitatory Neurons Exc, Inhibitory Neurons Inh, Microglia Mic, Oligodendrocytes Oli, and Oligodendrocyte Progenitor Cells Opc. Bar heights represent intersection sizes, and connected dots indicate specific combinations of cell types sharing overlapping genes. Notably, the strongest overlap was observed in astrocytes and excitatory neurons, suggesting a substantial convergence of predictive and transcriptionally dysregulated signals in these cell types. **B** Table summarizing the number of differentially expressed genes (DEGs) identified as significantly downregulated (blue column) or upregulated (orange column) in Alzheimer’s disease versus control cells across each major brain cell type. Differential expression was performed using a donor-aware Poisson generalized linear mixed model with a log link, an offset for library size, RUV covariates as fixed effects, and a random intercept for donor (FDR-adjusted *P* < 0.05 and |log₂ fold change | > 0.25). **C** Volcano plots showing differentially expressed genes (DEGs) in Alzheimer’s disease versus control cells for each major brain cell type: Astrocytes, Excitatory Neurons, Inhibitory Neurons, Microglia, Oligodendrocytes, and Oligodendrocyte Progenitor Cells. Genes are plotted by log2 fold change (*x*-axis) and –log10 FDR-adjusted *P* value (*y*-axis). Genes significant for differential expression (FDR-adjusted *P* < 0.05) and also identified as predictive (nonzero feature importance in at least two splits) are highlighted in red. Genes significant for differential expression only are shown in blue, and non-significant genes are shown in gray. Selected predictive DEGs are labeled, illustrating key markers that contribute to both differential transcriptional changes and disease-state classification within each cell type. **D** Heatmap displaying log2 fold changes of the top 80 differentially expressed genes (DEGs) in Alzheimer’s disease versus control cells across six major brain cell types. Genes were selected based on having an absolute log2 fold change >0.2 in at least one cell type and were ranked by minimal FDR-adjusted *P* value across all cell types. Color intensity reflects log2 fold change, with red indicating upregulation and blue indicating downregulation in Alzheimer’s disease. Only genes with FDR-adjusted *P* < 0.05 are shown. *P* values in **B**–**D** are from two-sided Wald tests and were adjusted for multiple comparisons using the Benjamini–Hochberg procedure; panel **A** is descriptive and involves no statistical test. Source data are provided as a Source Data file.

To contextualize and further interpret PEGs identified by predictive modeling, we used the second branch of TriSCOPE to perform differential expression analysis between AD and control samples within each major cell type, accounting for the donor of origin using a random effect. In Excitatory Neurons, many of our top PEGs such as *LINGO1*, *RASGEF1B*, and *RPL31* were significantly upregulated in AD while another predictor *ZBBX* was downregulated (Supplementary Data 8). While differential expression analysis revealed over 1066 genes (FDR-corrected *p* value < 0.05, abs(log2FC) > 0.25; Fig. 3B) altered in clinical AD within Excitatory Neurons, a much smaller subset of only 162 features were PEGs (33 overlapping, Fig. 3A), highlighting a much more focused core of disease-relevant changes when using a multivariate machine learning approach as opposed to a univariate differential expression approach. Similarly in Microglia, several top PEGs were also significantly differentially expressed including *SPP1*, the second highest-ranking predictor, which was significantly upregulated (log₂FC = 0.92, FDR = 0.033) (Fig. 3C). *SRGN* (log₂FC = −1.26, FDR = 0.005) and TNFRSF1B (log₂FC = −1.21, FDR = 8e-9) associated with immune response were downregulated while APOE (log₂FC = 0.81, FDR = 0.007) was upregulated (Fig. 3C and Supplementary Data 8). Oligodendrocytes had the largest PEG-DEG overlap with 108 genes.

Overall, we found varying overlap between our PEGs and DEGs (3.85–24.57% of predictive genes were DEGs across cell types). When evaluated against an independent ROSMAP cohort of 427 donors, only 2–8% of PEGs were differentially expressed, and Spearman correlations between predictor rank and absolute log₂ fold change were modest (ρ ≈ 0.02–0.21), with similar patterns observed across four independent single-cell AD studies (Supplementary Results; Supplementary Data 18). These results indicate that TriSCOPE nominated PEGs capture multivariate disease-associated structure not reducible to transcriptional effect size alone.

### Genetic colocalization anchors cell-type–specific transcriptional predictors

We next asked whether our PEGs were supported by genome-wide significant AD loci. Using the NHGRI-EBI GWAS Catalog^34^, we tested whether PEGs were proximal to previously reported AD-associated SNPs, and subsequently assessed whether these SNPs act as cis-eQTLs in the brain.

PEGs in Inhibitory Neurons, Microglia, Oligodendrocyte Progenitor Cells, and Oligodendrocytes were significantly enriched for genes containing genome-wide significant SNPs (Fisher’s *p* range=2.36e-5−0.047). In Oligodendrocytes, OPCs, and Excitatory Neurons, the strongest GWAS signal overlapping PEGs mapped to the CLU locus (-log10p = 43.7) (Supplementary Fig. 5a and Supplementary Data 5). Microglia showed 68 PEGs overlapping GWAS genes (OR = 1.31, Fisher’s *p* = 0.047), including *APOE*, *BIN1*, *PICALM*, and *RASGEF1C* (Supplementary Fig. 5a and Supplementary Data 5). The strongest GWAS signal overlapping Astrocyte PEGs mapped to the ABCA1 locus (-log10p = 32).

To more formally link genetic risk to gene regulation, we performed GWAS-eQTL colocalization analyses using brain tissue expression quantitative trait loci^35^. Importantly, colocalization analyses were restricted to PEGs, such that the reported proportions reflect the fraction of PEGs with genetic support rather than genome-wide colocalization rates. We quantified PP.H4, the posterior probability under a Bayesian colocalization model that a shared causal variant underlies both the GWAS and eQTL association signals at a locus. For each cell type, we identified multiple loci where AD GWAS signals colocalized with cis-eQTLs regulating expression of PEGs, indicating a shared genetic architecture underlying disease risk and transcriptional dysregulation (Fig. 2D). Across 1,107 independent genes, we identified 144 colocalization tests with moderate or stronger evidence of colocalization (PP.H4 ≥ 0.5) and 105 with strong evidence of a shared causal variant (PP.H4 ≥ 0.8) (Supplementary Data 17). Strong colocalization (PP.H4 ≥ 0.8) was observed for ~1–2% of PEGs across cell types (Supplementary Data 17), which we refer to as coloc-PEGs. Although the absolute fraction of predictive genes with strong colocalization was modest, this rate reflects enrichment within a pre-selected, multivariately predictive gene set rather than genome-wide colocalization frequencies, and therefore represents a stringent test of shared genetic architecture.

Among the strongest signals, ARL17B demonstrated near-complete colocalization (PP.H4 ≈ 0.99) across multiple brain regions and cell types, indicating that genetic variants influencing ARL17B expression likely underlie the associated AD GWAS signal (Fig. 2D). We additionally observed robust colocalization for CLU in excitatory neurons, oligodendrocytes, and oligodendrocyte progenitor cells. Cell-type-specific colocalization was also observed for EGFR in OPCs, USP6NL in microglia, and mitochondrial-associated genes such as NDUFAF6 in oligodendrocytes, suggesting again that distinct genetic regulatory programs contribute to disease risk across cellular compartments (Fig. 2D).

A particularly informative subset of coloc-PEGs was those that were *not* differentially expressed (FDR-adjusted *p* < 0.05, |log₂FC | > 0.25). These genes represent candidates whose disease relevance would be entirely missed by standard transcriptomic analyses: they do not exhibit significant marginal expression shifts between AD and control, but are nonetheless genetically supported by shared GWAS–eQTL architecture and contribute to multivariate disease classification. Across cell types, we identified 27 such gene–cell type pairs (Supplementary Data 17). *ARL17B* was the most prominent, appearing in five cell types (Astrocytes, Microglia, Inhibitory Neurons, Excitatory Neurons, and Oligodendrocytes). In Microglia, an additional seven genes met this criterion, including *JAZF1*, a transcription factor at a genome-wide significant AD locus implicated in lipid metabolism and insulin signaling; *SYK*, a tyrosine kinase central to microglial phagocytosis and neuroinflammatory signaling; and *RIN3*, an endocytic adaptor protein at the *BIN1*/*RIN3* AD risk locus. In OPCs, seven genes were identified, and in Oligodendrocytes, five genes, including *PLEKHA1*, linking genetic risk to phosphoinositide signaling. *ERC2*, involved in neurotransmitter release at active zones, appeared in three cell types (Microglia, OPCs, and Excitatory Neurons), representing a cross-cell-type genetically anchored predictor invisible to differential expression. These findings demonstrate that GWAS–eQTL colocalization, when applied conditionally to genes prioritized by predictive modeling, reveals a class of genetically supported disease candidates that occupy a blind spot of univariate transcriptomic analyses.

### Clinical and neuropathological predictors diverge across cell types

Given AD’s two hallmarks, cognitive decline and ATN (amyloid, tau, and neurodegenerative) pathology, we suspected that gene expression and other biological features may explain AD neuropathology (i.e., Consortium to Establish a Registry for Alzheimer’s Disease (CERAD) score) differentially from clinical AD diagnosis adjudicated by a panel of neurologists, neuropsychologists, and other clinicians. In the Mathys-48 dataset, 20/48 donors had neither CERAD pathology nor Clinical AD diagnosis, 4 were diagnosed with AD but did not meet the CERAD pathology criterion, 6 met the CERAD pathology criterion but were not clinically diagnosed, and finally 18 met both criteria (Supplementary Fig. 2a). Thus, our second goal was to determine whether gene expression could distinguish cell-type-specific signatures of clinical AD diagnosis from those of Alzheimer’s neuropathological burden.

**Fig. 4:**
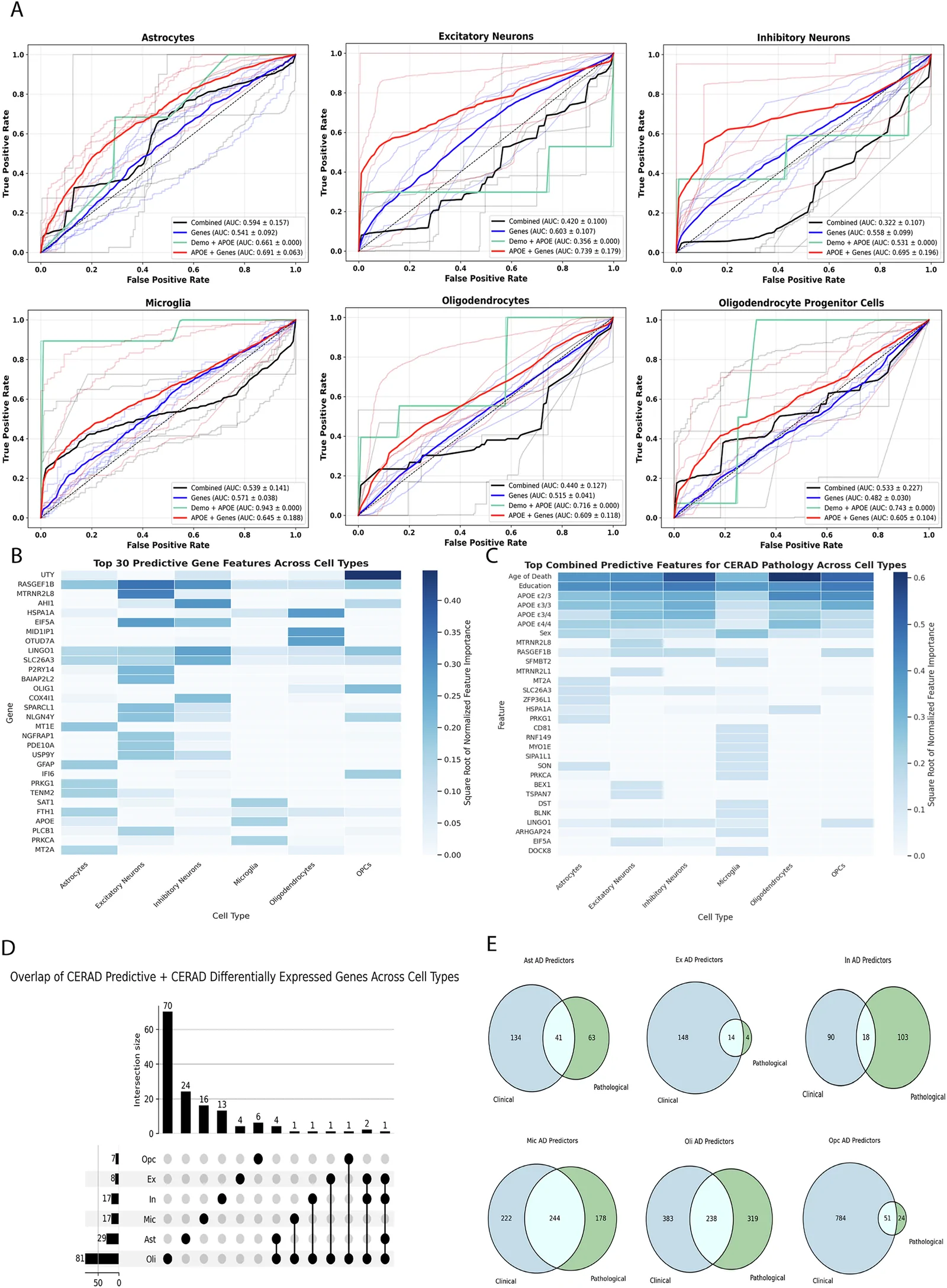
Predictive modeling of neuropathologic amyloid burden uncovers distinct and overlapping molecular signatures across cell types. **A** Receiver operating characteristic (ROC) curves summarizing the results from five independent runs of classification experiments performed on six major brain cell types: Astrocytes, Excitatory Neurons, Inhibitory Neurons, Microglia, Oligodendrocytes, and Oligodendrocyte Progenitor Cells. In this analysis, the classification task was to distinguish cells from individuals with high Alzheimer’s pathology (CERAD score 1 or 2) versus controls. For each cell type, classifiers were trained using four feature sets: (1) a combined set including demographic variables (age at death, sex, education), APOE genotype, and gene expression data (black curves); (2) demographic variables and APOE genotype only (red curves); (3) gene expression plus APOE genotype (green curves); and (4) gene expression only (blue curves). Mean area under the curve (AUC) values ± standard deviation are reported in the figure legends for each curve. Lighter lines represent individual splits; bold lines indicate mean performance. Higher AUC values reflect better discrimination of cells from high- versus low-pathology individuals. **B** Heatmap showing the relative importance (square-root-normalized feature importance) of the top 30 most predictive gene expression features identified for CERAD score classification, across each cell type. Darker blue indicates higher importance. Gene features were ranked by their mean importance across cross-validation splits and then displayed in order of aggregate contribution. Feature importance was square-root-normalized for display. **C** Heatmap showing the relative importance of the top 30 combined features (genes, APOE genotype, and demographic features) for CERAD score classification, across each cell type. This includes non-gene features (e.g., APOE ε2/ε3/ε4 status, age at death), highlighting their potential contributions relative to gene expression markers. Feature importance was square-root-normalized for display. **D** UpSet plot illustrating the overlap between the top predictive genes for CERAD score classification and differentially expressed genes (DEGs) identified between CERAD high and low pathology groups within each cell type. Vertical bars indicate intersection sizes (overlap), and connected dots indicate cell types contributing to each intersection set. This analysis highlights cell-type-specific overlaps and shared predictive signatures. **E** Venn diagrams summarizing the overlap between gene features predictive of CERAD score pathology status (blue) and gene features predictive of Alzheimer’s disease diagnosis (green), for each major cell type. Gene predictors were defined as features that appeared in at least two out of five cross-validation splits. The overlap highlights shared versus distinct molecular predictors used to discriminate pathology severity (CERAD) and clinical AD status across cell types. Source data are provided as a Source Data file.

To test this, we reran our analysis of the Mathys-48 dataset^7^, redefining outcomes by neuropathology rather than clinical diagnosis. Specifically, we classified moderate or frequent neuritic plaques (CERAD score 1-2) as AD, using this semiquantitative measure of plaque pathology as the outcome. Our classifiers showed that gene expression together with APOE genotype strongly predicted neuritic plaque pathology across cell types, similar to the clinical diagnosis of AD (Fig. 4A).

Across all cell types, APOE genotype and gene expression predicted neuritic plaque pathology (mean AUC range: 0.605 (Oligodendrocyte Progenitor Cells)−0.739 (Excitatory neurons) Supplementary Data 4). We found strong concordance between genes predictive of pathology-defined AD and clinical AD (Fisher’s q < 1.92e-20) (Supplementary Data 7), particularly in Microglia, with 244 overlapping predictors (OR = 106.7, 95% CI: 84.4-134.9), and in Oligodendrocytes, with 238 overlapping predictors (OR = 33.09, 95% CI: 27.2-40.27) (Fig. 4E, and Supplementary Data 7). These results suggest that in Microglia and Oligodendrocytes, molecular signals of amyloid pathology may be tightly coupled to clinical manifestation of disease^36,37^.

Although many predictors overlapped with clinical AD, their relative importance shifted in the pathology-based models. In Microglia, top predictors included *PRKCA*, *CD81*, and *APOE*. Differential expression analysis showed *PRKCA* was downregulated in AD, whereas *CD81* and *APOE* were upregulated, consistent with advanced amyloid pathology and altered immune-associated signaling (Fig. 4B, C and Supplementary Data 3, 9)^38^. Interestingly, the pathology-based models also highlighted genes that did not predict clinical AD, suggesting distinct molecular signals related to plaque pathology. For example, in Astrocytes we again observed *RASGEF1B* and *GFAP*, but *APOE* expression emerged in addition as a strong predictor. In contrast, *CLU*, while predictive of clinical AD, was not predictive of neuropathology, suggesting a potential bias toward cognitive AD phenotypes^39^. Together, these findings reveal that while clinical AD and AD neuropathology share many overlapping molecular programs, their divergence across specific genes highlights distinct biological processes underlying cognitive decline versus amyloid pathology in a cell type-specific manner.

### Transcriptional analysis of divergent clinical outcomes under matched neuropathology

Having shown that clinical and neuropathological predictors partially diverge, we next asked what distinguishes clinically symptomatic from resilient individuals among those with equivalent pathological burden. We performed restricted differential expression analyses of individuals with confirmed AD neuropathology, defined by CERAD scores of 1 or 2. We performed this analysis independently in two cohorts—Mathys-48 (24 neuropathology-positive: 18 with clinical AD, 6 without) and ROSMAP-48b (26 neuropathology-positive: 14 with clinical AD, 12 without), enabling a direct comparison between clinically symptomatic and resilient individuals matched for pathological burden.

In microglia, we identified a small set of genes showing consistent differential expression across the two cohorts, including APOE, TUBA1B, TNFRSF1B, PFKFB3, and ACSL1 (Supplementary Fig. 2b). Pathway enrichment analysis of these replicated microglial genes revealed overrepresentation of pathways related to inflammatory response, cytokine production, and regulation of cytokine signaling, consistent with activation of neuroimmune and inflammatory programs in clinically symptomatic individuals (Supplementary Fig. 2d).

In oligodendrocytes, a substantially larger set of genes exhibited consistent differential expression across cohorts. These included genes involved in cellular stress responses and metal homeostasis such as LAMP1^40^, MT3^41^, and RASGEF1B, among others (Supplementary Fig. 2c). Pathway analysis of replicated genes highlighted enrichment of pathways related to ion transport, cellular stress responses, protein localization, and cell cycle and cytokinesis regulation (Supplementary Fig. 2d). Together, these findings suggest that, even among individuals with similar neuropathological burden, distinct microglial inflammatory programs and oligodendrocyte stress- and transport-related transcriptional alterations are associated with progression to clinically symptomatic AD.

### Subcluster level expression improves AD prediction

**Fig. 5:**
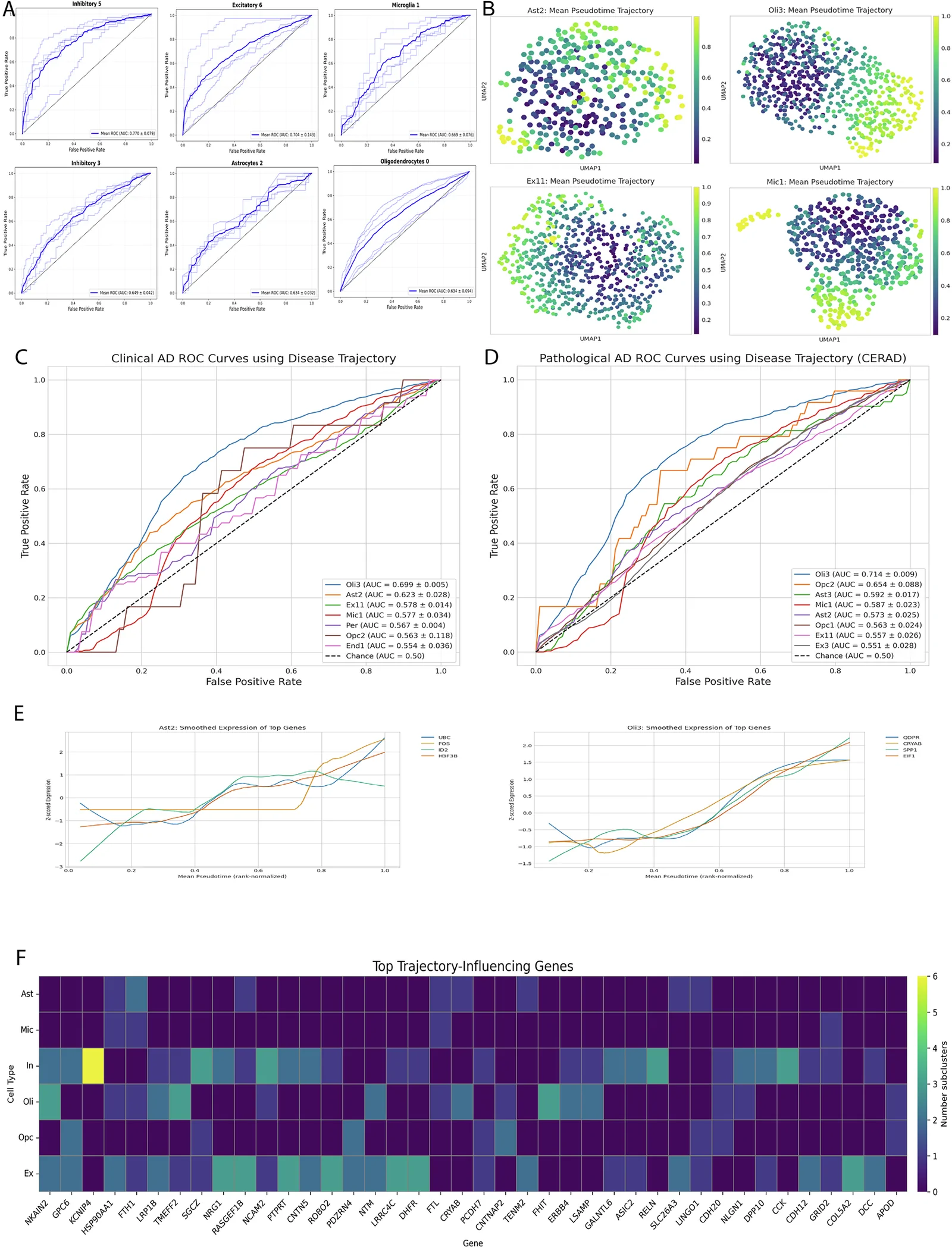
Subcluster-level predictive, trajectory, and trait enrichment analyses uncover fine-grained transcriptional programs in Alzheimer’s disease. **A** Receiver operating characteristic (ROC) curves summarizing subcluster-level classification of Alzheimer’s disease status using gene expression data alone. The six subclusters that exhibited the highest mean ROC performance among all subclusters analyzed. Mean area under the curve (AUC) values ± standard deviation across five cross-validation splits are indicated in the figure legends. Light blue lines represent ROC curves from individual splits, and bold lines indicate the averaged ROC curve per subcluster. Higher AUC values reflect better discrimination of Alzheimer’s disease cells from control cells within each subcluster. **B** Pseudotime trajectory visualizations for representative subclusters with high predictive performance or biologically relevant transcriptional signatures. For each subcluster, pseudotime was inferred using diffusion pseudotime (DPT), starting from root cells selected based on minimal amyloid pathology and minimal cognitive decline. Cells are plotted in UMAP space and colored by pseudotime values (color scale: dark purple to bright yellow), representing transcriptional progression from early to advanced disease states. **C** ROC curves summarizing the predictive capacity of pseudotime trajectories for distinguishing clinical AD versus control cells across significant subclusters. Pseudotime was inferred using diffusion pseudotime (DPT) initialized from root cells with a low amyloid pathology score and low cognitive impairment. **D** ROC curves summarizing the predictive capacity of pseudotime trajectories for distinguishing pathological AD versus control cells across significant subclusters. **E** Smoothed expression trajectories of top trajectory-influencing genes across pseudotime in Oligodendrocytes 3 and Astrocytes 2. **F** Heatmap showing top trajectory-influencing genes across major brain cell types. For each cell type, genes were identified based on their influence on pseudotime trajectories, with the top 1% of trajectory-associated genes selected within each subcluster. Color intensity indicates the number of subclusters (within a given cell type) in which a gene was ranked among the top 1% most trajectory-influential, reflecting the consistency of that gene’s association with transcriptional progression across subclusters. Source data are provided as a Source Data file.

Having identified cell-type-level predictors and their relationship to differential expression, we next asked whether finer transcriptional resolution, at the subcluster level, could improve prediction and reveal disease-associated trajectories. Predictive modeling at the subcluster level revealed Inhibitory 5 (In5) (top 3 marker genes: *CPLX3*, *TRPC3*, *KIT*) (mean AUC = 0.77 ± 0.079), Excitatory Neurons 6 (top 3 marker genes: *CA4*, *TIMP3*, *P2RY14*) (mean AUC = 0.704 ± 0.143), and Microglia 1 (top 3 marker genes: *RPL19*, *RPS11*, *RPL35*) (mean AUC = 0.669 ± 0.076) (Fig. 5A) as the most predictive cell type subclusters. At the subcluster resolution we saw that gene expression could predict clinical disease status in 31 out of 41 subclusters (mean AUC > 0.5), highlighting that localized gene expression patterns may reflect AD vulnerability (Fig. 5A, Supplementary Data 13). In Inhibitory Neurons 5, the top features were *RASGEF1B* and *PNISR* (Supplementary Data 13). We noted *LRRTM4*, which encodes leucine-rich repeat transmembrane protein 4, and *ATPB1* as top predictors in Ex6 (Supplementary Data 13). Taken together, these findings show that subcluster-level analyses can uncover transcriptional gradients overlooked at the broad cell-type resolution, and that focusing on these finer-grained states yields improved predictive performance, suggesting that disease vulnerability is better captured at the resolution of discrete transcriptional subclusters rather than whole cell types.

### Subcluster profiles reveal transcriptional gradients of ad progression

Applying the third branch of TriSCOPE, we performed diffusion pseudotime analysis^42,43^ to map transcriptional trajectories of both clinical and pathological AD within subclusters. Trajectories were constructed using highly variable genes selected independently of disease status or differential expression testing (Fig. 1B). To quantify the strength and breadth of disease-associated transcriptional trajectories, we evaluated the ability of subcluster-specific pseudotime to predict clinical AD diagnosis and neuropathological burden. Only a small subset of subclusters showed strong discrimination, with the most consistent signal observed in glial lineages—most notably an oligodendrocyte population (Oli3), which was predictive of both clinical and pathological endpoints (clinical AUC = 0.699, neuropathological *AUC* = *0.714*) (Fig. 5C, D), while weaker predictivity emerged across additional subclusters (Supplementary Data 14). To identify genes driving these disease-associated trajectories, we extracted the top trajectory-influencing features for each subcluster, finding *NKAIN2, LRP1B, HSP90AA1, FTH1, CRYAB*, and *SGCZ* across multiple neuronal and glial subclusters, and established AD genes in cell-type specific contexts (e.g., *APOE*/*GFAP* in astrocytes; *CLU* in excitatory neurons) (Supplementary Data 14). The discovery of these canonical genes suggests that the inferred trajectories recover known AD-associated expression programs while also highlighting additional candidate genes for follow-up.

### Integrated TriSCOPE identifies convergent Alzheimer’s disease programs

Applying three orthogonal analytical frameworks to single-cell data, our analyses illustrate the distinct types of disease-associated signals captured by each approach. Predictive modeling prioritizes genes that are conditionally informative when expression is modeled jointly across donors and covariates, differential expression captures marginal transcriptional shifts associated with disease status, and pseudotime inference identifies genes whose expression changes coherently along disease-associated trajectories (Supplementary Fig. 1f). Because each analytical axis captures a distinct mode of disease-associated signal in single-cell data, we used their intersection as a final integrative step to nominate cell-type–specific Alzheimer’s disease programs that are consistently detected across predictive, marginal, and trajectory-based analyses (Fig. 1D). These convergent genes include astrocytic stress and reactivity markers (GFAP, MT2A, MT3), neuronal signaling genes recurrently implicated in Alzheimer’s disease (RASGEF1B, LINGO1, NRG1), microglial regulators (HS3ST4, SYTL3), and oligodendrocyte-associated genes involved in myelination and metabolic support (APOD, OLIG1, SPP1), exemplifying high-confidence, cell-type–resolved molecular programs supported across all three orthogonal analytical paradigms.

### Independent validation of TriSCOPE

To evaluate the generalizability of TriSCOPE, we applied the full three-pronged framework, predictive modeling, donor-aware differential expression, and pseudotime inference, to the Seattle Alzheimer’s Disease Brain Cell Atlas (SEA-AD), an independent single-nucleus RNA-seq dataset comprising 1,395,601 nuclei from the prefrontal cortex of 83 donors (41 controls, 39 dementia, 3 neurotypical). SEA-AD cells were mapped to predefined cell-type annotations^7^ using a centroid-based approach, enabling direct comparison of gene-level findings across cohorts.

We first trained cell-type-specific classifiers on SEA-AD using the same modeling framework applied to ROSMAP. Consistent with our discovery analyses, models incorporating gene expression outperformed demographics-only baselines across cell types. In five of six major cell types, classifiers using APOE genotype and gene expression achieved the highest mean AUC: Microglia (0.738 ± 0.031), Inhibitory Neurons (0.709 ± 0.043), Excitatory Neurons (0.670 ± 0.039), Astrocytes (0.657 ± 0.138), and Oligodendrocytes (Genes-only: 0.660 ± 0.052). The sole exception was OPCs, where the combined model (demographics, APOE, and gene expression) achieved the best performance (0.704 ± 0.071), exceeding the APOE+Genes model (0.650 ± 0.106), suggesting that demographic covariates contribute additional predictive signal in this cell type within the SEA-AD cohort. Across all cell types, demographics and APOE genotype alone performed near chance (mean AUC range: 0.493–0.678), reinforcing that transcriptomic features are the primary drivers of disease classification.

To assess cross-cohort reproducibility of predictive gene selection, we quantified the overlap between ROSMAP-derived and SEA-AD-derived predictor sets for each cell type using Fisher’s exact tests against a background of 19,011 expressed genes. Significant overlap was observed in all six cell types: Microglia shared 81 predictive genes (OR = 7.95, *p* = 1.86 × 10^−^^39^), including APOE, ARL17B, BIN1, CTSB, FKBP5, and SPP1. Oligodendrocytes shared 11 predictors (OR = 23.67, *p* = 1.22 × 10^−^^10^), Astrocytes shared 7 (OR = 23.74, *p* = 7.17 × 10^−^^8^), OPCs shared 7 (OR = 6.98, *p* = 2.05 × 10^−^^4^), Excitatory Neurons shared 4 (OR = 39.74, *p* = 8.54 × 10^−^^6^), and Inhibitory Neurons shared 2 (OR = 16.19, *p* = 8.13 × 10^−^^3^). Notably, ARL17B was among the overlapping predictors in five of six cell types (Astrocytes, Microglia, Inhibitory Neurons, Oligodendrocytes, and Excitatory Neurons), reinforcing its role as a pan-cell-type predictor of clinical AD across independent cohorts (Supplementary Data 24).

Cross-cohort convergence was also observed at the level of differential expression. Donor-aware DE analysis in SEA-AD identified cell-type-specific DEGs that significantly overlapped with ROSMAP-derived DEGs across all six cell types (Fisher’s exact test, background = 19,011 genes). The strongest enrichment was observed in Oligodendrocytes (72 shared DEGs; OR = 6.33, *p* = 7.22 × 10^−30^) and Excitatory Neurons (155 shared DEGs; OR = 2.36, *p *= 5.02 × 10^−18^), followed by Microglia (19 shared; OR = 13.47, *p* = 1.94 × 10^−14^), Astrocytes (34 shared; OR = 5.55, *p* = 1.11 × 10^−13^), OPCs (10 shared; OR = 18.38, *p* = 1.17 × 10^−9^), and Inhibitory Neurons (27 shared; OR = 4.29, *p* = 3.36 × 10^−9^). Among shared microglial DEGs were APOE, SPP1, SRGN, CD81, PFKFB3, SNCA, and VSIG4, while shared OPC DEGs included HSP90AA1, OLIG1, OLIG2, and several metallothionein genes (MT2A, MT3) (Supplementary Data 24).

We next examined whether genetically anchored predictors that were not differentially expressed, genes identified through GWAS–eQTL colocalization and predictive modeling but invisible to univariate DE (predictive importance in ≥2/5 splits, PP.H4 > 0.8, but not differentially expressed), replicated in the independent SEA-AD cohort. ARL17B, the most prominent of these in our discovery analyses, was identified as a predictor in five cell types in SEA-AD (Astrocytes, Microglia, Inhibitory Neurons, Excitatory Neurons, and Oligodendrocytes), confirming that this genetically supported signal is reproducible across cohorts. Additionally, *JAZF1* replicated in Microglia as a colocalized predictor not identified by differential expression, providing further evidence that a subset of genetically anchored, predictive genes captures disease-relevant signal that is systematically missed by differential expression.

Finally, we performed pseudotime analysis in the SEA-AD cohort to evaluate replication of disease-associated transcriptional trajectories. Among subclusters, Ast1 achieved the strongest discrimination of neuropathological burden (CERAD AUC = 0.966), followed by Opc2 (CERAD AUC = 0.724) and Oli3 (CERAD AUC = 0.640). For clinical AD, Ast1 (AUC = 0.755), Oli3 (AUC = 0.649), and Ast3 (AUC = 0.601) showed the strongest pseudotime-based discrimination. Cross-cohort overlap of trajectory-influencing genes was strongest in Inhibitory Neurons (20 shared genes; OR = 64.60, *p* = 2.69 × 10^−27^) and Excitatory Neurons (14 shared genes; OR = 37.49, *p* = 7.85 × 10^−17^), with shared genes including *DCC*, *RELN*, *GRIA4*, *GRID2*, and *UNC5D* in Inhibitory Neurons, and *NRG1*, *ROBO2*, *RORA*, and *CDH13* in Excitatory Neurons (Supplementary Data 24). Cross-cohort trajectory overlap in Microglia and Oligodendrocytes did not reach statistical significance (*p* = 0.079 and *p* = 0.064, respectively), while no overlap was observed in Astrocytes or OPCs. Together, these results demonstrate that TriSCOPE identifies reproducible, cross-cohort disease programs across predictive modeling, differential expression, and trajectory inference, addressing concerns about generalizability beyond ROSMAP.

## Discussion

We identified convergent and cell-type-specific transcriptional programs associated with both clinical Alzheimer’s diagnosis and neuropathological burden across six major brain cell types and 41 subclusters. By integrating multivariate predictive modeling, donor-aware differential expression, pseudotime inference, and conditional GWAS–eQTL colocalization across ~4.2 million nuclei from 627 donors across independent cohorts, we mapped AD vulnerability, uncovering a class of genetically anchored, disease predictive genes whose relevance is invisible to differential expression. We further confirmed cross-cohort generalizability by replicating key findings across ROSMAP^7,44–46^, the South West Dementia Brain Bank^13^, and SEA-AD^15^ cohorts, with the latter reproducing results from all three TriSCOPE branches across cell types.

Applying TriSCOPE across increasing levels of cellular resolution revealed a structured increase in disease-discriminative signal, with modest performance at global levels and the strongest signal emerging within biologically coherent subclusters. This behavior reflects the multivariate nature of Alzheimer’s disease transcriptional programs, which are diluted at coarse resolution but become detectable when conditionally modeled within more homogeneous cellular states. A central strength of TriSCOPE lies in its use of supervised learning to prioritize a concise, stable set of disease-relevant genes that contribute jointly to disease discrimination, including genes that do not meet conventional univariate significance thresholds but are consistently informative in multivariate models. This capability is essential in high-dimensional single-cell data, where marginal testing alone cannot resolve redundancy among correlated transcripts or identify features that are conditionally informative given the broader transcriptional context. Empirically, only 3.9–24.6% of predictive genes overlapped with DEGs across cell types, and predictor rank showed weak correlation with log₂ fold change (Spearman ρ ≈ 0.02–0.21), showing that TriSCOPE captures disease-associated structure while compressing candidate lists relative to donor-aware DE alone, enabling downstream prioritization. We further ran simulations to characterize the mechanisms of this mismatch, including redundancy among correlated transcripts, nonlinear threshold effects, disease subgroup heterogeneity, and distributional changes without mean shifts.

This approach recovered well-established AD genes alongside less well-characterized candidates such as *ARL17B* across cell types and *IFI44L* in Microglia—illustrating how predictive modeling can refine candidate molecular programs of disease vulnerability beyond DE alone. Among pan-cell-type predictors, *ARL17B* emerged consistently across multiple cell types, aligning with growing evidence that the 17q21.31 MAPT inversion region exerts broad regulatory effects on brain phenotypes^47^. Although *ARL17B* itself is poorly characterized, it tracks with the inversion haplotype that also modulates *KANSL1* and *LRRC37A*^48^, genes repeatedly implicated in neurodegeneration and AD GWASs^49^. Prior work has similarly linked *ARL17B* expression in the frontal cortex to genetic risk and cortical thickness, supporting its interpretation as a haplotype-anchored transcriptional marker of disease vulnerability rather than a definitive causal effector^50^. We note that the 17q21.31 locus harbors a large-scale (~1 Mb) genomic inversion that suppresses recombination, producing unusually high linkage disequilibrium among SNPs throughout the region^48^, which may resist even careful conditional colocalization analysis. ARL17B’s near-complete colocalization (PP.H4 ≈ 0.99) should therefore be interpreted cautiously, as it may reflect the broad regulatory influence of the inversion haplotype rather than implicating ARL17B as a singular causal effector. Consistent with multi-omic support for genes at this locus, KANSL1 has been linked to AD-associated DNA methylation changes, and PICALM shows convergent methylation and expression evidence^51,52^. In contrast to ARL17B’s pan cell-type signal, IFI44L emerged as a microglia-specific predictor, reflecting its role as an interferon-stimulated gene and marker of innate immune activation that points to a cell-type-restricted program of interferon-driven microglial dysfunction.

A key insight from our analysis is the partial dissociation between transcriptional predictors of clinical AD diagnosis and those associated with neuropathological burden, which is often obscured by studies that rely on phenotypes that blend clinical and pathological definitions^53^. Leveraging both predictive modeling and differential expression, we observed the greatest concordance between clinical and neuropathological signatures in microglia and oligodendrocytes, consistent with neuroinflammatory and myelination-related pathways contributing to both cognitive decline and pathological progression. Furthermore, when restricting to neuropathology-matched individuals, differential expression revealed distinct microglial inflammatory programs and oligodendrocyte stress-related alterations that distinguish clinically symptomatic from resilient individuals, reinforcing the central role of these cell types in mediating the relationship between pathological burden and clinical outcome. These findings underscore the importance of phenotype-specific modeling when interpreting molecular signatures of Alzheimer’s disease.

Conditional GWAS-eQTL colocalization, restricted to genes prioritized by predictive modeling, identified a focused set of genes whose transcriptional regulation and inherited AD risk converge in specific cellular contexts. Across cell types, 27 gene–cell type pairs were genetically anchored (PP.H4 ≥ 0.8) and predictive of clinical AD, yet not differentially expressed—a class of disease candidates systematically missed by standard transcriptomic analyses. These included JAZF1, SYK, and RIN3 in microglia, PLEKHA1 in oligodendrocytes, ERC2 across three cell types, USP6NL in microglia, and EGFR in OPCs.

At the subcluster level, pseudotime inference identified recurrent trajectory-influencing genes including NKAIN2, LRP1B, HSP90AA1, FTH1, and CRYAB across neuronal and glial populations, with established AD genes appearing in cell-type-specific contexts (APOE/GFAP in astrocytes, CLU in excitatory neurons). Integration across all three branches nominated a convergent set of high-confidence programs — including astrocytic metal homeostasis and oxidative stress markers (MT2A, MT3), neuronal signaling genes (RASGEF1B, LINGO1, NRG1), microglial regulators (HS3ST4, SYTL3), and oligodendrocyte genes involved in myelination and metabolic support (APOD, OLIG1, SPP1). The independent recovery of well-characterized AD-associated genes such as *RASGEF1B* and *LINGO1* in neurons^7^, supports the biological validity of the TriSCOPE framework and suggests that less-characterized predictions may similarly reflect meaningful disease biology.

Our findings also have translational implications for therapeutic development. Using TriSCOPE, we uncovered several genes and pathways relevant to Alzheimer’s disease, including microglia-specific neuroinflammatory genes (IFI44L, TNFRSF1B, SRGN, PLXDC2, CD81) as well as genes involved in protein homeostasis and glycosylation across cell types (HSPA1A, CRYAB, PFKFB3, SGCZ, LAMP1), the latter set linking to evidence that N-glycosylation contributes to tau hyperphosphorylation^54^, a hallmark of AD. Systematic annotation against DrugBank revealed that multiple TriSCOPE-prioritized candidates are already linked to existing pharmacologic compounds, such as EGFR and HSP90AA1, which map to drugs with AD-relevant mechanisms. EGFR inhibitors modulate microglial neuroinflammatory responses in preclinical AD models^55^, while HSP90 inhibitors promote clearance of misfolded tau and amyloid-beta^56^, and TNFRSF1B, which maps to TNF-receptor-targeting compounds relevant to neuroinflammatory signaling in AD^57^, highlighting opportunities for future mechanistic studies.

Our study has several limitations: firstly, our framework is predictive in design, but its primary aim is feature discovery rather than clinical prognostication. The AUCs reflect the use of post-mortem, cross-sectional data, where prediction cannot be the endpoint; instead, the value lies in the identification of key genes and cell states that may reflect AD vulnerability independent of known risk factors, and thus warrant further investigation. Importantly, the cross-sectional nature of the data precludes inference about temporal ordering, such that we cannot determine whether the identified transcriptional signatures precede disease onset, track disease progression, or represent late-stage reactive responses to neuropathology. Longitudinal inference, approximated through integration with longitudinal clinical or imaging trajectories or via matched multi-region sampling within the same individuals across brain regions affected at different stages of Alzheimer’s disease, would better align molecular variation with disease stage, potentially strengthening signal-to-noise and improving predictive discrimination. As such, we believe that perturbation experiments will be essential to disentangle whether these transcriptional signatures represent protective adaptations, pathogenic drivers, or reactive epiphenomena. These perturbation experiments, when coupled with our subcluster-level findings, open up avenues for more targeted therapeutic studies. Additionally, we chose to focus on the prefrontal cortex in AD, since it represents a brain region most directly responsible for executive function, where we expected to see transcriptional changes associated both with Alzheimer’s neuropathology and cognitive decline, however, this focus may limit the generalizability of our findings to other brain regions that are affected earlier or differently over the course of disease progression, such as the hippocampus, entorhinal cortex, or subcortical nuclei. Lastly, our colocalization results do not assert direct causality, but rather provide evidence that AD-associated genetic variants and gene expression changes share a common genetic origin; causal resolution will require perturbational validation.

Our work reveals that Alzheimer’s disease vulnerability is encoded in cell-type-specific transcriptional programs that are often invisible to univariate differential expression but emerge through the convergence of multivariate prediction, genetic anchoring, and trajectory inference. By identifying a class of genetically supported disease genes systematically missed by standard analyses, and demonstrating their reproducibility across independent cohorts, this study provides both a resource for mechanistic follow-up and evidence that the molecular architecture of AD is richer than any single analytical lens can capture.

## Methods

### Ethics

This study is an analysis of de-identified, previously published human single-nucleus RNA-sequencing data and the associated clinical and neuropathological metadata. No human participants were recruited, no new human tissue or data were collected, and no participant compensation was provided by the present study. Because the analysis used only de-identified data that were not individually identifiable to the investigators, it did not constitute human subjects research and institutional review board approval was not required. Access to controlled-access datasets was obtained under an executed Data Use Agreement between Harvard Medical School and Sage Bionetworks.

All contributing studies obtained ethical approval and written informed consent from participants or their legal representatives prior to data collection. The ROSMAP-derived cohorts were collected under protocols approved by the Institutional Review Board of Rush University Medical Center^14^. The Lau-21 and Seattle Alzheimer’s Disease Brain Cell Atlas cohorts were collected under the ethical approvals described in their original publication^13,15^. All contributing studies complied with all relevant regulations governing the use of human study participants and were conducted in accordance with the criteria set out in the Declaration of Helsinki.

### Sex and gender

This study analysed data from 627 donors: Mathys-48 (*n* = 48; 24 male, 24 female), Lau-21 (*n* = 21; 14 male, 7 female), ROSMAP-48b (*n* = 48; 24 male, 24 female), ROSMAP-427 (*n* = 427; 212 male, 215 female) and SEA-AD (*n* = 83; 35 male, 48 female), giving 309 male and 318 female donors overall. Sex was recorded for all 627 donors. Age at death spanned the 60–62 to 90+ year bands across cohorts. Sex was considered in the study design: it was included as a demographic feature in the combined predictive models, and model interpretation was additionally stratified by sex (Supplementary Fig. 4). Sex was recorded by the contributing cohorts during their original data collection; the present study neither collected nor assigned this information, and the method of ascertainment is described in the original cohort publications. Gender was not available in the metadata released by any contributing cohort and could therefore not be analysed. Donor-level sex is reported disaggregated in Supplementary Data 15.

### Data source and cohort selection

We analyzed previously published single-nucleus RNA sequencing (snRNA-seq) dataset^7^ generated by Mathys et al. (2019), derived from post-mortem dorsolateral prefrontal cortex (PFC) tissue collected as part of the Religious Orders Study and Rush Memory and Aging Project (ROSMAP)^14^. We chose to use the prefrontal cortex for its important role in cognitive control and executive function. The original dataset included 48 individuals (26 with no clinical Alzheimer’s diagnosis, and 22 with clinical Alzheimer’s diagnosis)^14^, balanced by age, sex, and educational attainment. Detailed clinical, cognitive, and neuropathological assessments were obtained longitudinally and at autopsy (see *Clinical and Neuropathological Definitions of Alzheimer’s Disease*)^7^. When classified based on CERAD pathology, there were 24 individuals with AD-like pathology and 24 individuals with non-AD pathology (Supplementary Fig. 2a). All participants provided informed consent, and protocols were approved by the Institutional Review Board of Rush University Medical Center.

In addition to the primary Mathys-48 cohort, we incorporated four complementary single-nucleus and single-cell RNA-seq datasets to support robustness analyses and external validation. First, we analyzed two additional ROSMAP snRNA-seq releases derived from the same dorsolateral prefrontal cortex region, comprising cohorts of 48 and 427 donors, respectively. The second 48-donor ROSMAP cohort included 22 individuals classified as Alzheimer’s disease by CERAD neuropathology criteria and 15 individuals with a clinical Alzheimer’s diagnosis, providing an intermediate dataset bridging the original Mathys cohort and the larger ROSMAP release. The 427-donor ROSMAP dataset substantially increased statistical power for donor-aware differential expression analyses and reflected a broader spectrum of neuropathological and clinical heterogeneity. We also analyzed the Lau et al. dataset^13^, which includes single-cell RNA-seq profiles from 21 individuals (12 Alzheimer’s disease cases and 9 controls), generated using an independent experimental pipeline and cohort design. We then utilized the Skene/Murphy reprocessed version of the Mathys et al. 2019 dataset, which applies updated quality control, normalization, and cell-type annotation procedures to the original 48-donor cohort.

Finally, we analyzed single-nucleus RNA-seq data from the Seattle Alzheimer’s Disease Brain Cell Atlas (SEA-AD), comprising 1,395,601 nuclei from the prefrontal cortex of 83 donors (41 controls, 39 with dementia, 3 neurotypical), generated by the Allen Institute for Brain Science. Nuclei-by-gene matrices with normalized expression values were obtained from the SEA-AD Open Data Registry on AWS (sea-ad-single-cell-profiling). Clinical AD status was defined using the alzheimers_or_control variable derived from cognitive status (Dementia vs. No dementia), analogous to the ROSMAP DCFDX-based clinical classification. For neuropathological classification, we used the Overall AD neuropathological Change (ADNC) score, a composite measure integrating CERAD neuritic plaque density, Braak NFT stage, and Thal amyloid phase under the NIA-AA 2012 consensus framework. Donors with ADNC ratings of High or Intermediate were classified as neuropathology-positive, analogous to CERAD scores of 1–2 in ROSMAP. The same clinical and neuropathological definitions were applied consistently across predictive modeling, differential expression, and pseudotime analyses in the SEA-AD cohort.

### Single-nucleus RNA-seq preprocessing and quality control

For all analyses, we used the author-released, publication-grade processed gene-count matrices obtained from Synapse (Synapse ID: syn18485175) and did not reprocess raw FASTQs. Specifically, we obtained the processed gene-count matrix, consisting of an initial set of 17,926 genes profiled across 75,060 nuclei. In the original preprocessing, mitochondrial content was not filtered using a fixed percentage threshold; instead, a data-driven approach was applied in which a mitochondrial ratio (mitochondrial reads divided by the number of detected genes) was computed, and k-means clustering (k = 2) was used to classify nuclei into high- and low-mitochondrial groups, with nuclei in the high-mitochondrial cluster excluded. We retained this author-defined filtering to ensure consistency with prior analyses and to avoid introducing batch effects through partial reprocessing.

We applied minimal additional quality control to exclude nuclei with low gene detection or missing metadata, resulting in a final dataset comprising 17,926 genes across 70,634 high-quality nuclei. Cell-level metadata, including demographic variables and clinical annotations (e.g., age at death, sex, APOE genotype), were integrated using unique barcode identifiers. Post-mortem interval (PMI) did not differ significantly between AD and control individuals (FDR-adjusted *p* = 0.388), indicating comparable tissue preservation quality across diagnostic groups.

For the Lau-21, ROSMAP-48b, ROSMAP-427, and SEA-AD datasets, we likewise used author-released, publication-grade processed matrices and did not reprocess raw FASTQs, re-run alignment, or re-derive the original nucleus-level quality control. The Lau-21 cohort was obtained as CellRanger-processed per-sample count matrices from Gene Expression Omnibus accession GSE157827 and restricted to the 17,110 gene symbols shared with the discovery matrix. ROSMAP-48b and ROSMAP-427 were obtained as per-cell-type objects carrying the original authors’ annotations. SEA-AD comprises 1,395,601 nuclei from 83 donors, of which the three neurotypical reference donors were excluded from downstream analysis. In each case, analyses used the raw integer counts supplied in the release rather than any pre-normalized values.

### Clinical and neuropathological definitions of alzheimer’s disease

We used two definitions of Alzheimer’s disease throughout our study, *clinical* Alzheimer’s diagnosis and *neuropathological* Alzheimer’s diagnosis. For our clinical diagnosis, we used the clinical diagnosis of cognitive status (DCFDX) rendered at the patient’s last visit via uniform, structured, clinical evaluation, including a battery of 19 cognitive tests scored by computer, clinical judgment by a neuropsychologist, and diagnostic classification by a clinician. Using DCFDX, each patient is scored 1-6, with values of 4 or 5 corresponding to Alzheimer’s Dementia and a value of 6 corresponding to other primary causes of dementia, with no clinical evidence of Alzheimer’s dementia. In our study, we used only scores of 4 or 5 to define clinical Alzheimer’s disease, treating other cases of “other primary cause of dementia, no clinical evidence of Alzheimer’s dementia”^19^ as controls. For neuropathological based Alzheimer’s diagnosis, we used CERAD score which is a semiquantitative measure of neuritic plaques. For neuropathological Alzheimer’s, we required CERAD scores of 1 and 2, corresponding to frequent neuritic plaques (definite AD) in one or more neocortical regions and AD required moderate (probable AD), respectively^58^.

### Cell type annotations across datasets

For the Mathys-48 dataset, we used the available Broad cell type annotations (e.g., microglia, astrocytes, excitatory neurons) that were assigned using canonical marker gene expression profiles by Mathys et al. To further resolve cellular heterogeneity, we utilized subcluster labels from Mathys et al. For the ROSMAP-48b and ROSMAP-427 datasets, we used the broad cell-type annotations released with each dataset, which were assigned by the original authors using canonical marker gene expression profiles. No additional clustering or re-annotation was performed. In the ROSMAP-48b and ROSMAP-427 releases the authors provide an immune-cell compartment rather than a separately annotated microglial population. We used this immune-cell compartment in place of microglia, and refer to it as microglia throughout.

For the Lau et al. dataset, we started with the single-nucleus gene expression matrix which we preprocessed and clustered using the Scanpy (v.1.10.3) Python package. The log-normalized expression matrix, containing 172,514 nuclei and 17,110 genes, was used to construct an AnnData object without further normalization or log transformation. To reduce dimensionality while preserving biologically informative variability, we selected the top 5000 highly variable genes (HVGs) using the ‘seurat’ flavor, which prioritizes genes based on normalized dispersion and mean expression. Total counts per nucleus (approximated by the sum of inverse-log-transformed expression values) and the proportion of mitochondrial gene expression per nucleus were regressed out using Scanpy’s pp.regress_out function to control for potential technical variation related to library size and cell quality, followed by scaling each gene to unit variance and zero mean. Principal component analysis (PCA) was performed on the HVGs, and the top 50 principal components were used to construct a k-nearest neighbors (kNN) graph with k = 30 neighbors. A two-dimensional t-distributed stochastic neighbor embedding (t-SNE) was computed using the top 10 PCs to visualize the manifold structure of the cells. Cells were clustered using the Louvain algorithm with a resolution parameter of 1.0, resulting in unsupervised cell clusters. For each cluster, we identified differentially expressed genes using a variance-adjusted t-test implemented in Scanpy’s tl.rank_genes_groups, comparing each cluster to all remaining cells. We selected the top 500 marker genes per cluster to perform statistical enrichment analysis against curated cell-type marker gene sets. Specifically, we evaluated the overlap between each cluster’s top marker genes and cell-type-specific marker lists for Astrocytes, Oligodendrocytes, Oligodendrocyte progenitor cells, Microglia, Excitatory neurons, and Inhibitory neurons. These marker lists were assembled from published high-confidence marker genes reported in Ianevski et al. and Lake et al. ^59,60^, and further refined by including canonical markers widely used in the Alzheimer’s disease single-nucleus RNA-seq literature (Mathys et al.; Lau et al.). Cluster assignments were further confirmed by systematically evaluating marker gene expression across clusters using dot plots and t-SNE overlays. Overlap was evaluated using a hypergeometric test (one-sided Fisher’s exact test), with enrichment *p* values calculated per cell type and adjusted across all tested cell types within each cluster. The cell type with the most significant enrichment *p* value was assigned as the label for the corresponding cluster. We compared our annotations to those reported in Lau et al. (2020) and found 90% concordance in annotations. To assess annotation robustness, we compared our cluster-derived cell-type labels to independent assignments obtained using a supervised marker-based approach using the marker gene annotations published by Lau et al. In the Lau-based approach, each cluster’s mean expression profile was scored against an external marker gene list, and cell types were assigned to clusters based on maximal cumulative marker expression. We observed high concordance between the two annotation strategies, with 155,278 out of 172,514 nuclei (90.0%) showing consistent labeling.

For the SEA-AD dataset, broad cell-type labels were assigned by direct lookup of the author-assigned Subclass annotation, without clustering, marker scoring, or correlation-based reassignment. The nine glutamatergic subclasses (L2/3 IT, L4 IT, L5 IT, L5 ET, L6 IT, L6 IT Car3, L6 CT, L6b, and L5/6 NP) were mapped to excitatory neurons and the nine GABAergic subclasses (Pvalb, Sst, Sst Chodl, Vip, Lamp5, Lamp5 Lhx6, Sncg, Chandelier, and Pax6) to inhibitory neurons, reproducing the Class annotation released with the dataset. Astrocyte, Oligodendrocyte, and OPC were mapped to their corresponding broad classes, and Microglia-PVM was mapped to microglia. Endothelial and VLMC nuclei fall outside the six classes modeled here and were excluded.

Within these SEA-AD broad cell types, subcluster annotations were assigned using a centroid-based correlation approach that enabled direct comparison with Mathys et al. (2019) subcluster definitions. For each of the 41 subclusters defined by Mathys et al., we computed a centroid expression vector as the mean log-normalized expression across all cells belonging to that subcluster in the Mathys reference matrix. Each SEA-AD cell was then assigned to the subcluster whose centroid had the highest Pearson correlation with the cell’s expression vector, computed over all genes shared between the two datasets. This correlation-based approach uses the full shared transcriptome rather than a small marker gene set, making it robust to differences in marker gene expression magnitude between datasets. Assignment was performed within each broad cell type (Astrocytes, Excitatory Neurons, Inhibitory Neurons, Microglia, Oligodendrocytes, and OPCs), such that each SEA-AD cell was compared only to subclusters within its pre-annotated broad cell type. Assignment confidence was quantified as the margin between the top-ranked and second-ranked centroid correlations for each cell.

### Predictive model benchmarking and negative controls

To assess whether TriSCOPE’s predictive performance and downstream feature prioritization depended on a specific classifier choice, we benchmarked multiple commonly used supervised learning architectures for tabular gene-expression data. Specifically, we evaluated gradient boosted decision trees (GBM), LASSO-regularized logistic regression, elastic net–regularized logistic regression, and random forest classifiers across six major brain cell types.

All benchmarking experiments were conducted using an identical modeling pipeline. The only difference between benchmarked models was the choice of estimator, implemented by changing a single model argument within the same AutoML framework. This design ensured that any observed differences in performance or feature rankings reflected model architecture rather than differences in preprocessing or evaluation protocol. For each cell type, models were trained on the same gene-expression features, using the same donor-aware train/test splits, feature filtering, recursive feature elimination, class weighting, and cross-validation strategy. To assess whether the predictive performance and downstream feature prioritization of TriSCOPE depended on a specific choice of classifier, we evaluated four commonly used tabular learning architectures for gene-expression–based prediction of clinical Alzheimer’s disease (AD) across six major brain cell types: gradient boosted decision trees (GBM), LASSO-regularized logistic regression, elastic net–regularized logistic regression, and random forest classifiers. All models were trained and evaluated using identical preprocessing, donor-aware cross-validation splits (five splits per cell type), and feature inputs, enabling a controlled comparison of model behavior. Briefly, GBMs model non-linear interactions through additive ensembles of decision trees and are widely regarded as state-of-the-art for structured, tabular data. LASSO regression applies L1 regularization to perform sparse feature selection, favoring a small subset of predictive genes. Elastic net combines L1 and L2 regularization, balancing sparsity and stability in the presence of correlated predictors. Random forests aggregate multiple decorrelated decision trees and are robust to noise, but may dilute feature importance when signals are weak or distributed. Across all six cell types, each architecture achieved predictive performance above chance when trained on gene expression alone, with mean AUCs ranging from ~0.54 to 0.66 depending on cell type and model (Supplementary Data 16). Performance variability across architectures within a given cell type was modest, indicating that the disease signal was not driven by a single modeling choice.

To establish a conservative lower bound on predictive performance and to exclude spurious signals arising from donor structure or class imbalance, we additionally performed a negative-control experiment in which disease labels were permuted at the donor level in the training set only. In this control, all preprocessing, feature selection, and model training steps were identical to the primary analysis, but training set donor-level labels were randomly reassigned prior to training. As expected, models trained on permuted labels achieved below-chance performance (AUC < 0.5) across all cell types, consistent with the absence of a learnable disease signal under label randomization.

### Primary cohorts and analytical strategy

We performed de novo predictive modeling, donor-aware differential expression (DE), and pseudotime analyses across a total cohort comprising 627 unique individuals. Predictive modeling was conducted using the Mathys-48 dataset (*n* = 48 donors), the Lau-21 dataset (*n* = 21 donors), the ROSMAP-427 dataset (*n* = 427 donors), and the SEA-AD dataset (*n* = 83 donors), while donor-aware random-effects DE analyses were performed on the Mathys-48 dataset, two additional ROSMAP single-nucleus RNA-seq releases (ROSMAP-48b, ROSMAP-427) comprising *n* = 48 and *n* = 427 donors, respectively, and the SEA-AD dataset. Pseudotime analyses were performed on the Mathys-48 dataset and independently replicated in the SEA-AD dataset using subcluster assignments derived from centroid-based mapping (see Cell type annotations across datasets). To further assess robustness, we performed concordance analyses between TriSCOPE-derived predictive gene rankings and differential expression results from four additional independent Alzheimer’s disease single-cell and single-nucleus RNA-seq studies, comprising a total of 94 additional donors. These analyses evaluated rank concordance and overlap of disease-associated genes across studies generated using distinct experimental and analytical pipelines.

### TriSCOPE predictive modelling

We trained separate classifiers for each major cell type using normalized gene expression and evaluated their ability to predict Alzheimer’s status based on both Alzheimer’s diagnosis and CERAD neuropathological scoring. We trained on a variety of data modalities, including gene expression data and cell-level metadata variables: sex, education, age at death, and APOE genotype. We also tested various combinations of these data, including a combined model (gene expression data and all metadata variables), a gene expression data-only model, a metadata model (sex, education, age at death, and APOE genotype), and a gene expression and APOE genotype model. We also predicted on two sets of target variables, both Alzheimer’s diagnosis and CERAD pathology, using the National Institute on Aging’s guidelines for pathology-based diagnosis to group samples with CERAD scores of 1 and 2 as AD and scores of 3 and 4 as controls^61^. We took strict steps to ensure no data leakage in any of our predictive models, this including grouped train/test splits by donor in all models. We ensured that all train-test splits were done sample wise, ensuring that all cells from a given sample were in either the training or testing data, but were not included in both. Every predictive model was run across 5 different random seeds, with the train-test splits being determined by the random seed. All presented findings were calculated using data from across these 5 splits to ensure robustness.

We developed a supervised machine learning framework to predict Alzheimer’s disease (AD) status at the single-cell level. All analyses were performed in Python (v3.9.21) utilizing the FLAML AutoML library (v2.2.0), scikit-learn (v1.6.1), and custom scripts. Single-nucleus RNA-sequencing data were processed to generate a cell-by-gene expression matrix, which was normalized and log-transformed prior to analysis. Metadata, including APOE genotype and clinical annotations, were integrated at the single-cell level using matching unique cell barcodes (TAGs). Post-mortem interval (PMI) was assessed as a potential confounder in both the Mathys et al. and Lau et al. datasets using non-parametric tests and violin plots (Supplementary Fig. 3d). In both cohorts, PMI did not differ significantly between AD and control groups (FDR-adjusted *p*  =  0.388 and *p*  =  0.0949, respectively). Because PMI was constant across all cells from a given donor, and not associated with AD status, it was excluded from the predictive modeling framework to avoid overfitting and pseudoreplication. APOE genotype was encoded as a one-hot categorical variable. We performed a stratified train-test split at the donor level (80% train, 20% test) to prevent data leakage and to ensure that cells from the same individual did not appear in both sets. Stratification was performed jointly by AD status and sex to preserve class balance. Within only the training set data, we applied recursive feature elimination with cross-validation (RFECV) using a logistic regression estimator to select an optimal subset of informative genes, thus ensuring no data leakage. Genes with low mean expression or high sparsity were excluded prior to feature selection. After cell-type–specific expression and missingness filtering, the final gene sets used for modeling comprised 2971 genes in astrocytes (Ast), 1,339 genes in microglia (Mic), 3824 genes in oligodendrocyte precursor cells (Opc), 1751 genes in oligodendrocytes (Oli), 5637 genes in inhibitory neurons (In), and 7591 genes in excitatory neurons (Ex). After finalizing the gene set, expression data were merged with metadata features for each cell, and non-informative metadata variables were excluded to ensure model stability. The final feature set included the top-ranked genes along with APOE genotype encodings. We trained a gradient boosting classifier, LightGBM (v4.5.0), using FLAML, adjusting the time budget based on the number of variables per training run. Model optimization was guided by log-loss and cross-validated using grouped folds defined by donor identity to further mitigate batch and individual-level confounding. To account for class imbalance, inverse-frequency sample weights were incorporated during training. Prediction probabilities on training and test cells were computed, and an optimal decision threshold was determined by maximizing the F1 score on the training set based on the precision-recall curve. Evaluation metrics included area under the receiver operating characteristic curve (ROC AUC), average precision, accuracy, recall, precision, F1 score, and Matthews correlation coefficient. To evaluate feature importance and robustness, we additionally performed incremental retraining using the top 1 to 25 features, assessing test performance at each step. Prediction probabilities, classification outcomes, and corresponding metrics were saved for downstream interpretability analyses, including subcluster-specific misclassification and differential expression assessments. External validation was performed using an independent snRNA-seq dataset comprising 12 AD patients and 9 controls (totaling 172,514 nuclei across microglia, oligodendrocytes, excitatory neurons, inhibitory neurons, and astrocytes). Validation analysis included two steps: the first was fitting the predictive models trained on the Mathys et al. dataset (to study generalizability of predictive modeling across cohorts) and the second part including rerunning our entire pipeline in order to independently discover which features were most important in the Lau et al. dataset.

Validation analyses replicated the full predictive modeling workflow, including cell-type assignment, gene expression normalization, feature selection, and classifier training and evaluation. All performance metrics were calculated at the single-cell level, with train/test splits stratified and grouped by donor to avoid any cell from a given donor appearing in both sets. Cell-level metrics were reported because the biological question centers on detecting disease-associated states of individual cells. Donor-level aggregation would obscure within-donor heterogeneity, which is the central focus of our study.

For datasets with large per-donor cell counts (SEA-AD, ROSMAP-427), we applied per-donor downsampling prior to model training to ensure computational tractability and to control for donor-level sampling imbalance, whereby donors contributing disproportionately many cells could otherwise dominate model fitting. In SEA-AD, Excitatory and Inhibitory Neuron populations were downsampled to 1000 cells per donor for all gene-expression-containing model configurations. In the ROSMAP-427 cohort, cells were downsampled to 750 cells per donor; for cell types where this threshold exceeded available memory on the HMS HPC (Excitatory Neurons, Inhibitory Neurons, and Oligodendrocytes), a threshold of 400 cells per donor was used. All downsampling was performed independently within each of the five cross-validation splits using distinct random seeds.

### Pathway analysis of predictive and differentially expressed gene features

To interpret the biological relevance of genes predictive of Alzheimer’s disease (AD) status, we performed pathway enrichment analysis using gene rankings derived from our cell type–specific LightGBM classifiers. For each major cell type (Astrocytes, Excitatory Neurons, Inhibitory Neurons, Microglia, Oligodendrocytes, and OPCs), we trained five independent models on randomized train/test splits. Feature importance scores from each split were extracted and normalized by the total non-zero importance values. For each gene, we computed the mean normalized importance across splits and retained genes with non-zero importance in at least two out of five splits. Non-gene features (e.g., demographic covariates or APOE encodings) were excluded using name-based filtering. We ranked predictive genes by their average normalized importance (using all genes with a non-zero feature importance in at least 2 splits) and for DEGs we performed gene set enrichment analysis (GSEA) using the prerank function in the GSEApy Python package (v1.1.9). Enrichment was conducted against the GO_Molecular_Function_2023 gene set library^62^. Default parameters were used (minimum term size = 5, maximum = 1000), and a fixed random seed ensured reproducibility. The resulting pathways per cell type were visualized as barplots of –log₁₀(FDR). This same process was conducted for the differentially expressed genes where applicable.

### SHAP-based model interpretation stratified by APOE genotype and sex

To identify the features most predictive of Alzheimer’s disease (AD) classification across distinct genetic risk backgrounds, we applied SHAP (SHapley Additive explanations) (v0.48.0) analysis to an integrated model trained on both gene expression and donor-level demographic features. This approach enabled us to dissect model behavior at single-cell resolution while accounting for individual-level covariates. For each major cell type (Astrocytes, Excitatory Neurons, Inhibitory Neurons, Microglia, Oligodendrocytes, and OPCs), we analyzed the output of five independently trained models, each derived from a stratified train-test split using a LightGBM classifier implemented via FLAML. Models were trained using both transcriptomic features (*n* = 17,926 genes) and demographic metadata (age at death, sex, years of education, and one-hot encoded APOE genotype).

Unlike standard model interpretation methods that report global feature importance values from models trained solely on gene expression, our SHAP-based approach leverages a game-theoretic framework to compute the marginal contribution of each feature to the prediction for each individual cell. This allows for fine-grained, interpretable attribution of prediction decisions, enabling biologically meaningful comparisons across subgroups. To interrogate potential APOE-specific transcriptional programs, we stratified all cells into three mutually exclusive genotype groups based on their donor’s APOE genotype: non-carriers of ε4 (e.g., ε2/3, ε3/3), heterozygous ε4 carriers (ε3/4), and homozygous ε4/ε4 carriers. For each group, we computed SHAP values on the subset of cells belonging to that group using the full model trained on all features. SHAP values were computed using TreeExplainer on the LightGBM booster model (.booster_) and then aggregated in two ways: Cell-Level Gene SHAP Scores: For gene expression features, we computed the absolute SHAP value for each gene in each cell and averaged across all cells in the group. This highlights the genes that most consistently influenced AD predictions within each genotype-defined subgroup. Donor-Level Metadata SHAP Scores: For demographic covariates (age, sex, education, APOE one-hot features), we computed the absolute SHAP values per cell and aggregated these by donor, then averaged across donors in each group. This strategy controls for donor-level sampling imbalance and isolates the contribution of metadata in shaping the model’s behavior. We repeated an identical process across male and female labels in order to derive SHAP plots stratified by sex.

Features were only retained for visualization and downstream analysis if they had non-zero SHAP variance across the five model splits and positive mean SHAP values, ensuring robustness and biological interpretability. Bar plots of the top metadata drivers (per donor) and gene drivers (per cell) were generated separately for each APOE group and each cell type. To emphasize highly predictive features, all bar plots display the square root of the mean absolute SHAP value across splits. This SHAP analysis extends beyond classical feature importance analysis by conditioning on APOE genotype and leveraging the full multimodal model rather than gene expression alone. It reveals genotype-specific predictive features that may not appear when aggregating across the full dataset, thus providing a mechanistically interpretable and genetically stratified view of the transcriptional programs driving AD predictions across distinct brain cell types.

### Differential expression analysis

We performed cell-type–specific differential expression (DE) analyses to identify genes associated with Alzheimer’s disease (AD) across major brain cell types, including microglia, astrocytes, inhibitory neurons, excitatory neurons, oligodendrocytes, and oligodendrocyte precursor cells. For each analysis, cells were subset using broad cell-type annotations, and raw UMI count matrices were used directly. Genes were filtered prior to modeling to remove features with no variability or minimal expression. Specifically, genes with zero variance across cells were excluded, followed by expression-based filtering. In the Mathys et al. 2019 cohort (48 donors), genes were retained if they exceeded 1 count per million (CPM) in at least 10 cells. In the expanded Mathys et al. 2023 cohort (427 donors), genes were retained if detected in more than 20% of cells within a given cell type. Aside from this gene-filtering step, identical modeling procedures were used across cohorts.

To account for the hierarchical structure of single-nucleus RNA-seq data, with many cells nested within donors, we modeled gene expression using donor-aware generalized linear mixed-effects models. For each gene, we fitted a Poisson mixed-effects model with a log link, including a donor-specific random intercept to account for inter-individual variability. Alzheimer’s disease status was included as the primary fixed effect of interest, and a log-transformed library size offset was used to account for differences in sequencing depth across cells. To further control for unobserved technical and biological confounders, we incorporated residual-based unwanted variation factors derived using RUVSeq, following best practices for snRNA-seq analysis. RUV covariates capture global donor-level unwanted variation, while the donor random intercept accounts for residual within-donor correlation across cells; these components address complementary sources of confounding. Briefly, raw counts were first aggregated at the donor level within each cell type, and an initial donor-level generalized linear model was fitted using edgeR. Deviance residuals from this model were then used to estimate latent unwanted variation factors (RUVr), which were subsequently merged back into the cell-level metadata and included as covariates in the mixed-effects model. We retained the top 10 RUV components for all analyses, consistent with prior snRNA-seq studies and to ensure reproducibility and comparability across cell types and cohorts, and included them as fixed-effect covariates in the mixed-effects models. This procedure allows donor-level technical variation to be controlled without conditioning on disease status at the cell level.

Formally, for each gene, we fit the model given in Eq. (1):

1 $${counts}{{\_}}{ij} \sim {Poisson}(\mu {\_}{ij}),log (\mu {\_}{ij})=\beta {\_}0+\beta {\_}1 \; {AD}{\_}i+\varSigma {\_}k\gamma {\_}{k \, W}{\_}{ik} \\+\log ({libsize}{\_}{ij})+b{\_}d(i)$$

where i indexes cells, j indexes genes, W_*ik* denotes RUV covariates, and b_*d(i)* is a donor-specific random intercept. Counts were assumed to follow a Poisson distribution conditional on the model covariates.

Models were fit using the glmer function from the **lme4** package with adaptive Gauss–Hermite quadrature (nAGQ = 10) and the *bobyqa* optimizer. Estimated coefficients for AD status were extracted for each gene, and *p*-values were adjusted for multiple testing using the Benjamini–Hochberg procedure. Genes were considered differentially expressed if the false discovery rate (FDR) was less than 0.05 and the absolute log2 fold change exceeded 0.25.

As a robustness analysis, we also fit fixed-effects models adjusting for age at death, sex, and post-mortem interval (PMI), and observed strong concordance in estimated log2 fold changes between fixed-effects and mixed-effects specifications. We additionally fit an alternative fixed-effects model that included age at death, sex, post-mortem interval (PMI), APOE genotype and sequencing batch as covariates, yielding similarly high concordance of log₂ fold-change estimates across all cell types, further supporting the stability of inferred transcriptional effect sizes to alternative confounder adjustments. All results reported in the main text are derived from the donor-aware mixed-effects models. Differential expression analyses were performed separately for clinical diagnosis (dcfdx_lv) and neuropathological burden (CERAD score).

For each cell type, Spearman correlation of log2 fold-change values was computed across genes shared between models, assessing robustness of effect-size estimates to model specification. All results reported in the main text are derived from the donor-aware mixed-effects models. Differential expression analyses were performed separately for clinical diagnosis (dcfdx_lv) and neuropathological burden (CERAD score). All differential expression analyses were performed in R v4.4.2 using the lme4 (v1.1.38), glmmTMB (v1.1.13), RUVSeq (v1.40.0) and dplyr (v1.1.4) packages.

### GWAS enrichment

To contextualize cell-type-specific transcriptional predictors within the known genetic risk architecture of Alzheimer’s disease (AD), we systematically evaluated their overlap with genome-wide association study (GWAS) loci. Significant AD-associated single-nucleotide polymorphisms (SNPs) were curated from the NHGRI-EBI GWAS Catalog (accessed via the term MONDO_0004975, “Alzheimer’s disease”). SNPs were mapped to associated genes using the mappedGenes field in the catalog. For each major brain cell type, we compiled lists of predictive genes identified through our supervised machine learning classifiers. To ensure robustness, genes were retained only if they exhibited non-zero feature importance in at least two out of five cross-validation splits. Gene symbols were standardized to uppercase to ensure consistency in matching. As a background set for these enrichment analyses, we used the full set of 17,926 genes expressed in our single-nucleus RNA-seq dataset (Synapse accession: syn18485175), derived from the normalized expression matrix. This strategy controls for expression-dependent biases and provides a consistent denominator across analyses. To assess enrichment of GWAS signals, we performed two-sided Fisher’s exact tests comparing predictive genes with GWAS-mapped genes, relative to the background gene set, for each cell type. Overlapping genes, odds ratios, and *p* values were recorded in Supplementary Data. The full analysis was repeated analogously using predictors from classifiers trained to predict neuritic plaque burden using CERAD scores (pathology-based AD model). As with the clinical AD model, CERAD predictors were retained if non-zero feature importance was observed in at least two of five splits.

### GWAS–eQTL colocalization analysis

To assess whether cell-type–specific predictive genes were supported by shared genetic regulation between Alzheimer’s disease (AD) risk loci and brain gene expression, we performed Bayesian colocalization analysis between AD GWAS summary statistics and brain eQTLs using the coloc R package (v5.2.3, R v4.5.1). We used genome-wide association summary statistics from a large-scale AD GWAS (GCST90027158), aligned to the GRCh38 reference genome. GWAS variants were filtered to autosomal chromosomes (1–22) and harmonized to ensure consistent allele representation. When odds ratios were provided, effect sizes were converted to log-odds (β = log(OR)). Variants were deduplicated by genomic position (chr:pos), retaining the most significant association per position to avoid duplicated SNPs. Total sample size and case proportion were specified explicitly (111,326 cases; 677,663 controls), and the GWAS was modeled as a case–control trait within the colocalization framework.

Cis-eQTL summary statistics were obtained from GTEx v10 for all available brain tissues. For each tissue, significant variant–gene pairs were loaded from preprocessed eQTL files. Ensembl gene identifiers were harmonized by removing version suffixes, and variant identifiers were parsed to extract chromosome, position, reference allele, and alternate allele. For each tissue, the effective sample size was approximated using the maximum reported number of samples contributing to eQTL estimation. This approximation was used because per-variant effective sample sizes are not uniformly available across GTEx eQTLs and provides a consistent tissue-level estimate for colocalization.

Colocalization was restricted to genes we identified as predictive via the supervised learning models. For each major brain cell type (Astrocytes, Microglia, Inhibitory neurons, Excitatory neurons, Oligodendrocytes, and Oligodendrocyte progenitor cells), predictive genes were defined as those with non-zero feature importance in at least two of five donor-aware cross-validation splits. Gene symbols were standardized to uppercase and mapped to Ensembl gene identifiers using BioMart (GRCh38), retaining only genes located on autosomes or sex chromosomes. Genomic coordinates (chromosome, transcription start, and end positions) were used to define gene-centered regions for colocalization testing.

For each predictive gene, colocalization was performed independently for every brain tissue. A symmetric genomic window extending ±250 kb from the gene boundaries was defined. Within each region, GWAS and eQTL datasets were subset to variants falling inside the window. Regions were tested only if both datasets contained at least 10 variants and shared at least 10 overlapping SNPs after harmonization by genomic position. Bayesian colocalization was performed using approximate Bayes factors as implemented in the coloc.abf function. For coloc.abf we used the default prior probabilities (p1 = 1 × 10⁻⁴, p2 = 1 × 10⁻⁴, p12 = 1 × 10⁻⁵). GWAS data were modeled as a case–control trait, and eQTL data were modeled as a quantitative trait. For each gene–cell type–tissue combination, posterior probabilities were computed for the five standard hypotheses: no association (H0), GWAS-only association (H1), eQTL-only association (H2), two distinct causal variants (H3), or a shared causal variant (H4). The number of SNPs contributing to each test was recorded.

Colocalization results were aggregated across all tested genes, cell types, and brain tissues. To summarize evidence for shared genetic regulation, posterior probability for hypothesis H4 (PP.H4) was used as the primary metric. Results were filtered using predefined thresholds (PP.H4 ≥ 0.5 for moderate evidence and PP.H4 ≥ 0.8 for strong evidence of colocalization). For downstream summaries, genes were considered “anchored” if they (i) were predictive in a given cell type, (ii) were tested in colocalization analysis, and (iii) exhibited PP.H4 ≥ 0.8 in at least one brain tissue. All filtering and aggregation steps were applied using fixed, pre-specified criteria and were not adjusted based on downstream predictive performance, effect size, or prior biological knowledge.

### Pseudotime inference and trajectory analysis

To characterize continuous disease-associated transcriptional trajectories within defined cell subclusters, we performed pseudotime analysis using a non-circular, unsupervised feature selection strategy anchored to control-like cellular states. All analyses were conducted independently within each annotated subcluster to avoid confounding by broad cell-type differences. Among the 41 subclusters identified by Mathys et al., we analyzed those containing at least 50 cells and representation from both Alzheimer’s disease (AD) and control individuals. For each eligible subcluster, we constructed a subcluster-specific expression matrix from the normalized single-nucleus RNA-seq count matrix. Expression values were library-size normalized to a target sum of 10,000 counts per cell and log-transformed prior to downstream analysis.

To avoid circularity between trajectory inference and disease association, we selected features for pseudotime construction using highly variable genes (HVGs) within each subcluster, rather than differentially expressed genes or model-derived predictors. Specifically, within each subcluster, we identified the top 2000 HVGs using the Seurat variance-stabilization method as implemented in Scanpy, or all genes if fewer than 2000 were available. Only HVGs were used for dimensionality reduction and trajectory inference. Principal component analysis (PCA) was performed on the HVG-restricted expression matrix, retaining up to 30 principal components (or fewer if limited by gene number). A k-nearest neighbor graph was constructed using these components (k = 10), followed by diffusion map embedding to capture continuous cellular transitions.

Diffusion pseudotime (DPT) was used to infer continuous trajectories within each subcluster. To orient trajectories in a biologically interpretable manner while avoiding disease-label leakage, root cells were selected based on neuropathological and cognitive severity measures rather than transcriptomic similarity or classifier outputs. For each subcluster and each random seed, candidate root cells were restricted to those with available CERAD neuritic plaque scores and cognitive diagnosis values. Among these, the root was chosen as a cell with the maximal CERAD score (least pathological burden) and minimal cognitive impairment score, corresponding to the most control-like pathological state. When multiple cells satisfied these criteria, one was selected at random using a fixed seed. This procedure ensured consistent anchoring of pseudotime to a putative healthy baseline while allowing robustness assessment. Diffusion pseudotime was computed separately for each seed using the selected root cell.

To assess the stability of inferred trajectories, pseudotime inference was repeated across multiple random seeds per subcluster. For each seed, diffusion pseudotime values were computed for all cells, and the association between pseudotime and disease status was evaluated using receiver operating characteristic (ROC) analysis and logistic regression. To aggregate pseudotime estimates across seeds while preserving relative ordering, pseudotime values were rank-normalized within each seed and averaged across seeds to obtain a mean pseudotime score per cell. This aggregated pseudotime was used for all downstream analyses, including visualization and gene association testing.

To identify genes most strongly associated with progression along the inferred trajectory, we examined the relationship between aggregated pseudotime and principal component structure. For each subcluster, we identified the principal component whose scores exhibited the strongest Spearman correlation (by absolute value) with mean pseudotime. Gene loadings from this component were interpreted as trajectory-influencing weights. Genes were ranked by the absolute value of their loadings on the selected component. For each subcluster, we defined the top 20 genes by absolute loading as trajectory-influencing genes, corresponding approximately to the top 1% of genes contributing to the pseudotime-aligned transcriptional axis. These genes were recorded for downstream aggregation and validation analyses.

To identify target genes repeatedly implicated by trajectory analysis, we quantified the frequency with which each gene appeared among the top trajectory-influencing genes across all subclusters. These analyses were performed strictly as post hoc summaries of trajectory structure and did not influence pseudotime construction, feature selection, or thresholding. The relationship between pseudotime and disease status was quantified using logistic regression models and ROC analysis. For clinical diagnosis, AD status was defined based on dfcdx_lv, while a separate analysis used CERAD plaque burden to define neuropathology-based disease status. Models were fit independently for each seed and aggregated across seeds using Fisher’s method for *p* value combination and averaging of log odds ratios for effect sizes. Trajectory inference and gene ranking were performed without reference to disease labels or model-derived predictors.

### Predictive validation cohort

We validated our gene expression–based classifiers using an independent single-nucleus RNA-seq dataset derived from Lau et al. ^13^. This dataset comprises gene-by-cell count matrices and accompanying metadata from 12 Alzheimer’s disease patients and 9 non-AD controls, generated from post-mortem prefrontal cortex tissue by droplet-based single-nucleus RNA-seq as described by Lau et al., from which we analyzed Astrocytes, Excitatory Neurons, Inhibitory Neurons, Microglia, and Oligodendrocytes. Briefly, Lau et al. isolated nuclei from frozen post-mortem prefrontal cortex of donors with AD (Braak stage ≥4) and non-demented controls (Braak stage ≤2) obtained from the South West Dementia Brain Bank, using Dounce homogenization followed by OptiPrep density-gradient separation; libraries were prepared with the Chromium Single Cell 3’ Library Kit v3 (10x Genomics), sequenced paired-end on a NovaSeq 6000, and aligned to a GRCh38 pre-mRNA reference using Cell Ranger v3.0.1. We downloaded the processed expression matrix and metadata. We performed independent cell-type annotation (see Cell type annotations across datasets) to ensure consistency with our primary cohort, and separately assigned labels using the marker genes published by Lau et al.; the two approaches agreed for 155,278 of 172,514 nuclei (90.0%). For our validation models, we analyzed a final set of 17,110 overlapping genes shared with our primary dataset. We first used the incremental classifiers fit on the Mathys et al. dataset, froze these classifiers, and performed one-shot classification on the Lau et al. dataset to examine predictive generalizability of the 1) Genes expression only classifier and 2) APOE Genotype + Gene expression classifier. For each cell type, classifiers trained on the full feature set and on top-k feature subsets (1–25) were evaluated on the Lau dataset, and the top classifier was reported.

We then replicated our full predictive modeling workflow, including feature selection, cross-validation, and classifier training, across the 5 major brain cell types to independently assess feature stability. For each cell type, genes were ranked by mean normalized feature importance in each cohort, and Spearman rank correlations were computed between cohorts. In addition, we quantified predictor-set overlap by defining “stable predictors” as genes exhibiting nonzero importance in at least two of five splits. Overlap was summarized using recall of the smaller predictor set, providing a conservative estimate of shared predictive signal independent of absolute feature-set size.

### Internal validation using spearman correlation analysis

To assess the relationship between TriSCOPE predictor importance and differential expression (DE) signals, we performed rank-based concordance analyses tailored to the data-generating context of each comparison. For the Mathys et al. 2019 dataset, from which TriSCOPE predictors were derived, we evaluated genome-wide concordance between predictor importance and DE significance by computing Spearman rank correlations between predictor rank and FDR-adjusted DE *p* value rank within each cell type. DE genes were ranked by adjusted *p* value (most significant first), and predictor ranks were defined by mean normalized feature importance across five independent train–test splits.

For datasets on which predictive modeling was not run, such as ROSMAP-48b, as well as additional published single-nucleus RNA-seq studies, we instead assessed concordance using effect-size–based rankings. Specifically, we computed Spearman correlations between predictor rank and absolute log2 fold-change rank for genes reported in each external DE analysis, again stratified by cell type.

### External validation using independent differential expression studies

To evaluate biological reproducibility beyond the ROSMAP cohort, we assessed concordance between TriSCOPE predictors and differential expression results reported in four independent single-cell Alzheimer’s disease studies (Morabito et al. ^63^, Sadick et al. ^64^, Su et al. ^65^, and Grubman et al. ^66^), collectively comprising 94 donors. These studies span distinct brain regions, sequencing chemistries, and preprocessing pipelines.

For each external dataset and matched cell type, genes were ranked by absolute log2 fold change, and compared to ranks of stable TriSCOPE predictors derived from the ROSMAP cohort. Spearman rank correlation was computed between external DEG ranks (as determined by absolute log fold change) and predictor ranks, and results were summarized both at the dataset–cell-type level and by averaging correlations across datasets within each cell type.

### Quality-control validation on Murphy et al. Dataset

We also performed a reanalysis of Mathys et al.’s work based on the cell types as defined by Murphy et al. Specifically, we used a dataset of 50,381 “high-quality” nuclei from all 6 major cell types collected using the filtering procedure described in Murphy et al. ^45^ Briefly, Murphy et al. reprocessed the Mathys et al. data with scFlow^67^, applying more stringent per-nucleus quality control and cell-type annotation. During predictive modeling we dropped 941 nuclei that were missing post-mortem interval values. We found our results to be robust to this higher standard quality-control method, further validating our findings. Furthermore, we ensured that during our differential expression analysis we performed our analysis using Poisson GLMMs, which have been prescribed as best practice and shown to be equivalent to pseudobulk DE methods for datasets with more than 500 cells^7,12,45^.

### Computational resources

All analyses were run on the Harvard Medical School O2 high-performance computing cluster under the Slurm workload manager, with jobs submitted separately per cell type and per train-test split. Runtime and peak memory usage for each analytical component are reported in Supplementary Data 22.

### Reporting summary

Further information on research design is available in the Nature Portfolio Reporting Summary linked to this article.

## Supplementary information

Supplementary Information

Description of Additional Supplementary Files

Supplementary Data 1

Supplementary Data 2

Supplementary Data 3

Supplementary Data 4

Supplementary Data 5

Supplementary Data 6

Supplementary Data 7

Supplementary Data 8

Supplementary Data 9

Supplementary Data 10

Supplementary Data 11

Supplementary Data 12

Supplementary Data 13

Supplementary Data 14

Supplementary Data 15

Supplementary Data 16

Supplementary Data 17

Supplementary Data 18

Supplementary Data 19

Supplementary Data 20

Supplementary Data 21

Supplementary Data 22

Supplementary Data 23

Supplementary Data 24

Reporting Summary

Transparent Peer Review file

## Source data

Source Data

## Data Availability

The data used in this study are available as follows. 1) The Mathys-48 dataset is available on Synapse under accession syn18485175. ROSMAP metadata is also accessible via Synapse under accession syn3157322. This same snRNA-seq dataset with further quality control measures applied to it is publicly available on Synapse under accession number syn51758062.1. 2) The Lau-21 dataset is deposited on the NCBI Gene Expression Omnibus under accession GSE157827. 3) The ROSMAP-48b dataset was generated as part of the MIT ROSMAP Single-Nucleus Multiomics Study, and is available through Synapse under accession syn52293442. 4) The ROSMAP-427 dataset is available through Synapse under accession syn52293417. 5) Single-nucleus RNA-seq data from the Seattle Alzheimer’s Disease Brain Cell Atlas (SEA-AD) are available through the Allen Institute Open Data Registry on AWS and through controlled access on the AD Knowledge Portal on Synapse under accession syn26223298. Donor, library, and cell-level metadata are available on the Allen Institute Brain-Map portal (https://portal.brain-map.org/explore/seattle-alzheimers-disease). 6) Cis-eQTL data: GTEx v10 significant cis-eQTL variant–gene pairs for all brain tissues were obtained from the GTEx Portal (https://gtexportal.org/home/) on 07/26/25. 7) Alzheimer’s disease GWAS summary statistics: GWAS summary statistics were obtained from the NHGRI-EBI GWAS Catalog (https://www.ebi.ac.uk/gwas/efotraits/MONDO_0004975). 8) Processed differential expression results were obtained from four previously published studies: Morabito et al. ^63^ Supplementary Data 1C (https://doi.org/10.1038/s41588-021-00894-z), Sadick et al. ^64^ Tables S4 and S6 (https://doi.org/10.1016/j.neuron.2022.03.008), Su et al. ^65^ Table S5A (https://doi.org/10.1016/j.stem.2022.09.010), and Grubman et al. ^66^ Supplementary Data 2 (https://doi.org/10.1038/s41593-019-0539-4). Each file is available from the supplementary information of the corresponding publication. Access to the ROSMAP-derived and SEA-AD datasets is controlled to protect participant privacy, as these are classified as sensitive NIH controlled-access data. Access requires an executed Data Use Agreement and registration with the data provider; conditions of access, required approvals and expected response times are maintained by the providers at https://adknowledgeportal.synapse.org and https://www.radc.rush.edu/docs/omics/overview.htm. We did not generate these data and had no role in setting the conditions of access. Source data are provided with this paper.

## Code availability

All code used in this study is available in a public GitHub repository (https://github.com/AdiVM/TriSCOPE_RNASeq_Alzheimers_Analysis). The version of the code used in this study is archived on Zenodo^68^ and permanently citable at (https://doi.org/10.5281/zenodo.21793738). A synthetic demonstration dataset for testing the software is included in the repository (triscope_synthetic_demo.zip). The repository is released under the MIT License, an Open Source Initiative–approved licence. No restrictions apply to access or reuse of the code.
