# Supplementary material for: Single-cell machine learning uncovers genetically anchored, cell-type specific programs of Alzheimer’s disease
Source: Nat Commun. 2026 Nov 9;17:11124. doi: 10.1038/s41467-026-77651-5 (PMC13748584; doi:10.1038/s41467-026-77651-5)
Supplement: Supplementary file 1 [file 41467_2026_77651_MOESM1_ESM.pdf]

Supplementary Materials

Madduri et al., Single-cell machine learning uncovers genetically anchored, cell-type specific programs of Alzheimer’s disease

Supplementary Figures

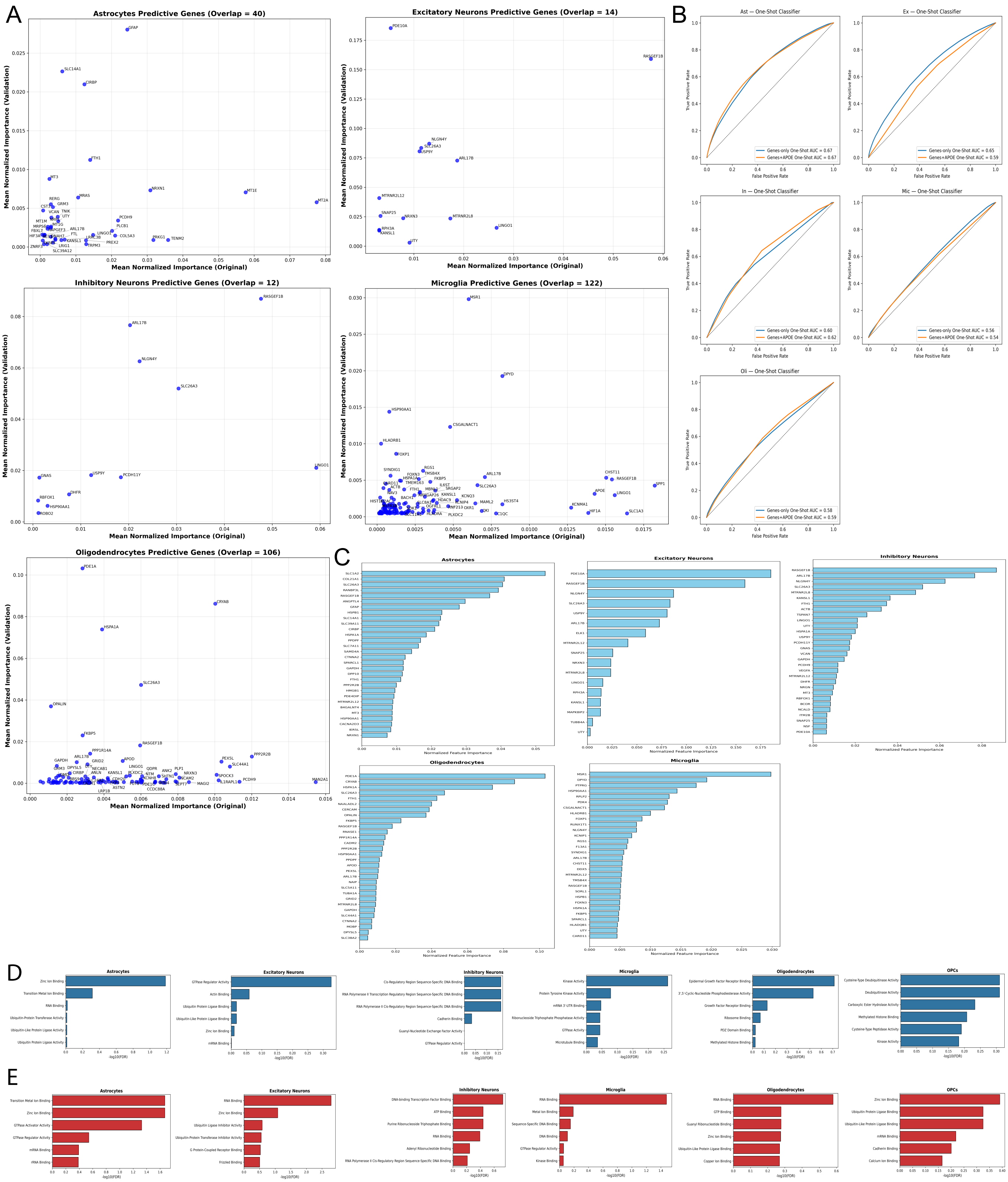

F

Method	Input	Output	Primary Question Answered	Typical Use Case
**Predictive Modeling**	Expression + covariates (supervised, joint)	Conditionally informative genes	Which genes still matter when modeled jointly?	Feature prioritization, robustness across donors
**Differential Expression**	Expression + covariates (supervised, marginal)	Marginal gene effects	Which genes differ on average?	Atlas construction, pathway discovery
**Pseudotime Inference**	Expression (unsupervised)	Trajectory-aligned genes	Which genes change monotonically along disease progression?	Temporal contextualization, progression consistency

Supplementary Figure 1: Supporting analyses for TriSCOPE modeling via cross-cohort validation, pathway enrichment, and framework comparison

a, Scatterplots comparing normalized predictive feature importance for genes independently identified in both the Mathys-48 and Lau-21 datasets across five major brain cell types (Astrocytes, Excitatory Neurons, Inhibitory Neurons, Microglia, Oligodendrocytes). Genes are plotted by mean normalized importance in Mathys-48 (x-axis) versus Lau-21 (y-axis), with overlap counts indicated in each panel. **b,** Receiver operating characteristic (ROC) curves showing one-shot classification performance in the independent Lau-21 dataset. Classifiers trained on Mathys-48 using either the full feature set or incremental subsets of the top k features (1–25) were frozen and applied directly to Lau-21 without retraining; for each cell type the best-performing model is shown. Results are shown for gene expression only (blue) or gene expression plus APOE genotype (orange). **c,** Bar plots of top-ranked predictive features for classifiers trained de novo on Lau-21 across the same five cell types. Normalized feature importance values converge with predictors identified in Mathys-48. **d,** Bar plots of top enriched GO molecular function terms for predictive genes in the Mathys-48 discovery cohort, defined as genes assigned predictive feature importance in two or more splits, shown separately for each of the six major brain cell types, adding Oligodendrocyte Progenitor Cells to those in a. **e,** Bar plots of top enriched GO molecular function terms for differentially expressed genes in the Mathys-48 discovery cohort, defined by donor-aware analysis with an FDR-adjusted $P < 0.05$, shown separately for the same six cell types. In **d** and **e,** enrichment was performed by preranked gene set enrichment analysis (GSEA; gseapy) against GO Molecular Function 2023, and $-\log_{10}$(FDR) is shown on the x-axis. **f,** Summary table illustrating the complementary analytical roles integrated by TriSCOPE: predictive modeling identifies genes that remain informative when modeled jointly with covariates, differential expression captures average marginal differences, and pseudotime inference highlights genes aligned with transcriptional progression. Analyses in **a**–**c** comprise n = 70,634 nuclei from n = 48 donors (Mathys-48) and n = 172,514 nuclei from n = 21 donors (Lau-21); **d** and **e** are derived from Mathys-48. Source data are provided as a Source Data file.

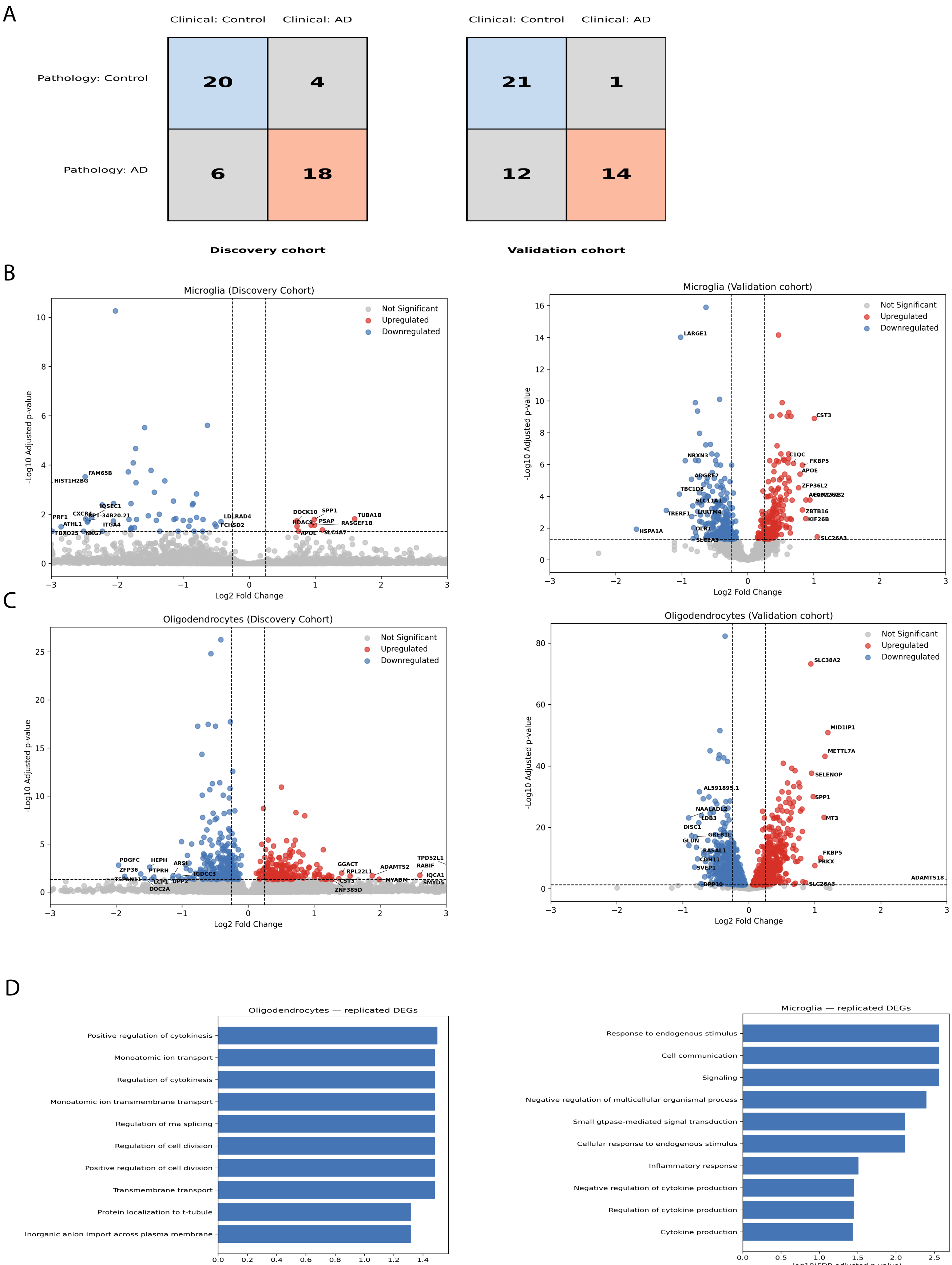
A
Clinical: Control
Clinical: AD
Pathology: Control
Pathology: AD
20
4
6
18
Discovery cohort
Clinical: Control
Clinical: AD
21
1
12
14
Validation cohort
B
Microglia (Discovery Cohort)
Not Significant
Upregulated
Downregulated
-Log10 Adjusted p-value
Log2 Fold Change
FAM65B
HIST1H2BG
IQSEC1
PRF1
CXCR4
RP1-34B20.21
ATHL1
ITGA4
FBXO25
NKG7
LDLRAD4
FCHSD2
DOCK10
SPP1
TUBA1B
HDAC9
PSAP
RASGEF1B
APOE
SLC4A7
Microglia (Validation cohort)
LARGE1
CST3
NRXN3
C1QC
FKBP5
APOE
ADGRE2
TBC1D8
ZFP36L2
SLC11A1
TRERF1
LRRTM4
ZBTB16
KIF26B
HSPA1A
OLR1
SLC2A3
SLC26A3
C
Oligodendrocytes (Discovery Cohort)
PDGFC
HEPH
ZFP36
PTPRH
ARSI
TSPAN11
LCP1
UPP2
IGDCC3
DOC2A
GGACT
RPL22L1
ADAMTS2
MYADM
CST3
ZNF385D
TPD52L1
RABIF
IQCA1
SMYD3
Oligodendrocytes (Validation cohort)
SLC38A2
MID1IP1
METTL7A
SELENOP
AL591895.1
SPP1
NAALADL2
LDB3
MT3
DISC1
GREB1L
GLDN
RASAL1
CDH11
FKBP5
PRKX
SVEP1
ADAMTS18
DPP10
SLC26A3
D
Oligodendrocytes — replicated DEGs
Positive regulation of cytokinesis
Monoatomic ion transport
Regulation of cytokinesis
Monoatomic ion transmembrane transport
Regulation of rna splicing
Regulation of cell division
Positive regulation of cell division
Transmembrane transport
Protein localization to t-tubule
Inorganic anion import across plasma membrane
-log10(FDR-adjusted p-value)
Microglia — replicated DEGs
Response to endogenous stimulus
Cell communication
Signaling
Negative regulation of multicellular organismal process
Small gtpase-mediated signal transduction
Cellular response to endogenous stimulus
Inflammatory response
Negative regulation of cytokine production
Regulation of cytokine production
Cytokine production
-log10(FDR-adjusted p-value)

Supplementary Figure 2: Differential expression analysis across combined clinical–pathological groups.

a, Distribution of donors across clinical and neuropathological categories in the Mathys-48 and ROSMAP-48b datasets, showing separation into four combined groups (Clinical Control/Pathology Control, Clinical AD/Pathology Control, Clinical Control/Pathology AD, and Clinical AD/Pathology AD). n = 24 neuropathology-positive donors in Mathys-48 (18 clinical AD, 6 without) and n = 26 in ROSMAP-48b (14 clinical AD, 12 without). **b,** Volcano plots showing random-effects differential expression in microglia comparing clinically diagnosed Alzheimer's disease cases and resilient individuals matched for neuropathological burden. Genes consistently differentially expressed across discovery and validation cohorts highlight microglial transcriptional programs associated with clinical manifestation independent of pathology severity. **c,** Volcano plots showing random-effects differential expression analysis in oligodendrocytes comparing clinically symptomatic and resilient individuals under matched neuropathology across discovery and validation cohorts. Oligodendrocytes exhibit a broader and more reproducible set of differentially expressed genes, suggesting widespread transcriptional remodeling associated with clinical outcome. In **b** and **c**, differential expression was assessed using a donor-aware Poisson generalized linear mixed model with a log link, an offset for library size, RUV covariates as fixed effects, and a random intercept for donor; *P* values are from two-sided Wald tests and were adjusted using the Benjamini–Hochberg procedure. Points are coloured by significance (upregulated, red; downregulated, blue; not significant, grey) at FDR-adjusted $P < 0.05$; dashed lines indicate $|\log_2$ fold change$| = 0.25$ and FDR-adjusted $P = 0.05$. **d,** Pathway enrichment analysis of genes showing consistent random-effects differential expression across cohorts in microglia and oligodendrocytes. Enriched pathways reflect inflammatory signaling in microglia and stress, transport, and cell-cycle–related processes in oligodendrocytes, highlighting cell-type–specific programs associated with divergent clinical outcomes under similar neuropathological burden. Enrichment was performed using g:Profiler (organism *Homo sapiens*; GO:BP, Reactome and KEGG source databases) with a significance threshold of 0.05; $-\log_{10}$(FDR-adjusted *P*) is shown on the x-axis. Source data are provided as a Source Data file.

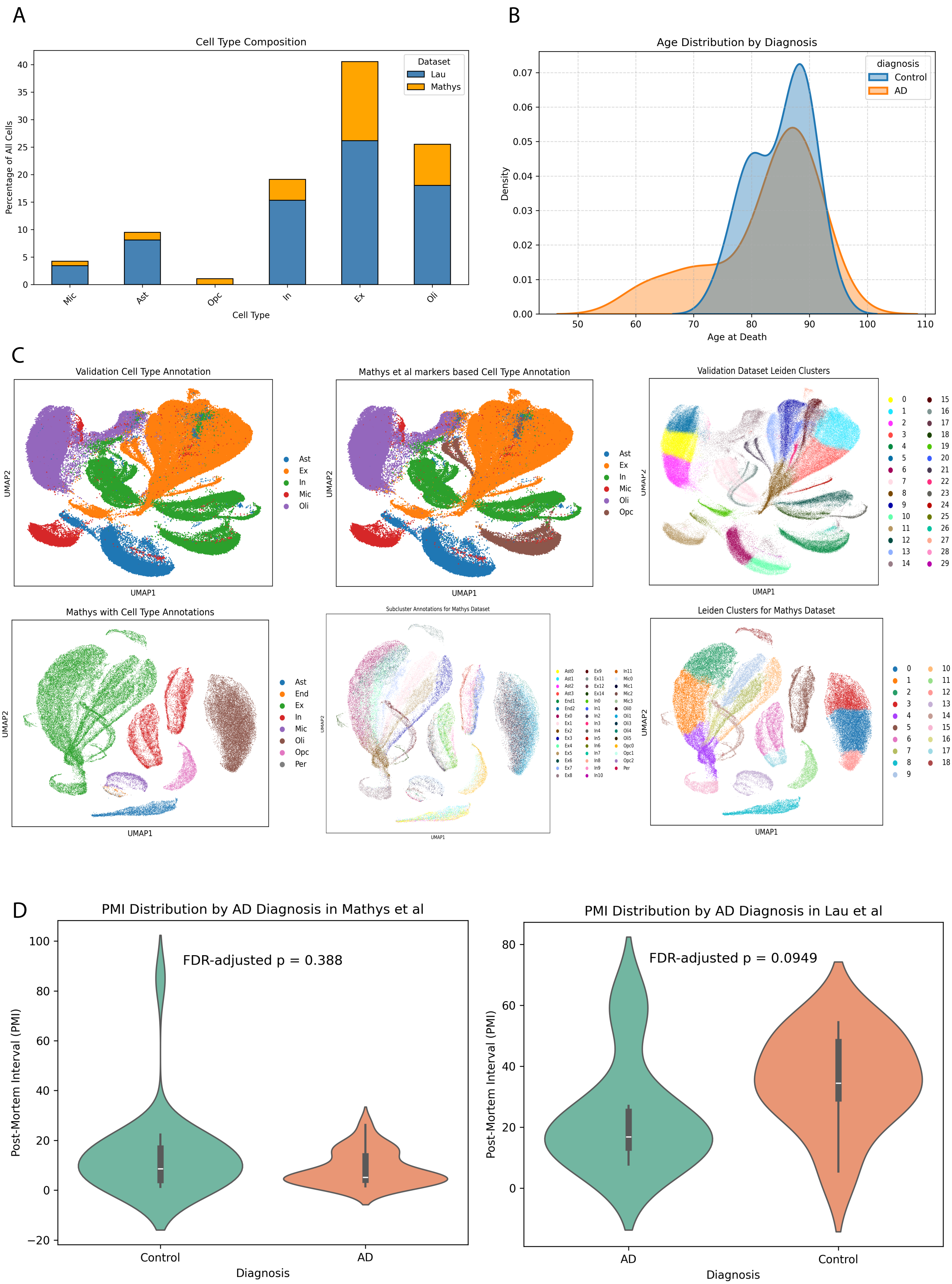

A
Cell Type Composition
Percentage of All Cells
Cell Type
Dataset
Lau
Mathys
Mic
Ast
Opc
In
Ex
Oli
B
Age Distribution by Diagnosis
Density
Age at Death
diagnosis
Control
AD
C
Validation Cell Type Annotation
Mathys et al markers based Cell Type Annotation
Validation Dataset Leiden Clusters
Mathys with Cell Type Annotations
Subcluster Annotations for Mathys Dataset
Leiden Clusters for Mathys Dataset
UMAP1
UMAP2
D
PMI Distribution by AD Diagnosis in Mathys et al
FDR-adjusted p = 0.388
PMI Distribution by AD Diagnosis in Lau et al
FDR-adjusted p = 0.0949
Post-Mortem Interval (PMI)
Diagnosis
Control
AD

Supplementary Figure 3: Demographic, compositional, and clustering characteristics of Mathys-48 and Lau-21 datasets.

a, Cell type composition across both Mathys and Lau datasets, demonstrating broadly consistent representation of astrocytes, excitatory neurons, inhibitory neurons, microglia, and oligodendrocytes. **b,** Age-at-death distributions for AD cases and controls in Mathys-48 and Lau-21 cohorts, indicating similar demographic ranges. **c,** Six UMAP projections, arranged in two rows and read left to right. Top row: the Lau-21 dataset annotated using marker genes from the original study; the Lau-21 dataset annotated using Mathys-48 marker genes, demonstrating concordance in broad cell type assignment with the preceding panel; and Leiden clustering of the Lau-21 dataset, capturing additional substructure within annotated cell types. Bottom row: the Mathys-48 dataset annotated with broad cell types; subcluster-level annotation of the Mathys-48 dataset, showing finer stratification within major cell classes; and Leiden clustering of the Mathys-48 dataset, highlighting resolution of transcriptionally distinct subpopulations. Cell type and cluster identities are defined by the key adjacent to each panel. **d,** Post-mortem interval (PMI) distributions for clinical AD versus control donors in Mathys-48 (left) and Lau-21 (right). PMI was compared between groups using two-sided Mann–Whitney U tests with Benjamini–Hochberg correction; exact FDR-adjusted *P* values are shown on each panel. Violins show the full distribution, with the internal box indicating the median and interquartile range. Source data are provided as a Source Data file.

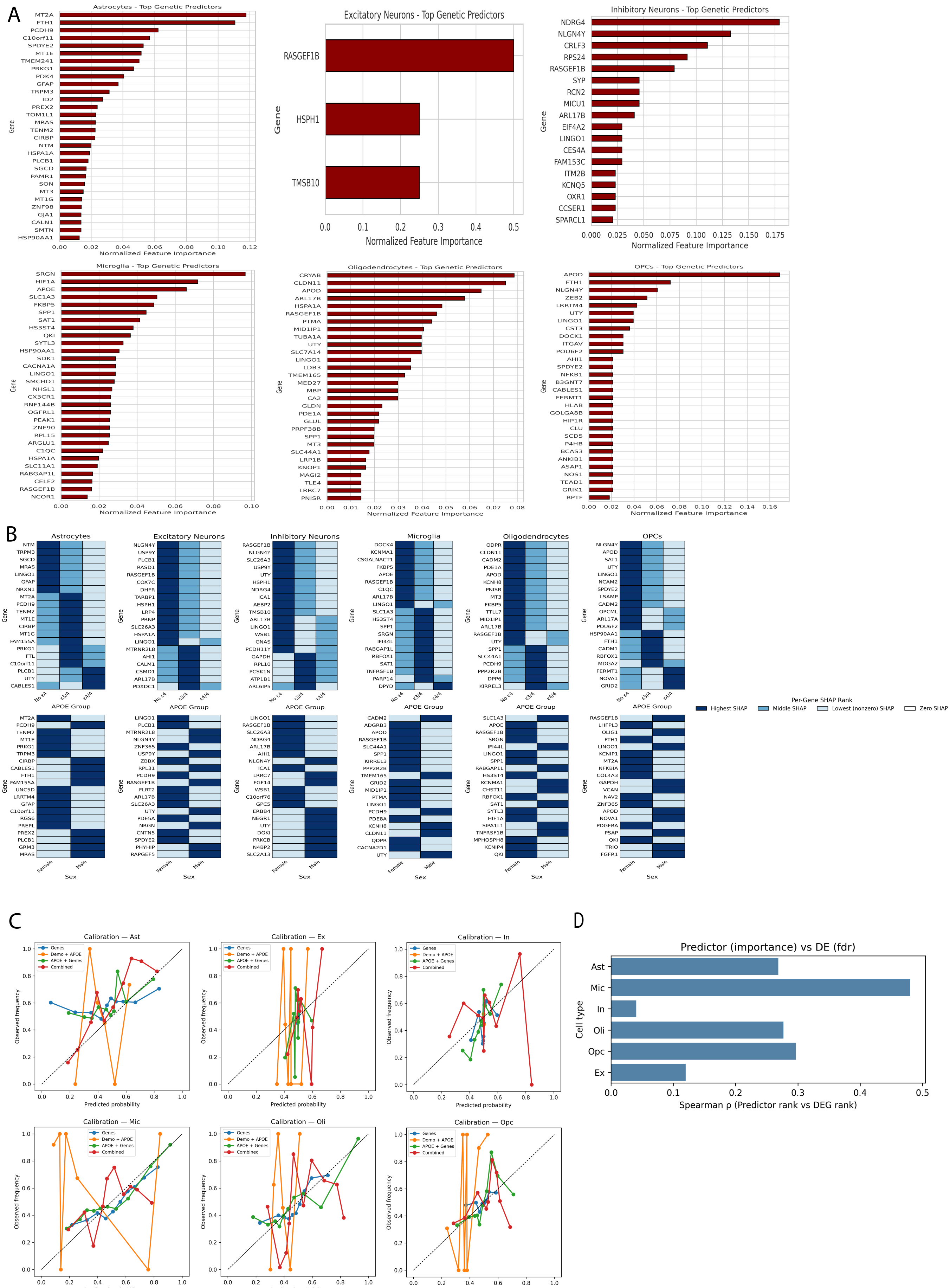

A
Astrocytes - Top Genetic Predictors
MT2A
FTH1
PCDH9
C10orf11
SPDYE2
MT1E
TMEM241
PRKG1
PDK4
GFAP
TRPM3
ID2
PREX2
TOM1L1
MRAS
TENM2
CIRBP
NTM
HSPA1A
PLCB1
SGCD
PAMR1
SON
MT3
MT1G
ZNF98
GJA1
CALN1
SMTN
HSP90AA1
Gene
Normalized Feature Importance
Excitatory Neurons - Top Genetic Predictors
RASGEF1B
HSPH1
TMSB10
Gene
Normalized Feature Importance
Inhibitory Neurons - Top Genetic Predictors
NDRG4
NLGN4Y
CRLF3
RPS24
RASGEF1B
SYP
RCN2
MICU1
ARL17B
EIF4A2
LINGO1
CES4A
FAM153C
ITM2B
KCNQ5
OXR1
CCSER1
SPARCL1
Gene
Normalized Feature Importance
Microglia - Top Genetic Predictors
SRGN
HIF1A
APOE
SLC1A3
FKBP5
SPP1
SAT1
HS3ST4
QKI
SYTL3
HSP90AA1
SDK1
CACNA1A
LINGO1
SMCHD1
NHSL1
CX3CR1
RNF144B
OGFRL1
PEAK1
ZNF90
RPL15
ARGLU1
C1QC
HSPA1A
SLC11A1
RABGAP1L
CELF2
RASGEF1B
NCOR1
Gene
Normalized Feature Importance
Oligodendrocytes - Top Genetic Predictors
CRYAB
CLDN11
APOD
ARL17B
HSPA1A
RASGEF1B
PTMA
MID1IP1
TUBA1A
UTY
SLC7A14
LINGO1
LDB3
TMEM165
MED27
MBP
CA2
GLDN
PDE1A
GLUL
PRPF38B
SPP1
MT3
SLC44A1
LRP1B
KNOP1
MAGI2
TLE4
LRRC7
PNISR
Gene
Normalized Feature Importance
OPCs - Top Genetic Predictors
APOD
FTH1
NLGN4Y
ZEB2
LRRTM4
UTY
LINGO1
CST3
DOCK1
ITGAV
POU6F2
AHI1
SPDYE2
NFKB1
B3GNT7
CABLES1
FERMT1
HLAB
GOLGA8B
HIP1R
CLU
SCD5
P4HB
BCAS3
ANKIB1
ASAP1
NOS1
TEAD1
GRIK1
BPTF
Gene
Normalized Feature Importance
B
Astrocytes
Excitatory Neurons
Inhibitory Neurons
Microglia
Oligodendrocytes
OPCs
Gene
No ε4
ε3/4
ε4/4
APOE Group
Per-Gene SHAP Rank
Highest SHAP
Middle SHAP
Lowest (nonzero) SHAP
Zero SHAP
Female
Male
Sex
C
Calibration — Ast
Calibration — Ex
Calibration — In
Calibration — Mic
Calibration — Oli
Calibration — Opc
Genes
Demo + APOE
APOE + Genes
Combined
Observed frequency
Predicted probability
D
Predictor (importance) vs DE (fdr)
Ast
Mic
In
Oli
Opc
Ex
Cell type
Spearman ρ (Predictor rank vs DEG rank)

Supplementary Figure 4: SHAP-based interpretation of predictive models for clinical AD status stratified by APOE genotype

a, Bar plots showing the top gene-level predictors of clinical AD classification across the six major brain cell types, identified by feature importance analysis for the Mathys-48 dataset. **b,** Heatmaps displaying per-gene SHAP ranks across APOE genotype groups (non-carriers, heterozygous ε4, homozygous ε4/ε4; upper row) and across sex (female, male; lower row), shown separately for each cell type. Shading indicates SHAP rank and is defined by the key within the image. **c,** Calibration curves for each cell type, plotting observed frequency against predicted probability for four model configurations (gene expression only; demographics plus APOE genotype; APOE genotype plus gene expression; and the combined model), identified by the key within each panel. The dashed diagonal indicates perfect calibration. **d,** Bar plot of the Spearman rank correlation (ρ) between predictor importance rank and differentially expressed gene rank for each cell type. The analyses reported here were performed on the Mathys-48 dataset (n = 70,634 nuclei from n = 48 donors). Source data are provided as a Source Data file.

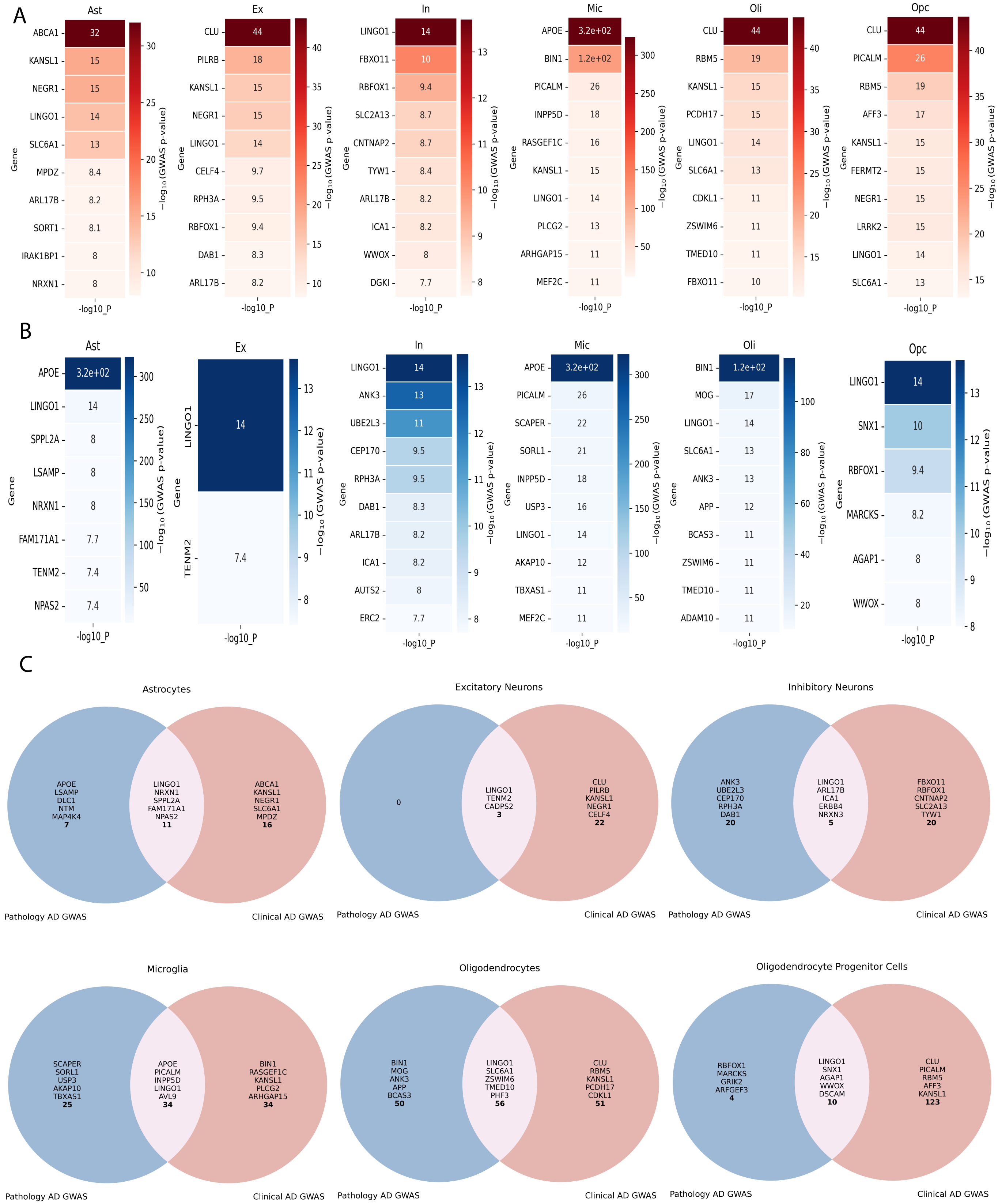

A
Ast
ABCA1 32
KANSL1 15
NEGR1 15
LINGO1 14
SLC6A1 13
MPDZ 8.4
ARL17B 8.2
SORT1 8.1
IRAK1BP1 8
NRXN1 8
Ex
CLU 44
PILRB 18
KANSL1 15
NEGR1 15
LINGO1 14
CELF4 9.7
RPH3A 9.5
RBFOX1 9.4
DAB1 8.3
ARL17B 8.2
In
LINGO1 14
FBXO11 10
RBFOX1 9.4
SLC2A13 8.7
CNTNAP2 8.7
TYW1 8.4
ARL17B 8.2
ICA1 8.2
WWOX 8
DGKI 7.7
Mic
APOE 3.2e+02
BIN1 1.2e+02
PICALM 26
INPP5D 18
RASGEF1C 16
KANSL1 15
LINGO1 14
PLCG2 13
ARHGAP15 11
MEF2C 11
Oli
CLU 44
RBM5 19
KANSL1 15
PCDH17 15
LINGO1 14
SLC6A1 13
CDKL1 11
ZSWIM6 11
TMED10 11
FBXO11 10
Opc
CLU 44
PICALM 26
RBM5 19
AFF3 17
KANSL1 15
FERMT2 15
NEGR1 15
LRRK2 15
LINGO1 14
SLC6A1 13
Gene
-log10_P
−log₁₀(GWAS p-value)
B
Ast
APOE 3.2e+02
LINGO1 14
SPPL2A 8
LSAMP 8
NRXN1 8
FAM171A1 7.7
TENM2 7.4
NPAS2 7.4
Ex
LINGO1 14
TENM2 7.4
In
LINGO1 14
ANK3 13
UBE2L3 11
CEP170 9.5
RPH3A 9.5
DAB1 8.3
ARL17B 8.2
ICA1 8.2
AUTS2 8
ERC2 7.7
Mic
APOE 3.2e+02
PICALM 26
SCAPER 22
SORL1 21
INPP5D 18
USP3 16
LINGO1 14
AKAP10 12
TBXAS1 11
MEF2C 11
Oli
BIN1 1.2e+02
MOG 17
LINGO1 14
SLC6A1 13
ANK3 13
APP 12
BCAS3 11
ZSWIM6 11
TMED10 11
ADAM10 11
Opc
LINGO1 14
SNX1 10
RBFOX1 9.4
MARCKS 8.2
AGAP1 8
WWOX 8
Gene
-log10_P
−log₁₀(GWAS p-value)
C
Astrocytes
APOE LSAMP DLC1 NTM MAP4K4 7
LINGO1 NRXN1 SPPL2A FAM171A1 NPAS2 11
ABCA1 KANSL1 NEGR1 SLC6A1 MPDZ 16
Excitatory Neurons
0
LINGO1 TENM2 CADPS2 3
CLU PILRB KANSL1 NEGR1 CELF4 22
Inhibitory Neurons
ANK3 UBE2L3 CEP170 RPH3A DAB1 20
LINGO1 ARL17B ICA1 ERBB4 NRXN3 5
FBXO11 RBFOX1 CNTNAP2 SLC2A13 TYW1 20
Microglia
SCAPER SORL1 USP3 AKAP10 TBXAS1 25
APOE PICALM INPP5D LINGO1 AVL9 34
BIN1 RASGEF1C KANSL1 PLCG2 ARHGAP15 34
Oligodendrocytes
BIN1 MOG ANK3 APP BCAS3 50
LINGO1 SLC6A1 ZSWIM6 TMED10 PHF3 56
CLU RBM5 KANSL1 PCDH17 CDKL1 51
Oligodendrocyte Progenitor Cells
RBFOX1 MARCKS GRIK2 ARFGEF3 4
LINGO1 SNX1 AGAP1 WWOX DSCAM 10
CLU PICALM RBM5 AFF3 KANSL1 123
Pathology AD GWAS
Clinical AD GWAS

Supplementary Figure 5: Cell-type–specific predictive features overlapping Alzheimer's disease GWAS loci.

a, Heatmaps of clinical AD predictors that overlap genome-wide significant AD GWAS loci, shown separately for each of the six major brain cell types. Up to the ten most significant genes are displayed per cell type, with shading and cell values indicating $-\log_{10}$ GWAS P value as defined by the colour bar adjacent to each panel. **b,** As in **a,** for AD neuropathology predictors. APOE shows the strongest GWAS association in both astrocytes and microglia. **c,** Venn diagrams illustrating the overlap between clinical AD GWAS-supported predictors and neuropathology GWAS-supported predictors for each cell type, highlighting convergent and distinct sets of genetically supported features; counts and the five genes with the smallest GWAS P values are shown in each region. Predictors were defined as genes with non-zero feature importance in at least two of five cross-validation splits, and GWAS-mapped genes were curated from the NHGRI-EBI GWAS Catalog. Analyses were performed on the Mathys-48 dataset (n = 70,634 nuclei from n = 48 donors). Source data are provided as a Source Data file.

Supplementary Results

Benchmarking of Model Architectures

At a cell-type level, the best-performing architecture varied. In Astrocytes, LASSO achieved the highest mean AUC (0.589 ± 0.055), followed closely by elastic net (0.575 ± 0.050) and GBM (0.568 ± 0.064). In Excitatory neurons: LASSO again performed best (0.607 ± 0.054), with elastic net yielding comparable performance (0.603 ± 0.054), while GBM achieved a lower mean AUC (0.530 ± 0.042). In Inhibitory neurons Random forest performed best (0.656 ± 0.043), followed by GBM (0.628 ± 0.074). In Oligodendrocytes: GBM achieved the highest performance (0.652 ± 0.096), outperforming random forest (0.619 ± 0.077) and linear models. Oligodendrocyte progenitor cells (OPCs): Random forest achieved the highest mean AUC (0.569 ± 0.065), with elastic net and LASSO performing similarly. Microglia: GBM yielded the strongest performance (0.653 ± 0.085), marginally exceeding LASSO (0.651 ± 0.066).

Overall, GBM achieved the highest mean AUC in two of six cell types (Oligodendrocytes and Microglia), while LASSO and random forest were optimal in other cell populations. Importantly, when GBM was not the top-performing model, the absolute performance gap between GBM and the best model was small (ΔAUC ranging from 0.021 to 0.077), indicating that no single architecture dramatically outperformed others. These results demonstrate that TriSCOPE's predictive signal is robust across multiple modeling paradigms and that downstream biological conclusions do not depend on a specific classifier. We therefore focus on GBM models in the main text, as they balance predictive performance, nonlinear modeling capacity, and stable feature-importance estimates suitable for interpretation and integration with differential expression and pseudotime analyses.

Clinical AD Prediction

Our AD classifiers revealed significant cell-level heterogeneity with Microglia (mean AUC: 0.653 ± 0.085), Inhibitory Neurons (mean AUC: 0.628 ± 0.074) and Oligodendrocytes (mean AUC: 0.652 ± 0.096) all demonstrating significant predictive performance using gene expression data alone relative to demographics (Supplementary Data 4, Fig. 2a). Classifiers trained on APOE genotype (without demographics) and gene expression data, exhibited significant predictive power in all cell types, and outperformed classifiers trained on Demographics and APOE genotype in Astrocytes, Oligodendrocytes, Inhibitory Neurons, and Microglia (Supplementary Data 4, Fig. 2a). To identify the molecular drivers underlying each model's performance, we next ranked feature importance scores across all cell types, both in our combined data modality models and gene expression-only models. Across our combined data modality models, age at death, education, APOE ε4/ε4 genotype, APOE ε4 homozygous genotype, sex, *ARL17B*, *HSPA1A*, *RASGEF1B*, and *LINGO1* were the most predictive features across cell types (Fig. 2c). These findings reinforce that while demographics contribute to disease prediction, they alone do not explain the transcriptional heterogeneity observed in AD on a cell-type basis.

Notably, several genes repeatedly ranked among the most important predictors across cell types, including *RASGEF1B*, *LINGO1*, and *ARL17B* (Fig. 2c, d). These genes remained top-ranked even after accounting for demographic and APOE genotype, suggesting that they reflect intrinsic molecular signals of disease. *RASGEF1B*, a member of the RAS signaling pathway, which has an established role in synaptic plasticity and neuronal dysfunction, emerged as a potential cross-cell-type effector of AD vulnerability [1,2]. *LINGO1* is a known regulator of axonal regeneration and myelination, and has been implicated in Alzheimer's

disease [3]. *ARL17B*, which encodes Rapidly Accelerated Fibrosarcoma (RAF) GTPases, likewise demonstrated pan-cell-type importance consistent with links between the GTP-binding protein complex and amyloid precursor protein (APP) to disease progression[4,5,6].

While some features were shared across cell types, others were highly specific. In Excitatory Neurons, we uniquely found that *MTRNR2L1* and *MTRNR2L8* were top predictors (Fig. 2b, Supplementary Data 1). *MTRNR2L1* (also known as HN1) encodes Humanin, a mitochondrial-derived peptide linked to Alzheimer's, Parkinson's, and other neurodegenerative disorders due to its neuroprotective effects, including inhibition of neuroinflammation [7].

In Inhibitory Neurons, we identified *AHI1* as a strong predictor. *AHi1* has been linked to impaired cognition and reduced synaptic plasticity in prenatally stressed offspring[8], and its importance here suggests a potential role in neuronal dysfunction in AD. *SLC26A3,* chloride/bicarbonate exchanger with emerging roles in neuronal physiology, also emerged as a top predictor in Inhibitory Neurons (Fig. 2b). Notably, we also observed an enrichment of Y chromosome–linked genes, including *USP9Y*, *NLGN4Y*, and *UTY*, suggesting a possible male-specific transcriptional program related to AD vulnerability in this population of neurons (Supplementary Data 1).

In Microglia, we identified a distinct set of top-ranked predictive genes, suggesting that this cell type may follow a distinct disease-induced transcriptional trajectory. The strongest predictor was *IFI44L*, a gene previously linked to interferon signaling [9] and immune activation in the brain (Fig. 2b, Supplementary Data 1). Other highly ranked features included *SRGN*, *HIF1A*, and *APOE*, [10]. *PLXDC2*, a gene associated with immune response in tumor microenvironments, also emerged as a strong predictor, highlighting a potential immune effector role in Microglial

responses to AD (Supplementary Fig. 4a, Supplementary Data 1). Notably, *PTEN*, a gene previously shown to bind *PLXDC2* in macrophages to promote inflammatory activation and prominent in GWAS, was also predictive. Another top-ranked feature was *HSPA1A*, a molecular chaperone that has been linked to protein quality control and the inhibition of amyloid-β and neurofibrillary tangle formation[10,11]. Collectively, these predictors support a Microglia-specific molecular signature enriched for immune and stress-response regulators (Fig. 2b, Supplementary Data 1).

In Oligodendrocytes, top predictive features included *CRYAB*, *CLDN11*, and *APOD* (Supplementary Data 1). *CRYAB* encodes a molecular chaperone linked to stabilization of intermediate GFAP filaments and more generally linked to prevention of protein aggregation [12,13] possibly representing a response to protein aggregation and misfolding events in Alzheimer's [14]. In OPCs, three top targets, *APOD, FTH1*, and *COL4A3* emerged (Fig. 2b; Supplementary Data 1). In Astrocytes, top predictive features included *ID2*, *MT2A*, *FTH1*, and *GFAP* (Fig. 2b, Supplementary Data 1). *ID2*, a gene encoding Inhibitor of DNA Binding 2, is known to be involved in inflammatory signalling, possibly linking Astrocytic inflammatory pathways in Alzheimer's to previous research that showed under inflammatory conditions cytokines upregulate *ID2* (Fig. 2b) [15]. *GFAP* is a well-established astrocyte marker and has been used as an Alzheimer's biomarker [16]. The identification of well-characterized AD-related genes among top features reinforces the biological validity of our modeling approach, and suggests that those high-ranking predictors with limited prior characterization may also play meaningful roles in AD.

Calibration and Brier Score Analysis

To evaluate probabilistic calibration, we computed Brier scores on held-out test data, pooled across five randomized train–test splits for each cell type and model configuration. The Brier score measures the mean squared error between predicted probabilities and observed outcomes, with lower values indicating better calibrated and more reliable probabilistic predictions. Across all models and cell types, Brier scores generally fell in the 0.20–0.30 range, consistent with moderate calibration in a challenging single-cell disease-classification setting (Supplementary Fig. 4c). Models integrating APOE genotype with gene expression exhibited the most consistent calibration across cell types, with mean Brier scores between 0.228 and 0.259 and relatively low variability across splits, indicating stable probabilistic behavior. In contrast, models combining gene expression with demographic covariates showed greater heterogeneity in calibration, including elevated variability in certain cell types. Gene-only models demonstrated intermediate calibration, while models using demographics and APOE alone showed mixed performance and poor calibration for some cell types, particularly microglia, highlighting the importance of transcriptomic features for reliable probability estimates.

Overall, these results indicate that calibration depends strongly on both cell type and feature set, with the most reliable probabilistic predictions obtained when genetic risk (APOE) is integrated with gene expression data. Calibration metrics are reported here as diagnostic measures of uncertainty and reliability, rather than as optimization targets, and should be interpreted alongside discrimination metrics such as AUC. Complete Brier score statistics for all models, cell types, and splits are provided in Supplementary Data 23.

Clinical and Pathology Predictors Diverge Across Cell Types

Considering the two key hallmarks of Alzheimer's, cognitive decline and amyloid, tau, and neurodegenerative (ATN) pathology, we suspected that gene expression and other biological

features may explain neuropathology-based AD diagnosis differentially from clinically-based AD diagnosis. We tested this hypothesis by rerunning our analysis of the Mathys et al. dataset[17], with outcomes and group labels defined by neuropathology (CERAD score 1-2) rather than clinician diagnosis. Specifically, we predicted moderate or frequent neuritic plaques based on CERAD score (i.e., CERAD: 1 or 2 as AD), a semiquantitative measure of neuritic plaque pathology. Our classifiers revealed that gene expression and APOE genotype strongly predict neuritic plaque pathology across cell types (Fig. 4a).

In Astrocytes (mean AUC: 0.691 ± 0.063), we found top predictors to include *RASGEF1B*, *MT1E*, and *GFAP* as well as *APOE*. We also observed predictive power in Excitatory Neurons (mean AUC: 0.739 ± 0.179), Inhibitory Neurons (mean AUC: 0.695 ± 0.196), and Microglia (mean AUC: 0.645 ± 0.188) (Fig. 4a). We found strong overlap between genes predictive of amyloid-plaque pathology and clinical diagnosis; however, their importance was rearranged. In Microglia, top predictors included *SAT1*, *PRKCA*, and *APOE*. *SAT1* (log2FC = -0.44, FDR p = 5.76E-08), a gene whose increased expression has previously been linked to immune cell infiltration in glioma, were downregulated in our fixed-effect DEG analysis, complementing our previous finding of IFI44L downregulation [18] (Supplementary Data 21). *PRKCA*, a gene responsible for lipopolysaccharide-induced macrophage functions, including inflammation and defense, was also downregulated (Supplementary Data 21). *APOE* was significantly upregulated, consistent with its role in advanced Amyloid pathology (Supplementary Data 21).

Overall, we demonstrate complementary findings across our predictive and fixed effects differential expression approaches for both clinical and neuropathology outcomes. First, despite cell-to-cell heterogeneity, *RASGEF1B* was observed as a top predictor of both CERAD

pathology and clinical diagnosis in all cell types. Second, we observed strong concordance between genes predictive of pathology AD and clinical AD across cell types (Fisher's $q < 1.92e-20$) (Supplementary Data 7), but especially in Microglia with 244 overlapping predictors (OR = 106.7, 95% CI: 84.4-134.9) and in Oligodendrocytes 238 overlapping predictors (OR = 33.09, 95% CI: 27.2-40.27) (Supplementary Data 7). These results suggest in Microglia and Oligodendrocytes, molecular signals of amyloid pathology may be tightly coupled to clinical manifestation of disease [19,20].

Robustness of Predictive Gene Prioritization to Updated snRNA-seq Quality Control

Mathys et al. (2019) conducted an early large-scale single-nucleus RNA-sequencing (snRNA-seq) study of Alzheimer's disease (AD), enabling the discovery of cell-type–specific transcriptional changes that are not observable in bulk RNA-seq data. Subsequent methodological work by Murphy et al., however, highlighted important sensitivities in snRNA-seq differential expression (DE) analyses to data processing, quality control, and statistical modeling choices, demonstrating that alternative, best-practice analytical pipelines can substantially alter the set of genes identified as differentially expressed. In brief, Murphy et al. applied updated quality-control procedures to the Mathys dataset, including explicit filtering of empty droplets, doublets, and nuclei with low complexity or high mitochondrial content, using quantitative cutoffs informed by contemporary best-practice guidelines. They also performed donor-level integration to mitigate batch effects across samples. These steps resulted in a smaller, higher-quality cell set for downstream analyses. These findings underscore that DEG discovery in single-cell data can be highly dependent on upstream analytical assumptions.

Given this critique, we sought to explicitly test whether the predictive gene prioritization produced by TriSCOPE is sensitive to these upstream processing and QC decisions, or whether it

identifies a more stable subset of disease-relevant genes that persists even under substantially stricter filtering and revised analytical assumptions. To this end, we reran the full TriSCOPE predictive modeling pipeline using the Murphy/Skene–processed snRNA-seq data (Murphy-48) in place of the original Mathys et al. processed data (Mathys-48), while holding all downstream modeling choices fixed (including donor-aware cross-validation, feature selection, and gradient-boosted decision tree classifiers). We then compared the resulting predictive gene rankings between the two pipelines on a cell-type–specific basis, focusing on both (i) rank concordance across all shared genes and (ii) overlap among consistently selected predictors.

Overall, our analysis of the Murphy-48 dataset recapitulated key findings from Mathys-48. Pan-cell-type predictors such as ARL17B, LINGO1, RASGEF1B, and SLC26A3 were again predictive across cell types, and cell-type specificity was preserved for PLXDC2 in Microglia, GRID2 in Oligodendrocytes, and GFAP in Astrocytes. For each major brain cell type (Astrocytes, Microglia, Inhibitory Neurons, Oligodendrocytes, OPCs, and Excitatory Neurons), we computed the Spearman rank correlation between mean normalized feature importance scores derived from the original Mathys-based pipeline and the Murphy/Skene-based pipeline, across all genes shared between the two analyses.

Despite substantial differences in upstream QC and DEG stringency, predictive gene rankings were significantly correlated across all cell types. Rank concordance was strongest in glial populations, with Spearman correlations of $\rho = 0.442$ in Microglia, $\rho = 0.440$ in Oligodendrocytes, and $\rho = 0.458$ in OPCs (all $p \ll 10^{-40}$). Excitatory neurons also showed robust concordance ($\rho = 0.345$, $p \approx 10^{-210}$), while Astrocytes exhibited more modest but still highly significant correlation ($\rho = 0.182$, $p \approx 10^{-22}$). Inhibitory neurons showed the weakest correlation ($\rho = 0.078$, $p \approx 10^{-9}$), consistent with greater transcriptional heterogeneity and lower

signal-to-noise in this population. These results indicate that, even when upstream technical processing substantially alters the DEG landscape, the relative prioritization of predictive genes remains broadly preserved, particularly in glial cell types that are strongly implicated in AD pathology.

To further assess robustness at the level of discrete gene sets, we defined predictive genes as those receiving nonzero feature importance in at least two of five cross-validation splits within each pipeline. We then quantified overlap between Mathys-derived and Murphy/Skene-derived predictor sets, reporting recall with respect to the smaller of the two sets to avoid bias from differences in predictor set size. Across cell types, we observed substantial overlap. Recall values ranged from 0.231 in Inhibitory Neurons to 0.660 in Oligodendrocytes, with intermediate values in Astrocytes (0.611), Microglia (0.637), OPCs (0.547), and Excitatory Neurons (0.442). Notably, glial populations again showed the highest concordance, with over half of the smaller predictor set recovered across pipelines in Astrocytes, Microglia, Oligodendrocytes, and OPCs. Importantly, this degree of overlap was observed despite the fact that the Murphy et al. reanalysis reported orders of magnitude fewer DEGs, underscoring that predictive modeling identifies a subset of genes that is far more stable to QC and statistical stringency choices than univariate differential expression alone.

Together, these analyses demonstrate that TriSCOPE's predictive gene prioritization is robust to major revisions in snRNA-seq preprocessing and analytical methodology, directly addressing concerns regarding the sensitivity of DEG-based findings to upstream choices. While univariate differential expression results can vary substantially with processing and modeling decisions, the multivariate, donor-aware predictive framework used here consistently recovers overlapping gene importance patterns across independently processed versions of the same

underlying data. These findings support the interpretation that predictive modeling captures coordinated transcriptional programs associated with disease state, rather than relying on marginal expression shifts that may be sensitive to specific DEG pipelines. As such, predictive feature prioritization provides a complementary and, in some respects, more stable approach to identifying disease-relevant transcriptional structure in single-cell data.

Predictor Validation in the Lau-21 cohort

To confirm the robustness of these findings, we validated our cell-type-specific classifiers on the Lau-21 dataset comprising 12 clinical AD and 9 clinical control individuals, covering 172,514 nuclei across Microglia, Oligodendrocytes, Excitatory Neurons, Inhibitory Neurons, and Astrocytes (Supplementary Data 15). Using one-shot classifiers from our original dataset, and ensuring strictly no donor-level data leakage, we showed consistent predictive performance across cell types, suggesting the generalizability of our models (Supplementary Fig. 1a). Our one-shot classifiers demonstrated consistent performance across cell types, with gene-only classifiers showing mean AUCs greater than 0.5 in Astrocytes (AUC 0.67), Oligodendrocytes (gene-only classifier mean AUC 0.58), Microglia (gene-only classifier mean AUC 0.56), Inhibitory Neurons (gene-only classifier mean AUC 0.60), and Excitatory Neurons (gene-only classifier mean AUC 0.65) (Supplementary Fig. 1b).

More significantly, upon retraining our models on the Lau et al. dataset from scratch, we observed strong concordance in top predictive genes between autoML models fit on the Lau et al. dataset and the Mathys et al. dataset, once again with cell type specificity (Supplementary Fig. 1a; Supplementary Data 10). In the models fit on Lau et al. data, these included *NLGN4Y* in Inhibitory Neurons and *MTRNR2L8* in Excitatory Neurons (Supplementary Fig. 1c,

Supplementary Data 10). Microglia showed *PLXDC2* and *APOE* as top predictors, while Astrocytes showed *COL21A1* and *GFAP*. In Oligodendrocytes we observed *CRYAB*, *FTH1*, and *APOD* to all be top predictors. We once again saw *RASGEF1B*, *ARL17B*, *LINGO1*, and *SLC26A3* as predictive across multiple cell types, reinforcing their robustness as pan-cell-type AD predictors (Supplementary Data 10, Supplementary Fig. 1c).

To further assess cross-dataset reproducibility at the level of discrete predictor selection, we quantified the overlap between Mathys (48 donors) and Lau predictor sets using a definition of predictors as genes with nonzero importance in at least two of five training splits, and measured recall relative to the smaller predictor set. Across cell types, we observed substantial but heterogeneous overlap, with higher recall in glial populations—particularly microglia (recall = 0.45) and oligodendrocytes (0.40)—and more variable overlap in neuronal populations. Notably, excitatory neurons showed high recall (0.82), reflecting strong agreement in the Lau dataset. Aggregated across cell types, 37.5% of predictors from the smaller sets were recovered across datasets, supporting partial but nontrivial reproducibility of predictive feature selection under differing cohorts and modeling pipelines (Supplementary Data 18).

Consistent with these recall estimates, Fisher's exact tests confirmed significant enrichment of shared predictors across all five major cell types (Supplementary Data 10). In Inhibitory Neurons, *RASGEF1B*, *ARL17B*, *NLGN4Y*, *SLC26A3*, and *LINGO1* were top predictors. In Excitatory Neurons top predictors were *PDE10A*, *RASGEF1B*, *NLGN4Y*, *SLC26A3*, *USP97*, *ARL17B*, *MTRNR2L8*, and *LINGO1*. Our validation analysis in Astrocytes showed top predictors as *SLC1A2*, *COL21A1*, *SLC26A3*, *RASGEF1B*, *GFAP*, and *HSPA1A*. In Microglia, *PLXDC2* and *APOE* once again emerged as cell-specific predictors (Supplementary Fig. 1c). Across the five cell types, we found strong overlap in predictive genes between our two cohorts (FDR adjusted

Fisher's $p < 0.05$ in all cell types) (Supplementary Fig. 1a, Supplementary Data 11), including 40 overlapping predictors in Astrocytes (OR = 30.28), 14 in Excitatory Neurons (OR = 560.06), 12 in Inhibitory Neurons (OR = 415.55), 122 in Microglia (OR = 40.39), and 106 overlapping in Oligodendrocytes (OR = 21.78) (Supplementary Fig. 1a, Supplementary Data 11).

Predictor Validation in ROSMAP-427

To assess the stability of TriSCOPE's predictive signal at larger within-cohort scale, we trained cell-type-specific classifiers in the ROSMAP-427 cohort (2.3 million nuclei, 427 donors) using the same gradient-boosted decision tree framework with donor-held-out cross-validation. For each cell type, we evaluated four model configurations: gene expression only (genes), gene expression with APOE genotype (genes_apoe), gene expression with all demographic covariates and APOE genotype (genes_all), and demographic covariates with APOE genotype alone (demo). For Astrocytes, Microglia, and OPCs, all cells per donor were included; for Excitatory Neurons, Inhibitory Neurons, and Oligodendrocytes, cells were capped at 400 per donor due to memory constraints.

Across all six cell types, the genes_apoe configuration achieved the highest mean AUC: Astrocytes (0.711 ± 0.027), Microglia (0.695 ± 0.030), Oligodendrocytes (0.667 ± 0.043), OPCs (0.651 ± 0.059), Excitatory Neurons (0.630 ± 0.011), and Inhibitory Neurons (0.626 ± 0.021). Notably, genes_apoe exhibited the tightest variability across splits (SD range: 0.011–0.059), while demo showed the widest (SD range: 0.049–0.061), indicating that the improvement from incorporating gene expression with APOE is robust to split selection rather than noise-driven. Gene expression alone outperformed the demographic-only model in Astrocytes (0.677 vs. 0.539), Microglia (0.599 vs. 0.574), Oligodendrocytes (0.624 vs. 0.607), and OPCs (0.586 vs.

0.545), while in Excitatory Neurons (0.567 vs. 0.572) and Inhibitory Neurons (0.548 vs. 0.591) the two configurations performed comparably. The combined model (genes_all), which included gene expression, demographics, and APOE genotype, did not improve upon genes_apoe in any cell type, suggesting that the addition of demographic covariates beyond APOE introduces noise rather than complementary signal when gene expression is already included.

To assess whether the same molecular programs emerge at 10× the cohort scale, we quantified PEG overlap between Mathys-48-derived and ROSMAP-427-derived predictor sets. Significant overlap was observed in all six cell types (Fisher's exact test, FDR-corrected): Microglia shared 85 predictive genes (466 Mathys-48, 255 ROSMAP-427; fold = 3.43, $p = 2.64 \times 10^{-26}$), Oligodendrocytes shared 62 (621 Mathys-48, 179 ROSMAP-427; fold = 3.02, $p = 2.14 \times 10^{-16}$), OPCs shared 45 (835 Mathys-48, 147 ROSMAP-427; fold = 2.77, $p = 1.00 \times 10^{-10}$), Astrocytes shared 40 (175 Mathys-48, 349 ROSMAP-427; fold = 3.53, $p = 1.19 \times 10^{-12}$), Excitatory Neurons shared 5 (162 Mathys-48, 11 ROSMAP-427; fold = 32.89, $p = 2.46 \times 10^{-7}$), and Inhibitory Neurons shared 2 (108 Mathys-48, 10 ROSMAP-427; fold = 17.96, $p = 5.21 \times 10^{-3}$) (Supplementary Data 24).

Among shared predictors, key genes from our discovery analyses were independently recovered at larger scale. In Microglia, shared PEGs included *APOE*, *BIN1*, *FKBP5*, *IFI44L*, *HS3ST4*, *HSPA1A*, *HSPA1B*, *HSP90AA1*, *HIF1A*, *INPP5D*, *CLEC7A*, *DOCK4*, *DOCK8*, *ERC2*, and *KANSL1*. In Oligodendrocytes, shared PEGs included *ARL17B*, *CRYAB*, *LINGO1*, *FKBP5*, *GRID2*, *HSPA1A*, *NCAM2*, *NRXN3*, and *OPALIN*. In Astrocytes, shared PEGs included *FTH1*, *LINGO1*, *KCNIP4*, *KANSL1*, *MERTK*, and metallothioneins *MT1E*, *MT1G*, *MT1M*, and *MT3*. In OPCs, shared PEGs included *EGFR*, *APOD*, *FTH1*, *KCNIP4*, *B2M*, *ERBB4*, *P2RX7*, and

SLC26A3. In Excitatory Neurons, shared PEGs included *PDE10A* and *RALYL*, and in Inhibitory Neurons, *LINGO1* and *SLC26A3*.

These results demonstrate that the same cell-type-specific PEGs nominated in our 48-donor discovery cohort are independently recovered when the pipeline is scaled to the full 427-donor ROSMAP release. The consistency of both the genes_apoe performance advantage and the cross-scale PEG overlap replicates the pattern observed in the Mathys-48 discovery cohort and in SEA-AD, supporting the robustness and generalizability of TriSCOPE's predictive framework.

Fixed-Effect DEG Analysis

We further evaluated sensitivity to modeling assumptions by comparing donor-aware random-effects DE models to two separate fixed-effects models. The first, was a fixed-effects model controlling for age, sex, and post-mortem interval. Log2 fold-change estimates were highly concordant across all cell types (Spearman $\rho = 0.77$–0.87), indicating that inferred transcriptional effect sizes are robust to model specification while random effects appropriately regulate statistical significance. The following section reports DEG results from our first fixed-effects model.

In Excitatory Neurons, two of our top predictors *LINGO1* and *RASGEF1B*, were both differentially expressed in Alzheimer's disease (AD) cases. LINGO1 showed significant upregulation ($log_2FC = 0.28$, FDR = 2.3E-209) and RASGEF1B was also marginally upregulated ($log_2FC = 0.26$, FDR = 1.6E-179) (Supplementary Data 21). In addition, *MTRNR2L1* ($log_2FC = 1.39$, FDR = 2.45E-82) was significantly upregulated and *MTRNR2L8* was significantly downregulated ($log_2FC = –0.39$, FDR = 2.44E-17) (Supplementary Data 21). While differential expression analysis revealed over 1,081 genes altered in AD within Excitatory Neurons, a much

smaller subset of only 162 features were prioritized (24 overlapping) by our predictive modeling analyses, highlighting a much more focused core of disease-relevant changes when using a multivariate machine learning approach as opposed to a univariate differential expression approach. In Inhibitory Neurons, 17 of our predictive features were significantly differentially expressed in Alzheimer's disease (AD) cases. Though we identified 252 DEGs, many of our highly predictive features — including *AHI1*, *USP9Y*, *NLGN4Y*, and *UTY* — did not meet differential expression thresholds (FDR < 0.05) (Supplementary Data 21). An exception was SLC26A3, which was significantly upregulated (FDR = 1.38E-27, FDR = 0.40) (Supplementary Data 21)

In Microglia, several top predictive genes were also significantly differentially expressed between Alzheimer's disease (AD) and control samples. *IFI44L*, the highest-ranking predictor, was significantly downregulated (log_2FC = –1.21, FDR = 0.0006), consistent with prior evidence implicating impaired type I interferon signaling, and reduced expression of interferon-stimulated genes like *IFI44L*, in individuals at elevated risk of AD progression [21]. SRGN, another strong immune-related predictor, was markedly downregulated (log_2FC = –1.30, FDR = 6.78E–19) (Supplementary Data 21). *HSPA1A***, a** chaperone gene associated with protein quality control, was also significantly reduced (log_2FC = –0.57, FDR = 3.56E-05), alongside upregulation of APOE (log_2FC = 0.36, FDR = 0.009). In contrast, PLXDC2 and PTEN—both of which showed predictive importance—were not significantly differentially expressed.

In Oligodendrocytes, *HSPA1A* — one of the top predictive features — was significantly downregulated in Alzheimer's disease (AD) cases, showing robust downregulation (log_2FC = -0.55, FDR = 2.34E-35) (Supplementary Data 21). In total, we observed 92 predictive and differentially expressed genes, with 16% of predictive genes being DEGs. In contrast, other

top-ranked predictors such as *APOD* were moderately differentially expressed, while *CRYAB* was not differentially expressed (Supplementary Data 21). These results highlight the potential of predictive modeling to uncover disease-relevant features that may not exhibit strong univariate shifts but could participate in multivariate, context-dependent expression patterns in AD. In Oligodendrocyte Progenitor cells (OPCs), two of the top predictive features, *FTH1* and *APOD*, were also significantly differentially expressed in Alzheimer's disease (AD) cases. *FTH1* was upregulated with a $\log_2$ fold change ($\log_2$FC) of 0.37 (FDR = 8.41E-06), and *APOD* similarly showed significant upregulation ($\log_2$FC = 0.27, FDR = 2.54E–08) (Supplementary Data 21). In Astrocytes, *MT2A* and *GFAP*, were significantly differentially expressed in Alzheimer's disease (AD) cases. *MT2A* was also strongly upregulated ($\log_2$FC = 0.57, FDR = 3.82E-40). *GFAP*, a known astrocytic marker, exhibited significant upregulation ($\log_2$FC = 0.45, FDR = 1.93E-11) (Supplementary Data 21).

Overall, we have identified robust transcriptional changes in a cell-type specific manner between Alzheimer's patients and controls, defined by clinical adjudication. Our prior analyses of predictive genes highlighted genes with varying overlap with DEGs (2.75-32% of predictive genes were DEGs in each cell type). Together, these findings demonstrate that while differential expression analysis captures many transcriptional changes in Alzheimer's disease (AD), our predictive modeling approach identifies a complementary but often non-overlapping set of biologically meaningful features. In most cases, these include genes that are subtly dysregulated or fall beneath conventional significance thresholds but may reflect important disease mechanisms—such as sex-specific vulnerability programs in Inhibitory Neurons or immune-related regulatory shifts in Microglia. These results reinforce the ability of predictive modeling to nominate both strongly altered and more nuanced transcriptional signals involved in

AD neuropathology, providing additional insight into the cell-type–specific molecular architecture of disease.

Due to the lack of a random effect, we sought to validate these single-cell differential expression results against a bulk RNA-seq standard. We first generated pseudobulk profiles using Scanpy (v1.10.3). Within each broad cell type, raw UMI counts were summed across all cells from the same donor to create donor-level count matrices, retaining only donors with at least one cell in the given cell type. Gene expression was then filtered to retain genes with counts per million (CPM) ≥ 1 in at least 10% of donors (minimum of three donors). For each cell type, we fitted a linear model using the limma-voom pipeline (edgeR v4.4.2, limma v3.62.2), with Alzheimer's disease (AD) status as the primary variable of interest and adjusting for the same covariates used in the single-cell models. Continuous covariates were mean-centered and scaled prior to model fitting. In parallel, we generated a "bulk-like" dataset by summing pseudobulk counts across all major cell types for donors with matched profiles across cell types, followed by the same limma-voom analysis. For both the per–cell-type and bulk-like analyses, moderated t-statistics for AD status were extracted to assess effect directionality and magnitude. We observed that the direction of log-fold changes in pseudobulk analyses generally matched those from our single-cell–level models, supporting the robustness of our single-cell findings to a bulk RNA-seq framework. Directionality agreement between single-cell and bulk differential expression results was 73.81% for astrocytes, 86.85% for excitatory neurons, 75.40% for inhibitory neurons, 57.73% for microglia, 76.86% for oligodendrocytes, and 78.72% for OPCs.

As an additional sensitivity analysis, we directly compared $\log_2$ fold-change estimates obtained from donor-aware random-effects models to those from fixed-effects models that explicitly controlled for sequencing batch and APOE genotype. Across all major cell types,

effect size estimates were highly concordant between the two modeling strategies (Spearman ρ = 0.63–0.78; all $p < 1\times10^{-16}$; n = 10,807–17,017 genes per cell type), indicating that inferred transcriptional effect sizes are largely insensitive to whether donor structure is modeled via random intercepts or through explicit batch and genotype covariates. Concordance was strongest in excitatory neurons (ρ = 0.78) and astrocytes (ρ = 0.72), with similarly high agreement observed in oligodendrocytes, OPCs, inhibitory neurons, and microglia. These results demonstrate that while random-effects modeling is essential for appropriate variance partitioning and statistical calibration, the underlying biological signal captured by differential expression—namely the direction and relative magnitude of disease-associated transcriptional changes—is robust to alternative approaches for handling donor- and batch-level confounding.

Discordance and concordance between predictive gene importance and differential expression

We assessed concordance between TriSCOPE-derived predictive gene rankings and differential expression results across multiple cohorts and analytical frameworks.

In our discovery cohort (Mathys et al. 2019, n = 48 donors), predictive gene rankings were positively and significantly correlated with DEG rankings defined using FDR-adjusted p-values (Spearman ρ = 0.04–0.48; all FDR < 0.005), with the strongest agreement observed in microglia. The magnitude of correlation varied substantially by cell type, ranging from weak but significant concordance in inhibitory neurons to moderate–strong concordance in glial populations. This partial alignment indicates that while TriSCOPE-derived predictors are biologically anchored in differential expression, predictive modeling captures multivariate disease-associated structure that is not explained by univariate differential expression alone.

To further evaluate concordance in an independent and larger cohort, we performed expanded donor-aware differential expression analyses in a larger ROSMAP cohort comprising 427 individuals. Across cell types, 2–8% of predictor genes were differentially expressed, indicating limited but non-random overlap between predictive features and donor-aware DEGs. Consistent with this, predictors showed significant enrichment among DEGs in multiple cell types (odds ratios ranging from ~2.8 to 6.9 in enriched cell types), while no enrichment was observed in inhibitory neurons and OPCs (Supplementary Data 18). In parallel, predictive importance exhibited a statistically significant but weak association with differential expression effect sizes across five of six cell types, with the strongest associations observed in Microglia and Oligodendrocytes. Spearman correlations between predictor rank and absolute log fold change were modest in magnitude ($\rho \approx 0.02$–0.21), indicating that although TriSCOPE predictors are enriched among DEGs in a subset of cell types, their predictive importance is not reducible to transcriptional effect size alone (Supplementary Data 18).

We observed a similar qualitative pattern when comparing TriSCOPE predictors to differential expression rankings from four previously published single-cell Alzheimer's disease studies (Morabito et al.[22], Sadick et al.[23], Su et al.[24], and Grubman et al.[25]) spanning multiple brain regions, collectively comprising 94 donors. Modest rank concordance was observed in glial cells (mean Spearman $\rho \approx 0.26$ in microglia, 0.18 in oligodendrocytes, 0.30 in OPCs) and moderate concordance in excitatory neurons (mean Spearman $\rho = 0.235$), with weaker correlations in inhibitory neurons and astrocytes (Supplementary Data 18). Across all analyses, our results included over 540 unique individuals, reinforcing that TriSCOPE predictors reflect stable, multivariate disease signals that are partially but not fully explained by univariate differential expression across cohorts, brain regions, and analytical pipelines.

Neuropathological versus Clinical AD Differential Expression

To better understand these differences, we performed differential expression analysis between individuals who exhibited Alzheimer's pathology, but no clinical diagnosis, and those with Alzheimer's pathology and clinical diagnosis. Our analysis revealed downregulation of *SRGN* and *HIF1A* in Microglia, and subsequent pathway analysis revealed enrichment of pathways related to neuroinflammatory response and cytokine production and regulation (Supplementary Fig. 2d). Similar analysis in Oligodendrocytes revealed upregulation of pathways related to ion transport and protein localization (Supplementary Fig. 2d).

Concordance between machine learning–derived pathway enrichment and differential expression–based pathway analysis

To evaluate whether pathways identified by machine-learning–derived feature importance reflected underlying disease biology rather than model-specific artifacts, we compared gene set enrichment results obtained from ML-based GSEA (Supplementary Fig. 1d) with enrichment results derived independently from differentially expressed genes (Supplementary Fig. 1e). Both analyses used an identical pre-ranked GSEA framework and only differ in the underlying gene sets being interrogated. Across cell types, the two approaches showed substantial qualitative concordance in the functional categories identified, despite relying on orthogonal signals—predictive importance across randomized splits versus random effects modeling-based differential expression.

In multiple cell types, both analyses converged on RNA-binding and transcription-related functions, as well as metal-ion and zinc-ion binding pathways, suggesting shared regulatory and

post-transcriptional processes associated with disease state. Ubiquitin-related activities and protein modification pathways were observed in both ML-derived and DEG-based analyses in neurons, oligodendrocytes, and OPCs, while kinase- and GTPase-related functions appeared consistently in microglia across both frameworks. Importantly, while the relative ranking and statistical strength of individual terms differed—as expected given differences in gene selection criteria and statistical modeling—the recurrence of overlapping functional themes across independent analytical strategies supports the robustness of these biological signals. Together, this concordance indicates that the pathways highlighted by the classifiers largely reflect genuine disease-associated molecular programs captured by differential expression, rather than spurious correlations introduced by model fitting.

GWAS Integration

We tested whether genome-wide significant AD single-nucleotide polymorphisms SNPs from the NHGRI-EBI GWAS Catalog exert functional effects on transcriptomic predictors of both clinical and pathological AD by identifying rsIDs that (i) map to model-nominated genes, and (ii) checking if those exact SNPs were eQTLs in human brain tissue.

Clinical AD predictors in Inhibitory Neurons, Microglia, Oligodendrocyte Progenitor Cells, and Oligodendrocytes were significantly enriched for genes containing genome-wide significant SNPs (Fisher's p range=1.78e-5 - 0.047). Our analyses revealed pan-cell-type genetic convergence, including LINGO1 (rs12902811) (-log10 p = 13.7) and ARL17B (rs2732703) (-log10 p = 8.22) (Supplementary Fig. 5a, Supplementary Data 5). The gene in Oligodendrocytes with the strongest association with AD was *CLU* (rs11787077) (-log10p=43.7), and *CLU* gene expression levels have been implicated in cognitive decline in Alzheimer's [26] (Supplementary

Data 5). Microglia showed 68 predictive overlapping GWAS genes (OR = 1.48, Fisher's p = 0.047), including *APOE* (-log10p = 3.2E+02), *BIN1* (1.2E+02), *PICALM* (rs561655) (-log10p = 26), and *RASGEF1C* (rs113706587) (-log10 p = 15.7) (Supplementary Fig. 5a, Supplementary Data 5). ABCA1 was a top GWAS hit in Astrocytes (-log10p = 32). To further examine functional relevance, we assessed whether these predictive GWAS-associated genes were also regulated at the transcriptional level using the GTEx brain tissue eQTL databases [27]. We found that across all cell types, 5 SNPs had both eQTL's in prefrontal cortex relevant tissue and were GWAS associated genes functionally predictive of Alzheimer's. Of these, *APOE* (rs769446), *CEP170* (rs71537331), *CTSB* (rs1065712), and *PICALM* (rs17745409) were predictors in Microglia, while *CLU* (rs4236673) was predictive in Oligodendrocytes and Excitatory Neurons. *CLU* and *PICALM* (rs4236673) were predictive in Oligodendrocyte Progenitor Cells.

We repeated this overlap analysis using predictors of AD neuropathology rather than clinical diagnosis, which revealed *APOE* as the top hit in both Astrocytes and Microglia (Supplementary Fig. 5b, Supplementary Data 6). We once again found the strongest overlap with GWAS hits of 106 genes in Oligodendrocytes (OR = 1.83, Fisher's p = 1.90E-07) (Supplementary Data 6). We then examined the overlap between clinical and pathological AD predictive genes that were also genome-wide significant in the GWAS catalog. In Excitatory Neurons, all pathology-associated GWAS hits also appeared among clinical AD GWAS hits and showed LINGO1 to be a shared GWAS hit in every cell type (Supplementary Fig. 5c). *CLU* was shown to be only present in the clinical AD GWAS set, suggesting a potential bias toward cognitive AD phenotypes. *APOE* overlapped between pathology- and clinical-AD GWAS hits in Microglia, but was only observed in the pathology-associated GWAS set in Astrocytes. Our CERAD predictors also showed highly significant overlap (Fisher's p = 1.36E-245) with brain

tissue eQTL's and revealed similar sets of genes. Our analysis thus reveals that there is heterogeneity among the functional effects of GWAS associated genes at the mRNA level, suggesting that GWAS-associated genes may exert differential, cell-type-specific effects, and may variably influence clinical or pathological manifestations of AD. We then took these GWAS associated pathological predictors and checked for brain tissue eQTLs, revealing 5 SNPs. These included *APOE* in Astrocytes, *APOE* and *PICALM* in Microglia, *CEP170* and *CEP63* in Inhibitory Neurons, and *ARFGEF3* in OPCs. By finding direct rsID overlap, we establish a functionally interpretable link between these previously GWAS associated genes and gene expression that is predictive of Alzheimer's disease. Furthermore, heterogeneity between Clinical and Pathological Alzheimer's predictors, may hint at differing stages or manifestations of disease severity.

Integration with GWAS and brain eQTL datasets provided support for many predictors, strengthening their biological evidence. One-to-one SNP-to-gene mapping allowed direct alignment of predictive genes with genome-wide significant AD loci. In microglia, *APOE* and *PICALM* overlapped with pathology-associated predictors, pointing to amyloid clearance and plaque-associated immune pathways. Clinical predictors in Oligodendrocyte Progenitor Cells included CLU, implicated in amyloid processing and cognitive decline, while astrocyte predictors included ABCA1, a lipid transporter linked to cognitive deficits in mice models [28]. These cell-type-specific overlaps link genetic susceptibility loci to transcriptional states in a biologically interpretable way, nominating high-confidence targets for mechanistic follow-up. Finally, pseudotime analysis at the subcluster level revealed progressive transcriptional trajectories predictive of AD. Key genes such as *RASGEF1B*, *SLC26A3*, and *LINGO1* defined the disease trajectory across multiple subclusters validating their significance, and pseudotime

states were positively associated with amyloid formation, impaired clearance, and neuroinflammatory pathways, highlighting transcriptional progression within discrete neuronal and glial subpopulations and suggesting potential key markers of vulnerability [29].

eQTL Overlap analysis

To evaluate whether predictive genes were likely under genetic regulatory control, we assessed their overlap with cis-eQTLs identified in human brain tissue. We downloaded version 10 eQTL data from the Genotype-Tissue Expression (GTEx) project for three AD-relevant tissues: Brain – Cortex, Brain – Anterior cingulate cortex (BA24), and Brain – Frontal Cortex (BA9). We extracted Ensembl gene IDs with significant eQTLs (FDR < 0.05) and mapped these to gene symbols using a reference gene annotation file. Predictive genes were matched to eQTL genes based on shared gene symbols, and overlaps were identified per cell type. As with GWAS analysis, the background for Fisher's exact tests was the full RNA-seq-expressed gene set (17,926 genes). To visualize eQTL support across cell types and predictive genes, we constructed a binary heatmap of the top predictive genes per cell type, indicating whether each gene had eQTL support in the three GTEx brain tissue. GWAS integration was performed by direct matching of exact genome-wide significant SNP rsIDs to model-nominated genes and brain tissue eQTL targets. Enrichment statistics (Fisher's test p-values and odds ratios) were calculated for each cell type and for both clinical and CERAD-based models.

Subcluster Profiles Reveal Transcriptional Gradients of AD Progression

To more comprehensively characterize the extent to which transcriptional trajectories capture Alzheimer's disease (AD)–associated variation across cellular subpopulations, we systematically evaluated the predictive performance of subcluster-specific pseudotime for both clinical AD diagnosis and neuropathological burden (CERAD score) across a range of discrimination thresholds (Supplementary Data 14). Pseudotime trajectories were inferred independently within each of the 41 subclusters defined by Mathys et al. using highly variable genes selected without reference to disease status, enabling an unbiased assessment of disease-associated transcriptional progression.

At a stringent threshold (AUC > 0.60), pseudotime robustly predicted clinical AD in only two subclusters, both of which were glial. Specifically, an oligodendrocyte subcluster (Oli3) exhibited strong discrimination (AUC = 0.699) with a large effect size (OR = 54.19, 95% CI: 39.98–73.46), while an astrocyte subcluster (Ast2) showed moderate discrimination (AUC = 0.623; OR = 22.22, 95% CI: 4.99–98.97). At the same threshold, pseudotime predicted neuropathological AD in Oli3 (AUC = 0.714; OR = 76.26, 95% CI: 50.09–116.11) and in a distinct oligodendrocyte progenitor population (Opc2; AUC = 0.654; OR = 161.18, 95% CI: 0.71–36,618.77). Although the effect size estimate for Opc2 was unstable, reflecting limited effective sample size, the consistent and strong performance of Oli3 across both clinical and pathological endpoints identifies this subcluster as a high-confidence disease-associated transcriptional trajectory.

At a moderate discrimination threshold (AUC > 0.55), pseudotime predictivity extended to a broader but still selective set of subclusters. For clinical AD, seven subclusters spanning seven cell types met this criterion, including astrocytes (Ast2), endothelial cells (End1), excitatory neurons (Ex11), microglia (Mic1), oligodendrocytes (Oli3), oligodendrocyte progenitors (Opc2),

and pericytes (Per). For neuropathological AD, eight subclusters across five cell types were predictive, including astrocytic (Ast2, Ast3), excitatory neuronal (Ex11, Ex3), microglial (Mic1), oligodendrocyte (Oli3), and OPC (Opc2, Opc1) populations. Effect sizes at this threshold were heterogeneous, ranging from modest (OR ≈ 3–7) to large in select glial subclusters, reinforcing the view that disease-associated transcriptional progression is unevenly distributed across cellular contexts. We note that although pericytes were included in this descriptive analysis, they were excluded from the main predictive modeling framework due to very low sample size, and their inclusion here should be interpreted cautiously.

At a permissive threshold (AUC > 0.50), weak but detectable disease-associated trajectories were observed across the majority of subclusters, encompassing 21 subclusters spanning eight cell types for clinical AD and 22 subclusters spanning six cell types for neuropathological AD. At this level, effect sizes were generally modest and confidence intervals frequently overlapped unity, consistent with diffuse, low-magnitude transcriptional shifts rather than strongly discriminative trajectories. These results indicate that while AD-related transcriptional variation is widespread across cell types, strong and interpretable disease-associated trajectories are restricted to a subset of subclusters, with the most consistent and robust effects observed in glial lineages.

To identify genes capturing shared disease-associated transcriptional gradients, we next quantified the recurrence of trajectory-influencing genes across subclusters. Several less well-characterized genes appeared repeatedly among the top trajectory loadings across multiple cell types, including NKAIN2, GPC6, KCNIP4, HSP90AA1, FTH1, LRP1B, TMEFF2, and SGCZ, each of which was identified in four or more subclusters spanning neuronal and glial populations. Among more established AD-associated genes, RASGEF1B and LINGO1 were the

most recurrent overall, appearing across both neuronal and glial subclusters, consistent with a broadly conserved disease-associated transcriptional axis. Canonical AD genes exhibited expected cell-type specificity: APOE and GFAP were restricted to astrocytic trajectories, while CLU appeared predominantly within excitatory neuronal subclusters. Importantly, these genes were identified through unsupervised trajectory inference using highly variable genes rather than differential expression testing, supporting their role as markers of continuous disease-associated transcriptional progression rather than discrete case–control effects.

DrugBank annotation of predictive genes

To further assess the experimental tractability of model-identified genes, we systematically annotated predictive genes against the full DrugBank database. Predictive genes were defined per cell type as those assigned non-zero feature importance in at least two of five independent donor-held-out model splits, yielding 485 unique gene–cell-type pairs. These genes were queried against DrugBank v5 using the complete XML schema, capturing human drug–gene relationships across targets, enzymes, transporters, and carriers. Across cell types, 18–30% of predictive genes had at least one DrugBank annotation, with the highest coverage observed in excitatory neurons (29.6%) and astrocytes (22.9%) (Supplementary Data 19). Among manuscript-emphasized genes, eight showed DrugBank associations, including *EGFR, HSP90AA1, FTH1, APOE, ABCA1, CLU, MAPT,* and *TNFRSF1B*, spanning multiple cell types. Notably, *EGFR* and *HSP90AA1* were linked to extensive pharmacologic repertoires, while *APOE, ABCA1, CLU, MAPT*, and *TNFRSF1B* mapped to smaller but well-defined sets of compounds. These findings indicate that although many predictive genes lack existing drug annotations, a subset of high-confidence genes is directly amenable to pharmacologic perturbation, enabling future targeted experimental validation of model-derived hypotheses.

Independent validation in the Seattle Alzheimer's Disease Brain Cell Atlas (SEA-AD)

To rigorously evaluate the generalizability of TriSCOPE findings beyond ROSMAP cohorts, we applied all three analytical prongs to the Seattle Alzheimer's Disease Brain Cell Atlas (SEA-AD), an independent snRNA-seq dataset from the prefrontal cortex comprising 1,395,601 nuclei from 83 donors (41 controls, 39 dementia, 3 neurotypical). No donors overlap with any ROSMAP cohort. SEA-AD subclusters were mapped to Mathys-defined annotations using a centroid-based approach to enable direct gene-level comparison.

SEA-AD cell-type-specific classification

Cell-type-specific classifiers were trained in the SEA-AD cohort using identical modeling procedures as in ROSMAP (gradient-boosted decision trees with donor-held-out cross-validation). We evaluated four model configurations per cell type: Demographics+APOE, Genes-only, APOE+Genes, and Combined (Demographics+APOE+Genes). Full results are reported below.

In Microglia, the APOE+Genes model achieved the highest mean AUC (0.738 ± 0.031), outperforming Genes-only (0.724 ± 0.024), Combined (0.635 ± 0.118), and Demographics+APOE (0.493 ± 0.125). In Inhibitory Neurons, APOE+Genes likewise performed best (0.709 ± 0.043), followed by Genes-only (0.653 ± 0.076), Combined (0.624 ± 0.098), and Demographics+APOE (0.560 ± 0.084). In Excitatory Neurons, APOE+Genes achieved 0.670 ± 0.039, with Genes-only at 0.666 ± 0.057, Combined at 0.618 ± 0.080, and Demographics+APOE at 0.511 ± 0.104. In Astrocytes, APOE+Genes led (0.657 ± 0.138), followed by Combined (0.622 ± 0.112), Genes-only (0.620 ± 0.035), and Demographics+APOE (0.500 ± 0.161). In Oligodendrocytes, the Genes-only model achieved the highest AUC (0.660 ± 0.052), exceeding

Combined (0.598 ± 0.130), APOE+Genes (0.597 ± 0.046), and Demographics+APOE (0.548 ± 0.149). In OPCs, the Combined model achieved the best performance (0.704 ± 0.071), followed by Demographics+APOE (0.678 ± 0.108), Genes-only (0.659 ± 0.055), and APOE+Genes (0.650 ± 0.106). The OPC result was the sole exception to the general pattern in which gene expression with APOE genotype outperformed all other configurations.

Cross-cohort overlap of predictive genes

To quantify cross-cohort reproducibility at the gene level, we computed the overlap between ROSMAP-derived and SEA-AD-derived predictor sets using Fisher's exact tests (background: 19,011 expressed genes). Predictive genes were defined as those with nonzero feature importance in at least two of five cross-validation splits.

Microglia showed the largest absolute overlap with 81 shared predictors out of 466 ROSMAP and 559 SEA-AD predictors (OR = 7.952, $p = 1.862 \times 10^{-39}$). Key shared genes included *APOE*, *ARL17B*, *BIN1*, *CTSB*, *FKBP5*, and *SPP1*. Oligodendrocytes shared 11 predictors (621 ROSMAP, 25 SEA-AD; OR = 23.669, $p = 1.217 \times 10^{-10}$), including *ARL17B*, *FKBP5*, *NRXN3*, *OPALIN*, and *GRID2*. Astrocytes shared 7 predictors (175 ROSMAP, 40 SEA-AD; OR = 23.741, $p = 7.167 \times 10^{-8}$): *ARL17B*, *FTH1*, *KCNIP4*, *MRPS6*, *SLC14A1*, *SYTL4*, and *UTY*. Excitatory Neurons shared 4 predictors (162 ROSMAP, 16 SEA-AD; OR = 39.741, $p = 8.537 \times 10^{-6}$): *ARL17B*, *PDE10A*, *RALYL*, and *SYT1*. OPCs shared 7 predictors (835 ROSMAP, 29 SEA-AD; OR = 6.976, $p = 2.054 \times 10^{-4}$): *AGAP1*, *B2M*, *CD81*, *HSP90AA1*, *KIF19*, *PARD3*, and *ZFPM2*. Inhibitory Neurons shared 2 predictors (108 ROSMAP, 24 SEA-AD; OR = 16.193, $p = 8.134 \times 10^{-3}$): *ARL17B* and *TARBP1*.

Cross-cohort overlap of differentially expressed genes

Donor-aware differential expression analysis was performed in SEA-AD using the same Poisson GLMM framework with RUVSeq employed in ROSMAP. Cross-cohort DEG overlap was assessed using Fisher's exact tests (background: 19,011 genes).

Excitatory Neurons showed the largest absolute overlap (155 shared DEGs from 1,066 ROSMAP and 1,360 SEA-AD DEGs; OR = 2.364, $p = 5.022 \times 10^{-18}$). Oligodendrocytes shared 72 DEGs (711 ROSMAP, 392 SEA-AD; OR = 6.331, $p = 7.217 \times 10^{-30}$). Astrocytes shared 34 DEGs (178 ROSMAP, 803 SEA-AD; OR = 5.546, $p = 1.111 \times 10^{-13}$). Inhibitory Neurons shared 27 DEGs (240 ROSMAP, 566 SEA-AD; OR = 4.288, $p = 3.355 \times 10^{-9}$). Microglia shared 19 DEGs (88 ROSMAP, 398 SEA-AD; OR = 13.473, $p = 1.938 \times 10^{-14}$), including *APOE*, *SPP1*, *SRGN*, *CD81*, *PFKFB3*, *SNCA*, and *VSIG4*. OPCs shared 10 DEGs (64 ROSMAP, 199 SEA-AD; OR = 18.379, $p = 1.167 \times 10^{-9}$): *HSP90AA1*, *MARCKS*, *MARCKSL1*, *MT2A*, *MT3*, *NFKBIA*, *OLIG1*, *OLIG2*, *TLE1*, and *TMEM100*.

Overlap between predictive genes and DEGs within SEA-AD

Within the SEA-AD cohort, we also assessed the internal overlap between ML-derived predictors and donor-aware DEGs. The number of genes that were both predictive and differentially expressed varied by cell type: Microglia (97), Astrocytes (18), Oligodendrocytes (14), Inhibitory Neurons (7), OPCs (6), and Excitatory Neurons (5). This pattern mirrors findings in ROSMAP, where limited but non-random overlap between predictors and DEGs was observed, further supporting the conclusion that multivariate predictive modeling captures disease-relevant signals beyond what is identified by univariate differential expression alone.

Replication of genetically anchored predictors not identified by differential expression

A key finding of our discovery analyses was that a subset of genetically anchored predictive genes were systematically missed by differential expression (predictive importance in ≥2/5 splits, PP.H4 > 0.8, but not differentially expressed). In the SEA-AD cohort, *ARL17B* replicated as a predictor in four cell types (Astrocytes, Microglia, Inhibitory Neurons, and Oligodendrocytes) while remaining absent from DEG lists, directly confirming its status as a genetically supported, predictive gene that is invisible to univariate DE across independent cohorts. *JAZF1* similarly replicated as a gene that had predictive importance in ≥2/5 splits and PP.H4 > 0.8, but was not differentially expressed in Microglia. These findings provide independent confirmation that GWAS–eQTL colocalization combined with predictive modeling identifies a class of disease-relevant genes that would be overlooked by standard transcriptomic analyses.

SEA-AD pseudotime analysis and cross-cohort trajectory overlap

Diffusion pseudotime analysis was performed in SEA-AD subclusters using the same framework applied to ROSMAP, with trajectories constructed from highly variable genes selected independently of disease status. Pseudotime-based classification of neuropathological burden (CERAD) identified Ast1 as the most discriminative subcluster (AUC = 0.966), followed by Opc2 (AUC = 0.724) and Oli3 (AUC = 0.640). For clinical AD, the top subclusters were Ast1 (AUC = 0.755), Oli3 (AUC = 0.649), and Ast3 (AUC = 0.601).

Cross-cohort overlap of trajectory-influencing genes was assessed using Fisher's exact tests (background: 19,011 genes). Inhibitory Neurons showed the strongest overlap (20 shared genes from 92 ROSMAP and 101 SEA-AD trajectory genes; OR = 64.602, $p = 2.689 \times 10^{-27}$). Shared inhibitory neuron trajectory genes included *ASIC2*, *CCK*, *CNTN5*, *DCC*, *FSTL5*, *GRIA4*, *GRID2*,

KIRREL3, *LRRC4C*, *LUZP2*, *NCAM2*, *NXPH1*, *PTPRT*, *RELN*, *SGCZ*, *SORCS1*, *TMEM132D*, *TMTC2*, *UNC5D*, and *ZBTB20*.

Excitatory Neurons showed the second-strongest overlap (14 shared genes from 93 ROSMAP and 103 SEA-AD trajectory genes; OR = 37.492, $p = 7.848 \times 10^{-17}$). Shared excitatory neuron trajectory genes included *ARHGAP15*, *CCBE1*, *CDH13*, *DCC*, *GPC6*, *GRIK1*, *KCNH7*, *NRG1*, *PCDH7*, *ROBO2*, *RORA*, *RORB*, *TSHZ2*, and *UNC5D*.

In Microglia, 1 trajectory gene overlapped (40 ROSMAP, 39 SEA-AD; OR = 12.775, p = 0.079), and in Oligodendrocytes, 1 gene overlapped (37 ROSMAP, 34 SEA-AD; OR = 15.944, p = 0.064); neither reached statistical significance. No trajectory gene overlap was observed in Astrocytes or OPCs. The strong replication of trajectory-influencing genes in neuronal populations, particularly the recovery of genes involved in axon guidance (*DCC*, *ROBO2*, *UNC5D*), synaptic signaling (*GRIA4*, *GRID2*, *NRG1*), and cell adhesion (*RELN*, *CDH13*, *NCAM2*), provides independent validation that these trajectory programs reflect reproducible disease-associated transcriptional progression rather than cohort-specific artifacts.

Simulation analysis of divergence between differential expression and predictive feature importance

To provide a formal basis for the observed discordance between differentially expressed genes (DEGs) and predictively expressed genes (PEGs), we performed a count-based RNA-seq simulation designed to isolate specific biological and statistical mechanisms that can cause the two analyses to disagree. The simulation is not intended to recapitulate the complexity of real

single-nucleus transcriptomic data, but rather to demonstrate that divergence between DE and ML feature importance is expected under well-characterized conditions and does not imply that either analysis is incorrect.

We generated negative-binomial count data for 305 genes across 320 samples (80 per sex × disease stratum), with sample-specific library size variation and per-gene baseline expression and dispersion parameters drawn from distributions calibrated to approximate bulk RNA-seq. Covariates included age (continuous, normally distributed) and sex (binary). Genes were assigned to one of seven categories, each encoding a distinct mechanism: (i) *Redundant DE genes* (n = 25), which carried a genuine disease-related mean shift (log-fold change ≈ 0.42 on the log scale) but were additionally correlated through a shared latent module, ensuring that while all 25 genes would be detected by DE, a tree-based classifier would need only a small number of representatives, leaving the rest with low or zero marginal gain importance; (ii) *Sex-interaction genes* (n = 5), in which disease effects operated in opposite directions across sexes, such that an additive DE model estimating a single disease main effect partially cancels these opposing shifts, yielding weak or non-significant disease coefficients even though the gene is informative when conditioned on sex; (iii) *Heterogeneous disease genes* (n = 5), in which the disease group contained two hidden subtypes with opposing expression shifts relative to controls, such that the overall disease mean was similar to the control mean and DE tests targeting the average disease effect found no signal, whereas tree-based classifiers could exploit the bimodal distribution; (iv) *Threshold-effect genes* (n = 5), in which disease samples included a rare (~20%) high-expression tail balanced by more common mildly reduced expression, yielding little net mean shift but enabling tree models to learn a threshold rule that an additive mean-shift test does not target; (v) *Distribution-shift genes* (n = 5), in which disease samples exhibited increased variance relative to

controls with no change in mean expression, providing classification signal through altered tail behavior without producing a detectable mean shift; (vi) *Age-only genes* (n = 10), which varied with age but not disease status and served as negative controls; and (vii) *Noise genes* (n = 250), which had no disease, age, or sex effects and provided a realistic high-dimensional background.

Differential expression was performed using PyDESeq2 with an additive design (~ age + sex + disease), and a second model including a sex × disease interaction term was fit to aid interpretation of interaction-driven genes. Feature importance was assessed using a LightGBM gradient-boosted decision tree classifier trained on log2-CPM-transformed expression values together with age and sex as covariates (test AUC = 0.981). Genes were classified as DE-significant at FDR < 0.05.

The simulation recapitulated several modes of DE–ML disagreement. All 25 redundant DE genes were FDR-significant (median FDR = 1.29×10^{-15}), but only 13 of 25 (52%) received nonzero LightGBM gain, and only 6 of 25 (24%) ranked among the top 20 genes by gain importance, demonstrating that correlated genes carrying overlapping disease information are detected individually by DE but are rendered partially redundant in a joint model where only a few representatives are needed. In contrast, none of the heterogeneous disease, threshold-effect, or distribution-shift genes (0/15) reached FDR significance in the additive DE model, yet all 15 received nonzero LightGBM gain and 12 of 15 (80%) ranked among the top 20 genes by importance. The heterogeneous disease genes were notable: all five ranked in the top 20 by gain (median gain = 45.99) despite uniformly non-significant FDR values (median FDR = 0.89), illustrating how hidden disease subtypes with canceling mean effects can produce genes that are invisible to DE but highly informative for classification. Similarly, threshold-effect genes achieved high gain importance (median gain = 146.92) through nonlinear decision rules that

exploit rare high-expression tails rather than average shifts, and distribution-shift genes (median gain = 34.35) provided classification signal through altered variance without any change in mean expression. Sex-interaction genes showed an intermediate pattern: 0 of 5 were FDR-significant in the additive model (median additive FDR = 0.71), but the interaction term was highly significant (median interaction $p = 4.17 \times 10^{-5}$), confirming that the signal exists but is not captured by an additive design; two of five received nonzero LightGBM gain, reflecting the classifier's ability to implicitly model interactions through sequential splits. Noise genes behaved as expected: only 2 of 250 (0.8%) reached nominal FDR significance, and only 2 (0.8%) appeared in the top 20 by gain, confirming low false-positive rates for both analyses.

These results demonstrate that disagreement between DE significance and ML feature importance arises from fundamental differences in the statistical questions posed by each analysis. DE asks whether a single gene shows a covariate-adjusted mean shift between disease groups; ML asks whether a gene contributes incremental predictive information within a joint, nonlinear, redundancy-aware model. Five concrete mechanisms produce divergence: (1) redundancy among correlated transcripts, where DE detects each gene individually but a tree ensemble needs only a few representatives; (2) gene × covariate interactions not modeled by additive DE designs; (3) disease subgroup heterogeneity that cancels average effects but produces informative distributional structure; (4) nonlinear threshold effects exploitable by decision trees but invisible to mean-shift tests; and (5) distributional changes (e.g., increased variance) without shifts in central tendency. The observation that 3.9–24.6% of PEGs overlap with DEGs across cell types in our empirical data is therefore consistent with the expected behavior of these two complementary analytical frameworks. Full simulation code is available at

https://github.com/AdiVM/TriSCOPE_RNASeq_Alzheimers_Analysis/tree/main/rnaseq-de-fi-mismatch-main.

Supplementary Discussion

Our results firstly reveal a consistent increase in predictive power as the resolution of analysis shifts from a global to a subcluster-specific lens. While baseline models using only demographics and APOE genotype performed just above chance, the integration of cell-type-specific gene expression significantly boosted performance, with models for populations like Microglia and Oligodendrocytes achieving mean AUCs of approximately 0.65. This performance gradient culminated at the subcluster level, where TriSCOPE captured highly localized disease signals; specifically, classification accuracy reached as high as 0.77. This staircase of predictive performance underscores that AD vulnerability is not a monolithic state but is encoded within discrete transcriptional sub-compartments that are obscured when analyzed at a lower resolution. However, we maintained our primary focus at the broad cell-type resolution to ensure sufficient statistical power for our genetic colocalization and cross-cohort replication analyses, using subcluster-level findings to provide a higher-resolution 'sanity check' on the specific cellular niches where these programs are most active.

We noted that several of the top predictors we identified—such as *LINGO1*, *RASGEF1B*, and *CRYAB*—have either been directly investigated or are biologically tractable drug targets. For example, *LINGO1* inhibitors were previously tested in phase II multiple sclerosis trials as potential remyelination-promoting agents [30,31]. Preclinical work has also shown that hippocampal

overexpression of *Lingo1* improves cognitive function in a mouse model of AD, with knockdown showing the opposite effect [30,31]. Similarly, *RASGEF1B* has been implicated in inflammatory signaling, and its regulation of Intercellular Adhesion Molecule 1 suggests that modulating this axis may influence neuroinflammatory cascades in AD [32]. Additionally, *CRYAB* protein has previously been identified as a myelin binding partner and linked to increased phagocytosis of the myelin sheath [33]. Together, these results highlight genetically anchored, cell-type–specific molecular programs that may guide targeted therapeutic development in Alzheimer's disease, and demonstrate how our pipeline can systematically nominate such high-confidence targets.

Why Machine Learning?

Machine learning is used in this study not as a prognostic tool, but as a variance-controlling and hypothesis-sharpening instrument for gene prioritization in high-dimensional single-nucleus RNA-seq data. Our predictive models are explicitly designed to identify a minimal, multivariately informative subset of genes whose joint expression patterns distinguish Alzheimer's disease (AD) from controls conditional on known demographic and genetic risk factors, rather than to maximize clinical prediction accuracy.

Single-nucleus RNA-seq data are characterized by extreme dimensionality, strong correlation structure among genes, and substantial within-donor variability. As a result, traditional univariate differential expression (DE) analyses typically identify hundreds to thousands of marginally associated genes per cell type (for example, >1,000 DEGs in excitatory neurons), many of which reflect correlated or redundant signals rather than distinct disease-relevant programs. While donor-aware mixed-effects models appropriately regulate statistical significance, they do not

resolve redundancy among correlated transcripts or distinguish which genes remain informative when modeled jointly.

In contrast, the supervised learning framework used in TriSCOPE introduces implicit regularization and competition among correlated features, favoring genes that contribute independent predictive information beyond other transcripts and covariates. Empirically, this results in a substantial compression of the candidate space: across cell types, only 3.9–24.6% of ML-prioritized predictors overlap with DEGs, yielding a focused core of 162 predictive genes in excitatory neurons compared to 1,066 DEGs identified by donor-aware mixed-effects models. Importantly, predictor rank shows only weak correlation with $\log_2$ fold change (Spearman $\rho \approx$ 0.05–0.20), indicating that ML is not simply rediscovering the largest-effect DEGs, but instead prioritizing genes that retain information content when thousands of correlated transcripts are modeled simultaneously.

To further control variance arising from donor sampling, all predictive analyses were repeated across five independent donor-held-out train–test splits, and genes were prioritized based on their consistency across splits rather than performance in any single partition. This design ensures that ML-selected predictors reflect stable, donor-generalizable signal rather than split-specific artifacts, reinforcing the role of supervised learning as a variance-control mechanism rather than a performance-optimized classifier.

ML as conditional hypothesis sharpening for genetics

A second role of machine learning in TriSCOPE is to enable conditional hypothesis testing for genetic anchoring. We perform GWAS enrichment and GWAS–eQTL colocalization analyses within the ML-prioritized gene set, which includes genes that are multivariately informative for

disease classification but may lack significant marginal expression differences and would therefore be excluded from DEG-based genetic analyses.

Although only ~1–2% of predictive genes show strong colocalization evidence (PP.H4 ≥ 0.8), this proportion reflects enrichment within a pre-selected, multivariately informative gene set rather than genome-wide colocalization rates. Critically, several genes with weak or absent differential expression—such as ARL17B, USP6NL, and EGFR—emerge as genetically anchored only after ML prioritization. These genes would not have been highlighted by marginal testing alone, demonstrating that the predictive framework identifies regulatory candidates that are conditionally informative given the broader transcriptional context. In this sense, machine learning functions as a hypothesis-sharpening step that defines a biologically constrained search space for downstream genetic validation.

Importantly, we explicitly emphasize that predictive performance is modest (cell-type AUCs approximately 0.62–0.70) and not intended for clinical deployment. Instead, the value of the ML framework in TriSCOPE lies in its ability to identify reproducible, cell-type-specific transcriptional programs that (i) generalize across donors, (ii) diverge between clinical and neuropathological definitions of AD, and (iii) exhibit convergent support from differential expression, disease-associated pseudotime progression, and genetic colocalization—rather than in forecasting disease status.

Translational Implications

Our findings also have translational implications for therapeutic development. For example, across the three modeling arms of TriSCOPE, we consistently identified microglia-specific neuroinflammatory programs involving *IFI44L, TNFRSF1B*, *SRGN*,

PLXDC2, and *CD81.* Interestingly, several of these genes have previously been identified in a variety of cancers, suggesting a potential shared inflammatory response and opportunities for mechanistic insight or therapeutic repurposing from oncology-focused immune-modulatory strategies [34,35]. Across cell types we identified several genes involved in protein homeostasis and glycosylation. For instance, we identified *HSPA1A* in Microglia and *CRYAB* in Oligodendrocytes, which are both involved in preventing protein clumping in response to cellular stress; we hypothesize that these gene products will play a role in protein aggregation in AD [11]. We also identified genes involved in glycosylation such as *PFKFB3*, *SGCZ*, and *LAMP1* across cell types. These genes may present promising targets for further mechanistic investigation – across cell types – given that N-Glycosylation has been linked to tau hyperphosphorylation in AD [36]. Further study should evaluate if glycosylation is a cell-type specific process in the trajectory to hyperphosphorylation and eventual neuronal death. Consistent with this translational potential, systematic annotation against DrugBank revealed that several high-confidence TriSCOPE-prioritized genes are already linked to existing pharmacologic compounds, highlighting opportunities for targeted perturbation and therapeutic repurposing.

Proposed Translational Target List and Validation Assays

For ARL17B, validation can be performed by CRISPR knockout or overexpression in human iPSC-derived neurons followed by established APP trafficking and processing assays, including ELISA quantification of secreted Aβ and immunoblotting for intracellular APP C-terminal fragments and phospho-tau, directly testing whether altered vesicle trafficking modulates AD-relevant protein handling. For RASGEF1B, neuronal relevance can be evaluated by siRNA or CRISPR perturbation in iPSC-derived excitatory neurons, followed by Ras–ERK pathway activation assays (pERK immunoblotting), dendritic spine density quantification, and

synaptic activity measurements, assays routinely used to assess Ras-dependent neuronal vulnerability in neurodegenerative contexts.

For CRYAB, tractable validation consists of gain- or loss-of-function in OPC–neuron co-culture myelination assays, with quantification of myelin basic protein (MBP) expression and internode formation, complemented by cuprizone or AD mouse models to assess white-matter integrity [37]. LINGO1 is directly actionable using existing antagonistic antibodies; validation can be performed by LINGO1 blockade in APP/PS1 mice or OPC differentiation assays, measuring remyelination and synaptic preservation [38]. For MT3 (metallothionein-3), functional validation can be achieved by neuronal overexpression or knockdown followed by oxidative stress and metal-toxicity assays (e.g., hydrogen peroxide or zinc exposure), with readouts including neuronal survival and reactive oxygen species accumulation, leveraging established paradigms demonstrating MT3-dependent modulation of neuronal vulnerability [39].

For TNFRSF1B (TNFR2), validation can be performed using selective TNFR2 agonists or microglia-specific genetic deletion in AD mouse models, followed by assessment of neuroinflammation, neuronal survival, and amyloid/tau burden, directly testing the neuroprotective TNF signaling axis [40]. For IFI44L, validation consists of inducing type-I interferon signaling in iPSC-derived microglia (e.g., IFN-β treatment) and suppressing the pathway using IFNAR-blocking antibodies or JAK inhibitors, with readouts including Aβ phagocytosis and inflammatory cytokine production.

Supplementary References

1. Stornetta, R. L. & Zhu, J. J. Ras and Rap signaling in synaptic plasticity and mental disorders. *Neuroscientist* **17**, 54–78 (2011).
2. Shankar, G. M. & Walsh, D. M. Alzheimer's disease: synaptic dysfunction and Abeta. *Mol. Neurodegener.* **4**, 48 (2009).
3. Fernandez-Enright, F. & Andrews, J. L. Lingo-1: a novel target in therapy for Alzheimer's disease? *Neural Regen. Res.* **11**, 88–89 (2016).
4. Nishimoto, I. *et al.* Alzheimer amyloid protein precursor complexes with brain GTP-binding protein G(o). *Nature* **362**, 75–79 (1993).
5. Nielsen, E., Cheung, A. Y. & Ueda, T. The regulatory RAB and ARF GTPases for vesicular trafficking. *Plant Physiol.* **147**, 1516–1526 (2008).
6. Zhang, X., Huang, T. Y., Yancey, J., Luo, H. & Zhang, Y.-W. Role of Rab GTPases in Alzheimer's disease. *ACS Chem. Neurosci.* **10**, 828–838 (2019).
7. Thiankhaw, K., Chattipakorn, K., Chattipakorn, S. C. & Chattipakorn, N. Roles of humanin and derivatives on the pathology of neurodegenerative diseases and cognition. *Biochim. Biophys. Acta Gen. Subj.* **1866**, 130097 (2022).
8. Wei, B. *et al.* GR/Ahi1 regulates WDR68-DYRK1A binding and mediates cognitive impairment in prenatally stressed offspring. *Cell. Mol. Life Sci.* **81**, 20 (2024).
9. Jin, M. *et al.* Type-I-interferon signaling drives microglial dysfunction and senescence in human iPSC models of Down syndrome and Alzheimer's disease. *Cell Stem Cell* **29**, 1135–1153.e8 (2022).
10. Qian, Y. *et al.* SRGN amplifies microglia-mediated neuroinflammation and exacerbates ischemic brain injury. *J. Neuroinflammation* **21**, 35 (2024).
11. Dong, Y. *et al.* HSPA1A, HSPA2, and HSPA8 are potential molecular biomarkers for prognosis among HSP70 family in Alzheimer's disease. *Dis. Markers* **2022**, 9480398 (2022).

12. Muraleva, N. A., Kolosova, N. G. & Stefanova, N. A. p38 MAPK-dependent alphaB-crystallin phosphorylation in Alzheimer's disease-like pathology in OXYS rats. *Exp. Gerontol.* **119**, 45–52 (2019).

13. Becerra-Hernández, L. V., Escobar-Betancourt, M. I., Pimienta-Jiménez, H. J. & Buriticá, E. Crystallin alpha-B Overexpression as a possible marker of reactive astrogliosis in human cerebral contusions. *Front. Cell. Neurosci.* **16**, 838551 (2022).

14. Rudenko, L. K. *et al.* Intraneuronal tau misfolding induced by extracellular amyloid-β oligomers. *J. Alzheimers. Dis.* **71**, 1125–1138 (2019).

15. Hwang, S.-M. *et al.* Inflammation-induced Id2 promotes plasticity in regulatory T cells. *Nat. Commun.* **9**, 4736 (2018).

16. Kim, K. Y., Shin, K. Y. & Chang, K.-A. GFAP as a potential biomarker for Alzheimer's disease: A systematic review and meta-analysis. *Cells* **12**, 1309 (2023).

17. Mathys, H. *et al.* Single-cell transcriptomic analysis of Alzheimer's disease. *Nature* **570**, 332–337 (2019).

18. Mou, Y., Zhang, L., Liu, Z. & Song, X. Abundant expression of ferroptosis-related SAT1 is related to unfavorable outcome and immune cell infiltration in low-grade glioma. *BMC Cancer* **22**, 215 (2022).

19. Leng, F. & Edison, P. Neuroinflammation and microglial activation in Alzheimer disease: where do we go from here? *Nat. Rev. Neurol.* **17**, 157–172 (2021).

20. Kedia, S. & Simons, M. Oligodendrocytes in Alzheimer's disease pathophysiology. *Nat. Neurosci.* **28**, 446–456 (2025).

21. Song, L. *et al.* Impaired type I interferon signaling activity implicated in the peripheral blood transcriptome of preclinical Alzheimer's disease. *EBioMedicine* **82**, 104175 (2022).

22. Morabito, S. *et al.* Single-nucleus chromatin accessibility and transcriptomic characterization of Alzheimer's disease. *Nat. Genet.* **53**, 1143–1155 (2021).

23. Sadick, J. S. *et al.* Astrocytes and oligodendrocytes undergo subtype-specific transcriptional changes in Alzheimer's disease. *Neuron* **110**, 1788–1805.e10 (2022).

24. Su, Y. *et al.* A single-cell transcriptome atlas of glial diversity in the human hippocampus across the postnatal lifespan. *Cell Stem Cell* **29**, 1594–1610.e8 (2022).

25. Grubman, A. *et al.* A single-cell atlas of entorhinal cortex from individuals with Alzheimer's disease reveals cell-type-specific gene expression regulation. *Nat. Neurosci.* **22**, 2087–2097 (2019).

26. Foster, E. M., Dangla-Valls, A., Lovestone, S., Ribe, E. M. & Buckley, N. J. Clusterin in Alzheimer's disease: Mechanisms, genetics, and lessons from other pathologies. *Front. Neurosci.* **13**, 164 (2019).

27. GTEx Consortium. The GTEx Consortium atlas of genetic regulatory effects across human tissues. *Science* **369**, 1318–1330 (2020).

28. Fitz, N. F. *et al.* ABCA1 deficiency affects basal cognitive deficits and dendritic density in mice. *J. Alzheimers. Dis.* **56**, 1075–1085 (2017).

29. Rahman, M. M. *et al.* Emerging role of neuron-Glia in neurological disorders: At a glance. *Oxid. Med. Cell. Longev.* **2022**, 3201644 (2022).

30. MS Trust. Opicinumab (anti-LINGO-1). *MS Trust* https://mstrust.org.uk/a-z/opicinumab-anti-lingo-1-biib033 (2015).

31. He, Q. *et al.* The role of LINGO-1 in regulating CB1R/TrkB signalling and GABAergic interneurons in Alzheimer's disease pathogenesis. *Exp. Neurol.* **391**, 115319 (2025).

32. Ng, W. L. *et al.* Inducible RasGEF1B circular RNA is a positive regulator of ICAM-1 in the TLR4/LPS pathway. *RNA Biol.* **13**, 861–871 (2016).

33. Dulle, J. E. & Fort, P. E. Crystallins and neuroinflammation: The glial side of the story. *Biochim. Biophys. Acta* **1860**, 278–286 (2016).

34. Zhang, C. *et al.* Secreted PTEN binds PLXDC2 on macrophages to drive antitumor immunity and tumor suppression. *Dev. Cell* **59**, 3072–3088.e8 (2024).

35. Mohammed, M. A. *et al.* USP6NL knockdown suppresses colorectal cancer progression by inducing CASP9-Mediated apoptosis and disrupting FOXC2/SNAI1-Driven EMT and angiogenesis. *Funct. Integr. Genomics* **25**, 153 (2025).

36. Zhao, J. & Lang, M. New insight into protein glycosylation in the development of Alzheimer's

disease. *Cell Death Discov.* **9**, 314 (2023).

37. Ousman, S. S. *et al.* Protective and therapeutic role for alphaB-crystallin in autoimmune demyelination. *Nature* **448**, 474–479 (2007).

38. Mi, S. *et al.* LINGO-1 negatively regulates myelination by oligodendrocytes. *Nat. Neurosci.* **8**, 745–751 (2005).

39. Hashimoto, K., Hayashi, Y., Watabe, K., Inuzuka, T. & Hozumi, I. Metallothionein-III prevents neuronal death and prolongs life span in amyotrophic lateral sclerosis model mice. *Neuroscience* **189**, 293–298 (2011).

40. Hübschmann, V., Korkut-Demirbaş, M. & Siegert, S. Assessing human iPSC-derived microglia identity and function by immunostaining, phagocytosis, calcium activity, and inflammation assay. *STAR Protoc.* **3**, 101866 (2022).
