# Supplementary material for: Single-cell machine learning uncovers genetically anchored, cell-type specific programs of Alzheimer’s disease
Source: Nat Commun. 2026 Nov 9;17:11124. doi: 10.1038/s41467-026-77651-5 (PMC13748584; doi:10.1038/s41467-026-77651-5)
Supplement: Supplementary file 2 [file 41467_2026_77651_MOESM27_ESM.pdf]

nature portfolio

Corresponding author(s): Chirag J. Patel

Last updated by author(s): Aug 6, 2026

Reporting Summary

Nature Portfolio wishes to improve the reproducibility of the work that we publish. This form provides structure for consistency and transparency in reporting. For further information on Nature Portfolio policies, see our Editorial Policies and the Editorial Policy Checklist.

Statistics

For all statistical analyses, confirm that the following items are present in the figure legend, table legend, main text, or Methods section.

n/a	Confirmed	
☐	☒	The exact sample size (*n*) for each experimental group/condition, given as a discrete number and unit of measurement
☐	☒	A statement on whether measurements were taken from distinct samples or whether the same sample was measured repeatedly
☐	☒	The statistical test(s) used AND whether they are one- or two-sided<br>*Only common tests should be described solely by name; describe more complex techniques in the Methods section.*
☐	☒	A description of all covariates tested
☐	☒	A description of any assumptions or corrections, such as tests of normality and adjustment for multiple comparisons
☐	☒	A full description of the statistical parameters including central tendency (e.g. means) or other basic estimates (e.g. regression coefficient) AND variation (e.g. standard deviation) or associated estimates of uncertainty (e.g. confidence intervals)
☐	☒	For null hypothesis testing, the test statistic (e.g. *F*, *t*, *r*) with confidence intervals, effect sizes, degrees of freedom and *P* value noted<br>*Give P values as exact values whenever suitable.*
☐	☒	For Bayesian analysis, information on the choice of priors and Markov chain Monte Carlo settings
☐	☒	For hierarchical and complex designs, identification of the appropriate level for tests and full reporting of outcomes
☐	☒	Estimates of effect sizes (e.g. Cohen's *d*, Pearson's *r*), indicating how they were calculated

Our web collection on statistics for biologists contains articles on many of the points above.

Software and code

Policy information about availability of computer code

Data collection	No software was used for data collection. This study is a secondary computational analysis of previously published datasets downloaded from public repositories as described in the Data Availability statement.
Data analysis	Data analysis: Predictive modelling, pseudotime inference and figure generation were performed in Python v3.9.21 using FLAML v2.2.0, LightGBM v4.5.0, scikit-learn v1.6.1, Scanpy v1.10.3, anndata v0.10.8, GSEApy v1.1.9, SHAP v0.48.0, NumPy v2.0.0, pandas v2.2.2, SciPy v1.13.1, statsmodels v0.14.4, matplotlib v3.9.4, seaborn v0.13.2, upsetplot v0.9.0, joblib v1.5.1 and h5py v3.14.0.<br><br>Differential expression analyses were performed in R v4.4.2 using lme4 v1.1.38, glmmTMB v1.1.13, RUVSeq v1.40.0, edgeR v4.4.2, limma v3.62.2, dplyr v1.1.4, data.table v1.17.8, arrow v22.0.0, Seurat v5.4.0 and biomaRt v2.62.1.<br><br>GWAS–eQTL colocalization was performed in R v4.5.1 using coloc v5.2.3.<br><br>Custom code developed for this study is available at https://github.com/AdiVM/TriSCOPE_RNASeq_Alzheimers_Analysis and archived at https://doi.org/10.5281/zenodo.21793738 under the MIT License.

For manuscripts utilizing custom algorithms or software that are central to the research but not yet described in published literature, software must be made available to editors and reviewers. We strongly encourage code deposition in a community repository (e.g. GitHub). See the Nature Portfolio guidelines for submitting code & software for further information.

Data

Policy information about availability of data

All manuscripts must include a data availability statement. This statement should provide the following information, where applicable:

- Accession codes, unique identifiers, or web links for publicly available datasets
- A description of any restrictions on data availability
- For clinical datasets or third party data, please ensure that the statement adheres to our policy

The data used in this study are available as follows.
1. The Mathys-48 dataset is available on Synapse under accession syn18485175 [https://www.synapse.org/#!Synapse:syn18485175]. ROSMAP metadata is also accessible via Synapse [https://www.synapse.org/#!Synapse:syn3157322]. This same snRNA-seq dataset with further quality control measures applied to it is publicly available on Synapse [https://doi.org/10.7303/syn51758062.1].
2. The Lau-21 dataset is deposited on the NCBI Gene Expression Omnibus under accession GSE157827 [https://www.ncbi.nlm.nih.gov/geo/query/acc.cgi?acc=GSE157827].
3. The ROSMAP-48b dataset was generated as part of the MIT ROSMAP Single-Nucleus Multiomics Study, and is available through Synapse under accession syn52293442 [https://www.synapse.org/Synapse:syn52293442].
4. The ROSMAP-427 dataset is available through Synapse under accession syn52293417 [https://www.synapse.org/Synapse:syn52293417].
5. Single-nucleus RNA-seq data from the Seattle Alzheimer's Disease Brain Cell Atlas (SEA-AD) are available through the Allen Institute Open Data Registry on AWS [https://sea-ad-single-cell-profiling.s3.amazonaws.com/index.html] and through controlled access on the AD Knowledge Portal on Synapse under accession syn26223298 [https://www.synapse.org/Synapse:syn26223298]. Donor, library, and cell-level metadata are available at [https://portal.brain-map.org/explore/seattle-alzheimers-disease].
6. Cis-eQTL data: GTEx v10 significant cis-eQTL variant–gene pairs for all brain tissues were obtained from the GTEx Portal [https://gtexportal.org/home/] on 07/26/25.
7. Alzheimer's disease GWAS summary statistics: GWAS summary statistics were obtained from the NHGRI-EBI GWAS Catalog [https://www.ebi.ac.uk/gwas/efotraits/MONDO_0004975].
8. Processed differential expression results were obtained from four previously published studies: Morabito et al.63 Supplementary Data 1C [https://doi.org/10.1038/s41588-021-00894-z], Sadick et al.64 Tables S4 and S6 [https://doi.org/10.1016/j.neuron.2022.03.008], Su et al. 65 Table S5A [https://doi.org/10.1016/j.stem.2022.09.010], and Grubman et al.66 Supplementary Data 2 [https://doi.org/10.1038/s41593-019-0539-4]. Each file is available from the supplementary information of the corresponding publication.
Access to the ROSMAP-derived and SEA-AD datasets is controlled to protect participant privacy, as these are classified as sensitive NIH controlled-access data. Access requires an executed Data Use Agreement and registration with the data provider; conditions of access, required approvals and expected response times are maintained by the providers at [https://adknowledgeportal.synapse.org] and [https://www.radc.rush.edu/docs/omics/overview.htm]. We did not generate these data and had no role in setting the conditions of access. Source data are provided with this paper.

Research involving human participants, their data, or biological material

Policy information about studies with human participants or human data. See also policy information about sex, gender (identity/presentation), and sexual orientation and race, ethnicity and racism.

Reporting on sex and gender	Sex, as a biological attribute, was considered in the study design. It was included as a demographic feature in the combined predictive models, and model interpretation was additionally stratified by sex (Supplementary Fig. 4). The study analysed 627 donors: 309 male and 318 female. Per cohort: Mathys-48 (24 male, 24 female), Lau-21 (14 male, 7 female), ROSMAP-48b (24 male, 24 female), ROSMAP-427 (212 male, 215 female), SEA-AD (35 male, 48 female). Findings are not restricted to one sex; results are reported for the combined cohort with sex-stratified model interpretation provided alongside. Sex was recorded by the contributing cohorts during their original data collection; this study is a secondary analysis and neither collected nor assigned this variable, so the method of ascertainment is that described in the original cohort publications. Gender was not available in the metadata released for the contributing cohorts and therefore was not analyzed. Disaggregated donor-level sex is provided in Supplementary Data 15 and in the Source Data file.
Reporting on race, ethnicity, or other socially relevant groupings	No race, ethnicity or other socially constructed categorization variables were used in this study. These variables were not included as model features or covariates, were not used to stratify or define any analysis, and are not reported in Supplementary Data 15; consequently no analyses were adjusted for them. The donor-level covariates used were age at death, sex, years of formal education completed, and APOE genotype. Years of education was used directly as a demographic covariate and was not used as a proxy for any other construct. The ancestry composition of the contributing cohorts is described in their original publications and constrains the generalizability of these findings.
Population characteristics	Participants were deceased adult brain donors from six previously published single-nucleus RNA-seq datasets, totalling 627 donors and approximately 4.2 million nuclei. Age at death spans the 60–62 to 90+ year bands; 309 donors were male and 318 female. Covariate-relevant characteristics available across cohorts are age at death, sex, APOE genotype, years of formal education completed, post-mortem interval, clinical diagnosis and CERAD neuritic plaque score, all reported per donor in Supplementary Data 15. Disease status was defined either by clinical diagnosis (Alzheimer's dementia versus no cognitive impairment) or by neuropathological burden (CERAD score), depending on the analysis. No treatment categories apply, as all data are post-mortem.
Recruitment	No participants were recruited by the present study; all data were obtained from previously published cohorts. Recruitment procedures and inclusion criteria are described in the original cohort publications. Because the contributing cohorts were assembled as longitudinal ageing and dementia studies with brain donation at enrolment, donors are older than the general population, enriched for cognitive impairment, and self-selected through willingness to consent to autopsy. These features limit generalizability to the wider population but are unlikely to bias the within-cohort comparisons reported here, which contrast donors drawn from the same recruitment stream.

Ethics oversight	The ROSMAP-derived cohorts (Mathys-48, ROSMAP-48b, ROSMAP-427) were approved by the Institutional Review Board of Rush University Medical Center. The Lau-21 and Seattle Alzheimer's Disease Brain Cell Atlas cohorts were approved by the ethics bodies identified in their original publications. The present analysis used only de-identified previously published data, did not constitute human subjects research, and did not require institutional review board approval.

Note that full information on the approval of the study protocol must also be provided in the manuscript.

Field-specific reporting

Please select the one below that is the best fit for your research. If you are not sure, read the appropriate sections before making your selection.

☒ Life sciences ☐ Behavioural & social sciences ☐ Ecological, evolutionary & environmental sciences

For a reference copy of the document with all sections, see nature.com/documents/nr-reporting-summary-flat.pdf

Life sciences study design

All studies must disclose on these points even when the disclosure is negative.

Sample size	No sample-size calculation was performed. This study is a secondary analysis of previously published datasets, so sample size was not determined by the investigators: all donors and nuclei available in each dataset were included, totalling 627 donors and approximately 4.2 million nuclei. These sizes are sufficient for the analyses performed because each of the six major brain cell types is represented by tens of thousands to hundreds of thousands of nuclei drawn from hundreds of donors, which supports donor-level mixed-effects differential expression and donor-held-out predictive modelling at that resolution.
Data exclusions	Nuclei missing essential metadata were excluded: 941 nuclei lacking post-mortem interval values in the Murphy et al. re-analysis, and 13 ROSMAP-427 donors absent from the clinical metadata file were excluded from analyses requiring clinical diagnosis or CERAD score. Subclusters containing fewer than 50 cells were not analysed at the subcluster level. All exclusion criteria were pre-established on the basis of metadata completeness and minimum cell count. No other data were excluded.
Replication	Predictive models were run across five independent donor-held-out cross-validation splits, and pseudotime inference was repeated across multiple random seeds per subcluster; all reported estimates are aggregated across these repeats. Findings were tested for replication in two independent cohorts (Lau-21 and the Seattle Alzheimer's Disease Brain Cell Atlas), in two additional data releases from the ROSMAP cohort, and against published differential expression results from four further studies. Key predictors replicated across cohorts; concordance with external differential expression rankings varied by cell type, and all replication results are reported in full in the manuscript and Supplementary Data.
Randomization	Participants were not allocated into experimental groups by the investigators. Group membership was determined by the clinical diagnosis and neuropathological scores recorded by the original cohort studies. For predictive modelling, donors were randomly partitioned into training and test sets (80/20) at the donor level using a fixed random seed per split, with the split stratified jointly by disease status and sex so that both were balanced across training and test sets. Age at death, sex, years of education and APOE genotype were additionally included as model covariates, and differential expression models included RUV covariates and a donor-level random intercept.
Blinding	Blinding was not relevant to this study. All analyses were computational and applied to previously collected data carrying pre-existing diagnostic labels assigned by the original cohort studies; investigators played no role in assigning disease status, and predictions were generated algorithmically.

Reporting for specific materials, systems and methods

We require information from authors about some types of materials, experimental systems and methods used in many studies. Here, indicate whether each material, system or method listed is relevant to your study. If you are not sure if a list item applies to your research, read the appropriate section before selecting a response.

Materials & experimental systems

n/a	Involved in the study	
☒	☐	Antibodies
☒	☐	Eukaryotic cell lines
☒	☐	Palaeontology and archaeology
☒	☐	Animals and other organisms
☒	☐	Clinical data
☒	☐	Dual use research of concern
☒	☐	Plants

Methods

n/a	Involved in the study	
☒	☐	ChIP-seq
☒	☐	Flow cytometry
☒	☐	MRI-based neuroimaging

Plants

Seed stocks	n/a
Novel plant genotypes	n/a
Authentication	n/a
