# Supplementary material for: Single-cell machine learning uncovers genetically anchored, cell-type specific programs of Alzheimer’s disease
Source: Nat Commun. 2026 Nov 9;17:11124. doi: 10.1038/s41467-026-77651-5 (PMC13748584; doi:10.1038/s41467-026-77651-5)
Supplement: Supplementary file 3 [file 41467_2026_77651_MOESM28_ESM.pdf]

nature portfolio

Peer Review File

Single-cell machine learning uncovers genetically anchored, cell-type specific programs of Alzheimer's disease

Corresponding Author: Professor Chirag Patel

This file contains all reviewer reports in order by version, followed by all author rebuttals in order by version.

Version 0:

Reviewer comments:

Reviewer #1

(Remarks to the Author)
This manuscript by Madduri et al looked into cell type specific molecular programme governing clinical symptoms and the neuropathological burden in AD. The authors used gene expression data from snRNAseq data along with demographics to predict both clinical and neuropathological outcome. I am enthusiastic about the integrated method and the impact of the publication. However, there are several points that needs to be reconsidered before publication.
Major
1) The result section is a bit repetitive with description of individual genes which can be concised to better capture the main contributors.
2) The authors decided to omit the random effect for the snRNAseq based DEG which reduces the confidence in the DEG. Although, the authors tested the pseudobulk method and mentioned that the logFC direction generally agrees but did not use the results indicates that the pvalues were large in pseudobulk. DEG methods are still not consolidated in the field however, omitting random effect inflates the pvalue a lot (see https://www.nature.com/articles/s41467-021-21038-1). A more appropriate DEG method is therefore needed. It was not clear whether the authors re-analysed the data from Mathys et al 2019 or used the processed data from the said publication. While Mathys et al 2019 is one of the pioneering work in the field, the field has advanced in the best practice of the analysis which should be adopted in the work. Specifically, the cellranger version (see the preprint https://pmc.ncbi.nlm.nih.gov/articles/PMC11014608/#S2), the removal of ambient RNA contamination, use of appropriate QC are important. In this context, the authors mentioned the following without specifying the percent mitochondria threshold used.

"We applied additional quality control filters to exclude cells with low gene detection or aberrant mitochondrial read fractions, resulting in a final dataset comprising 17,926 genes and 70,634 high-quality nuclei." Though, I appreciate the authors attempted to reproduce the results using Murphy et al qc criteria. I encourage the authors to report the findings in the result section.
3) There are now much larger well characterised AD datasets available from ROSMAP (i.e. Mathys et al 2024) and Allen Brain Atlas. I encourage the authors to use those datasets for increased power.

Minor:
1. The manuscript file lacks page numbers and line numbers, so it's difficult to point out the concerns page by page.
2. In the TriScope method section, the authors mentioned "Genes with low mean expression or high sparsity were excluded prior to feature selection." Please report how many genes were left after this filtering.
3. Had the authors identified any limitations, they should report that in the limitation section.

(Remarks on code availability)
N/A

Reviewer #2

(Remarks to the Author)
The authors train a predictive model of AD case control status using a single cell data-set from the ROSMAP cohort (the Mathys et al. data) and identify cell type specific predictors of AD status which they validate in an independent data-set (from

Lau et al). They then show that the most predictive features from the cell type expression predictor of AD are enriched in known AD genetic loci. They also apply pseudotime inference to the single cell RNA-seq data and show enrichment for relevant AD pathways (e.g. Amyloid processing). The study is focused on machine learning approaches to explore the relationship between variation in single cell expression and disease diagnosis (particularly with the focus on disease state classification). I have some major concerns with the design and novelty of the study that I've outlined below along with some minor concerns. Overall the paper could use some aggressive editing and streamlining to focus on what is novel about their approach compared to what has already been published on single-cell RNAseq analyses in AD.

Major Concerns
1. Why use such a small training (n=48) and validation data-set (n=21)? And also why not use some more recently published data as well? Both studies they use are more than five years old. There are now publicly available single cell data-sets on hundreds if not thousands of unique participants from various sources derived including SEA-AD (https://doi.org/10.1038/s41593-024-01774-5), additional ROSMAP data (https://doi.org/10.1038/s41588-024-01685-y & https://doi.org/10.1016/j.cell.2023.08.039), MSBB (https://doi.org/10.1038/s41597-023-02507-2), AZSAND (https://doi.org/10.1038/s41467-024-49790-0), and others.
2. They focused primarily on a machine learning approach to identify features of interest. I suspect that the most correlated features with disease state within each cell population will be very similar to the most predictive features in the GBM approach - why isn't there a direct comparison of the ordering of predictive features and the ordering of differentially expressed genes? Apologies if it is there, I looked for it but could not find it.
3. There are snRNA-seq papers showing enrichment for genetic loci in genes that are correlated with disease status from single cell data. Specific examples include APOE (https://doi.org/10.1038/s41593-024-01774-5, https://doi.org/10.1038/s41588-024-01685-y), CLU (https://doi.org/10.1186/s13073-022-01136-5), BIN1 (https://doi.org/10.1038/s41593-019-0539-4) and particularly Mathys et al. 2024 (https://doi.org/10.1038/s41586-024-07606-7) overall. How are their results materially different from these previous observations?
4. The disease pseudotime aspect of the paper is confusing to me - what exactly is it adding to the story that is not already captured in the predictive disease features and differential expressed genes? Why not have a direct comparison between all of their analyses somewhere for the reader to understand what is similar and what is different? Also how is it materially different than previous pseudotime (or pseudotrajectory) observations made in other snRNA-seq AD papers (e.g. from SEA-AD)?
5. The authors use very strong language to describe the genes that they prioritize with their analysis e.g. from the abstract - "our study nominates high-confidence, genetically anchored molecular drivers of AD and prioritizes them for mechanistic investigation and therapeutic targeting in age-related neurodegenerative disease". I am not convinced that the authors have shown anything but some evidence that transcriptional regulation of a given locus is associated with AD status and is dysregulated in AD brains within a certain cell popluation. A great example of this is ARL17B - a more parsimonious possible explanation given what we know about the role of tau pathology in AD may be that MAPT is the causal gene within the locus but MAPT protein expression (or perhaps phopho-tau expression) is co-regulated with ARL17B mRNA expression and so is invisible in the expression data view. The authors should tone down the language with this sort of caveat.

Minor Concerns
1. Did they try other approaches for prediction of disease state than a GBM? Does model selection matter?
2. Did they try to fit a model with multiple cell types to predict disease state? Is a multi cell type model more predictive of disease state?
3. I wasn't able to open the supplementary tables.

(Remarks on code availability)
I did not try to run the code, but I did look through all of the attached jupyter notebooks and the approach looks very reasonable and should work.

Reviewer #3

(Remarks to the Author)

This manuscript introduces TriSCOPE as an integrative framework that combines multivariate machine learning, differential expression, and pseudotime analysis to identify cell-type and subcluster-specific programs associated with clinical and neuropathological Alzheimer's disease, with cross-cohort validation and genetic anchoring to GWAS/eQTL signals. The study is thoughtfully motivated and biologically plausible, recovering known AD features while nominating underexplored candidates. It usefully contrasts clinical vs pathology readouts across cell types. At the same time, important clarifications and additions would strengthen impact and adoptions; specifically clearer articulation and empirical benchmarking of TriSCOPE;s added value over standard pipelines (univariate DE, pseudotime/GLMs or widely used integrative models), broader validation and sensitivity analyses to solidify robustness and reproducibility/usability improvements (open code, tutorials and compute scaling). In short, the work while promising and lose to broadly useful, still requires additional evidence and clearer communication of when and why TriSCOPE should be preferred over "normal analysis". As a prospective used, I still find it not fully clear what unique decision(s) TriSCOPE enables beyond standard DE + pathway + pseudotime and the manuscript should explicitly define the core use-case and demonstrate a measurable gain over baselines on both cohorts.

Major Comments
1. It is strongly advised to the authors to benchmark TriSCOPE against alternative integrative methods (e.g., LASSO/logistic, MOFA+, scVI/scANVI, deep embeddings) and report comparative performance with confidence intervals.
2. It would be also important to expand the validation beyond the PFC by testing cross-region and cross-cohort generalizability (e.g., hippocampus, other public datasets).
3. I suggest the authors should perform sensitivity analyses that explicitly control for donor and technical confounders (PMI/RIN/batch/co-pathologies) and show robustness of features and AUCs under these controls.
4. It would be interesting to see if the pseudotime analysis is affected by circularity due to DE gene anchors. The authors should address circularity in pseudotime by constructing trajectories independent of DE gene selection and validating trajectory direction with orthogonal signals (e.g., amyloid burden per donor, pathology staging).
5. It would be suitable to provide APOE- and sex-stratified models and SHAP summaries to demonstrate genotype- and sex-specific predictor stability and divergence.
6. Although the authors utilize the GWAS/eQTL signal, it might be important to show the integration of genetic evidence more formally by testing colocalization (using tools such as coloc, eCAVIAR) between AD GWAS and brain eQTLs for top predictors to substantiate the “genetically anchored” claim.
7. The authors might clarify translational relevance by nominating a short, ranked target list with mechanism, tractability, and proposed assays (e.g., LINGO1 inhibition, CRYAB modulation in myelination assays) as part of their results.
8. It is also strongly advised to supply a minimal example (subset of cells/genes) demonstrating the entire TriSCOPE workflow including feature selection, AutoML training, grouped CV by donor, SHAP interpretation, DE, pseudotime, so that future users can adopt without re-engineering. Including inputs/outputs for each stage and expected metrics is essential.
9. It would be beneficial to report runtime, peak memory, and hardware for each stage (feature selection, FLAML/LightGBM training, SHAP, DE, pseudotime) as a function of cells × genes for both datasets and include detailed tables in the Supplement.
10. The authors should clearly state typical AUC ranges and variance across splits (CIs), emphasizing that predictive gains are modest at whole-dataset level but stronger at cell type / subcluster resolution; this sets realistic expectations for users deciding when TriSCOPE is beneficial and should be clearly communicated.
11. It is suggested to the authors to provide an explicit negative-control analysis (such as permuted labels, shuffled donor blocks) to bound false-positive discovery rates and report the results at least in Supplement.
12. The manuscript notes convergence issues for mixed-effects models and proceeds without a donor random intercept. Given the hierarchical structure of snRNA-seq (many cells nested within donors), the lack of a random effect risks anti-conservative inference (inflated significance from pseudo-replication). Please explicitly discuss this limitation and its expected impact and add robustness checks demonstrating that key DE findings are not driven by within-donor correlation. Concretely, I recommend one or more of the following.

Minor Comments
- The authors should improve figure readability with a single overview panel that maps top predictors per cell type and indicates GWAS/eQTL support.
- It is essential to report uncertainty on all metrics (AUC CIs, feature importance variability across seeds/splits) and include calibration plots for classifiers.
- The authors might want to compare pathway results across methods (GSEA on predictors vs DEGs vs ORA) and report convergent vs unique terms.
- The authors should discuss limitations of cross-sectional prediction (moderate AUCs, lack of temporality) and outline how longitudinal or matched multi-region sampling could resolve them.

(Remarks on code availability)

Version 1:

Reviewer comments:

Reviewer #1

(Remarks to the Author)
I thank the authors for meticulously revising the manuscript to consider the reviewers' comments.

(Remarks on code availability)
N/A

Reviewer #2

(Remarks to the Author)
While the authors acknowledged some aspects of the feedback from the first round of revisions, I do think there are still a couple areas that could be improved to make it very clear what novel insights into potential new avenues of AD biology the triscope method is illuminating as well as clearly disclosing the potential limitations of the approach. That being said, I particularly appreciated the observations about USP6NL and EGFR as genes that were not differentially expressed but looked very promising when considering the GWAS-eQTL colocalization analysis. This sort of observation is what

potentially sets the authors' analysis apart from previously reported analyses. Are there other examples like this (or even an updated figure) to show this so it's very clear to the reader what the potentially new and biologically relevant observations actually are?

Major Notes:
I have new concerns regarding their feedback to my first comment from the previous review ("Why use such a small training (n=48) and validation data-set (n=21)? And also why not use some more recently published data as well? Both studies they use are more than five years old…."):
First, now they have 3 ROSMAP data-sets, and 1 validation data-set (Lau et al.). I pointed out multiple other data-sets that are not derived from the ROSMAP cohort (e.g. SEA-AD, MSBB, etc…) that are publicly available - why did they only add ROSMAP samples? If the triscope method is truly more robust as the authors claim, shouldn't the results generalize to other large scale cohorts?
Second, maybe I'm confused as to the analyses they performed, but why did they just do a differential expression analysis in the larger ROSMAP cohort (n=427), and not test and report their cell type specific predictive model generalization performance in this data-set as well? Was this due to computational reasons, or something to do with this vague statement "We note that retraining models on each dataset is an important but distinct objective, and we view large-scale cross-study model training as a valuable direction for future work as harmonized, multi-cohort single-cell resources continue to mature."? Why couldn't they just test their Mathys 2019 model prediction performance in the Mathys et al 2023 n=427 data-set (without retraining)? Does that mean they did try it and the results did not generalize? If so, they should report this discrepancy and discuss what this means regarding the quality of the relevant data-sets, as readers should know if there are in fact limitations of particular data-sets or potential concerns regarding model generalizability when considering larger data-sets. If it was a computational limitation they should note this, and perhaps run a sub-sampled analysis of the larger Mathys data-set. Also, whenever they discuss a particular data-set they should cite the paper where the data-set is described so the reader knows which data-set they are referring to. For example, on page 37, lines 724-727: "External validation was performed using an independent snRNA-seq dataset comprising 12 AD patients and 9 controls (totaling 169,496 nuclei across microglia, oligodendrocytes, excitatory neurons, inhibitory neurons, astrocytes)." I assume this is the Lau data-set? Regardless, the authors should make it very clear to the reader which exact data-set is being referred to in each section for all data-sets. It might be helpful to have a main text table describing the data-sets, sample sizes, and which analyses from triscope are performed in each one so the reader can follow along easily.
My second concern is again around the interpretation of the ARL17B result which they still emphasize very strongly in the abstract and later in the manuscript. The authors response to my concern was:
"We have toned down the strong language that we previously used to describe the genes prioritized by our integrative framework, and now emphasize that these genes should be interpreted as high-confidence candidates supported by convergent evidence, rather than definitive causal drivers. Importantly, our claims are grounded not in transcriptional association alone, but in the convergence of four orthogonal signals—multivariate predictive relevance, donor-aware differential expression, pseudotime-based trajectory inference, and conditional GWAS–eQTL colocalization. In this context, loci such as ARL17B are not asserted to be the sole causal effectors; rather, our analyses provide strong evidence that genetic risk at the locus is mediated through transcriptional regulation captured at the single-cell level, even in the presence of alternative mechanistic explanations (e.g., co-regulation with MAPT protein or tau pathology). We therefore frame these genes as genetically anchored molecular candidates that warrant mechanistic follow-up, while explicitly acknowledging that causal resolution will require perturbational and proteomic validation."
Again, I would encourage the authors to be cautious with their interpretation of the results of their analyses. While a conditional GWAS-eQTL analysis combined with donor aware cell type differential expression, feature importance in a cell type aware disease diagnosis predictive model, and cell type specific pseudotime is potentially more powerful than a differential expression/eQTL analysis alone for identifying potential mRNAs/genes of interest in disease, any insights are still fundamentally limited by the nature of the instrument variables that you are measuring and analyzing (e.g. mRNA transcript abundance at a fixed point in time in a post mortem tissue across a heterogenous cohort of cases and controls) with regards to a dynamic multi-cellular multi-scale process such as is happening in neurodegeneration. If the authors really want to strengthen their argument, they may consider layering in additional multi-omic evidence that is orthogonal to mRNA expression for some top candidate novel targets (e.g. proteomics or methylation for their short list of high value targets). Furthermore, as noted in their discussion in the MAPT (ARL17B) locus there is a large scale genomic inversion, so I would tread very carefully with the interpretation of strong eQTL within that locus, because there are unusually high levels of linkage disequilibrium throughout the locus that may be resistant even to careful conditional colocalization analysis.

Minor notes
The whole manuscript could use a thorough read through to improve grammar and readability (an illustrative example here): pg. 47, Line 1048, "We found are pan-cell type predictors of ARL17B, LINGO1, RASGEF1B, and SLC26A3 to be predictive across cell types, and found cell type specificity for PLXDC2 in Microglia, GRID2 in Oligodendrocytes, and GFAP in Astrocytes. We additionally found predictive performance to be likewise similar."

(Remarks on code availability)
I could not access the code at:

https://github.com/AdiVM/TriSCOPE_RNASeq_Alzheimers_Analysis

I got a 404 error.

Reviewer #3

(Remarks to the Author)
All the comments were successfully addressed.

(Remarks on code availability)

Version 2:

Reviewer comments:

Reviewer #2

(Remarks to the Author)
The authors have adequately addressed all of my concerns in the most recent revision of the manuscript.

(Remarks on code availability)

Open Access This Peer Review File is licensed under a Creative Commons Attribution 4.0 International License, which permits use, sharing, adaptation, distribution and reproduction in any medium or format, as long as you give appropriate credit to the original author(s) and the source, provide a link to the Creative Commons license, and indicate if changes were made.
In cases where reviewers are anonymous, credit should be given to 'Anonymous Referee' and the source.
The images or other third party material in this Peer Review File are included in the article's Creative Commons license, unless indicated otherwise in a credit line to the material. If material is not included in the article's Creative Commons license and your intended use is not permitted by statutory regulation or exceeds the permitted use, you will need to obtain permission directly from the copyright holder.
To view a copy of this license, visit https://creativecommons.org/licenses/by/4.0/

Reviewer #1 (Remarks to the Author):

This manuscript by Madduri et al looked into cell type specific molecular programme governing clinical symptoms and the neuropathological burden in AD. The authors used gene expression data from snRNAseq data along with demographics to predict both clinical and neuropathological outcome. I am enthusiastic about the integrated method and the impact of the publication. However, there are several points that needs to be reconsidered before publication.
Major
1) The result section is a bit repetitive with description of individual genes which can be concised to better capture the main contributors.

First, we thank the reviewer for taking the time to read and provide positive feedback on our manuscript. We have streamlined the Results section by removing extended descriptions of individual genes. The revised text now focuses on a concise set of key contributors, with brief references to broader pathways or gene families where appropriate. Detailed gene-level functional descriptions have been moved to the Supplementary Information to improve clarity and readability of the main text.

2) The authors decided to omit the random effect for the snRNAseq based DEG which reduces the confidence in the DEG. Although, the authors tested the pseudobulk method and mentioned that the logFC direction generally agrees but did not use the results indicates that the pvalues were large in pseudobulk. DEG methods are still not consolidated in the field however, omitting random effect inflates the pvalue a lot (see https://www.nature.com/articles/s41467-021-21038-1). A more appropriate DEG method is therefore needed. It was not clear whether the authors re-analysed the data from Mathys et al 2019 or used the processed data from the said publication. While Mathys et al 2019 is one of the pioneering work in the field, the field has advanced in the best practice of the analysis which should be adopted in the work. Specifically, the cellranger version (see the preprint https://pmc.ncbi.nlm.nih.gov/articles/PMC11014608/#S2), the removal of ambient RNA contamination, use of appropriate QC are important. In this context, the authors mentioned the following without specifying the percent mitochondria threshold used.

We have replaced our previous Differential Expression method with a Random Effects Model for all primary analyses reported in the main text. This method is now described in detail in our methods section. All sections of the paper reporting differential expression results have now been modified to report results from the Random Effects model, with the previous analysis (which omitted the random effect) moved to the supplement. We further perform a concordance analysis between the two approaches and report strong concordance in the supplement. We further performed an extended differential expression analysis of 427 donors using the random-effects method.

We used the processed version of the Mathys et al matrix. As such we now report the percent mitochondria filtering approach they used in their analysis. Specifically, in the original Mathys et al. 2019 processing pipeline, mitochondrial filtering was performed using a data-driven approach rather than a fixed percentage threshold. Specifically, a mitochondrial ratio (mitochondrial read fraction normalized by the number of detected genes) was computed per nucleus using the scater framework,

and k-means clustering (k = 2) was applied to separate nuclei into low- and high-mitochondrial groups, with the high-mitochondrial cluster excluded from downstream analyses. As a result, the effective mitochondrial cutoff was determined empirically by the clustering outcome rather than by a predefined percentage value.

To account for potential issues in this processing approach, we reran our predictive models with the Murphy et al quality control measures, which we now report in the manuscript. In brief, the enhanced quality control measures involve: Murphy et al. applied updated quality-control procedures to the Mathys dataset, including explicit filtering of empty droplets, doublets, and nuclei with low complexity or high mitochondrial content, using quantitative cutoffs informed by contemporary best-practice guidelines. They also performed donor-level integration to mitigate batch effects across samples. These steps resulted in a smaller, higher-quality cell set for downstream analyses. We report the results of this analysis in response to the next question.

"We applied additional quality control filters to exclude cells with low gene detection or aberrant mitochondrial read fractions, resulting in a final dataset comprising 17,926 genes and 70,634 high-quality nuclei." Though, I appreciate the authors attempted to reproduce the results using Murphy et al qc criteria. I encourage the authors to report the findings in the result section.

We have included our analysis of Murphy et al data in the results, and performed a correlation analysis between the Murphy et al data and our findings on the Mathys et al 2019 data. We found concordance between predictive genes between the two datasets, which are reported in our supplementary results section titled: **Robustness of Predictive Gene Prioritization to Updated snRNA-seq Quality Control**, and we report the concordance between the two analyses in Supplementary Table 18. Notably, TriSCOPE identified predictors exhibit relatively strong concordance in this case, where differential expression analysis results differ significantly depending on methodology and approach.

Our supplementary results section "**Robustness of Predictive Gene Prioritization to Updated snRNA-seq Quality Control**" is as follows:
Mathys et al. (2019) conducted the first large-scale single-nucleus RNA-sequencing (snRNA-seq) study of Alzheimer's disease (AD), enabling the discovery of cell-type–specific transcriptional changes that are not observable in bulk RNA-seq data. Subsequent methodological work by Murphy et al., however, highlighted important sensitivities in snRNA-seq differential expression (DE) analyses to data processing, quality control, and statistical modeling choices, demonstrating that alternative, best-practice analytical pipelines can substantially alter the set of genes identified as differentially expressed. In brief, Murphy et al. applied updated quality-control procedures to the Mathys dataset, including explicit filtering of empty droplets, doublets, and nuclei with low complexity or high mitochondrial content, using quantitative cutoffs informed by contemporary best-practice guidelines. They also performed donor-level integration to mitigate batch effects across samples. These steps resulted in a smaller, higher-quality cell set for downstream analyses. These findings underscore that DEG discovery in single-cell data can be highly dependent on upstream analytical assumptions. Given this critique, we sought to explicitly test whether the predictive gene prioritization produced by TriSCOPE is sensitive to these upstream processing and QC decisions, or whether it identifies a more stable subset of disease-relevant genes that persists even under substantially stricter filtering and revised analytical assumptions. To this end, we reran

the full TriSCOPE predictive modeling pipeline using the Murphy/Skene–processed snRNA-seq data in place of the original Mathys et al. processed data, while holding all downstream modeling choices fixed (including donor-aware cross-validation, feature selection, and gradient-boosted decision tree classifiers). We then compared the resulting predictive gene rankings between the two pipelines on a cell-type–specific basis, focusing on both (i) rank concordance across all shared genes and (ii) overlap among consistently selected predictors. For each major brain cell type (Astrocytes, Microglia, Inhibitory Neurons, Oligodendrocytes, OPCs, and Excitatory Neurons), we computed the Spearman rank correlation between mean normalized feature importance scores derived from the original Mathys-based pipeline and the Murphy/Skene-based pipeline, across all genes shared between the two analyses. Despite substantial differences in upstream QC and DEG stringency, predictive gene rankings were significantly correlated across all cell types. Rank concordance was strongest in glial populations, with Spearman correlations of $\rho = 0.442$ in Microglia, $\rho = 0.440$ in Oligodendrocytes, and $\rho = 0.458$ in OPCs (all $p \ll 10^{-40}$). Excitatory neurons also showed robust concordance ($\rho = 0.345$, $p \approx 10^{-210}$), while Astrocytes exhibited more modest but still highly significant correlation ($\rho = 0.182$, $p \approx 10^{-22}$). Inhibitory neurons showed the weakest correlation ($\rho = 0.078$, $p \approx 10^{-9}$), consistent with greater transcriptional heterogeneity and lower signal-to-noise in this population. These results indicate that, even when upstream technical processing substantially alters the DEG landscape, the relative prioritization of predictive genes remains broadly preserved, particularly in glial cell types that are strongly implicated in AD pathology. To further assess robustness at the level of discrete gene sets, we defined predictive genes as those receiving nonzero feature importance in at least two of five cross-validation splits within each pipeline. We then quantified overlap between Mathys-derived and Murphy/Skene-derived predictor sets, reporting recall with respect to the smaller of the two sets to avoid bias from differences in predictor set size. Across cell types, we observed substantial overlap. Recall values ranged from 0.231 in Inhibitory Neurons to 0.660 in Oligodendrocytes, with intermediate values in Astrocytes (0.611), Microglia (0.637), OPCs (0.547), and Excitatory Neurons (0.442). Notably, glial populations again showed the highest concordance, with over half of the smaller predictor set recovered across pipelines in Astrocytes, Microglia, Oligodendrocytes, and OPCs. Importantly, this degree of overlap was observed despite the fact that the Murphy et al. reanalysis yields orders of magnitude fewer DEGs, underscoring that predictive modeling identifies a subset of genes that is far more stable to QC and statistical stringency choices than univariate differential expression alone. Together, these analyses demonstrate that TriSCOPE's predictive gene prioritization is robust to major revisions in snRNA-seq preprocessing and analytical methodology, directly addressing concerns regarding the sensitivity of DEG-based findings to upstream choices. While univariate differential expression results can vary substantially with processing and modeling decisions, the multivariate, donor-aware predictive framework used here consistently recovers overlapping gene importance patterns across independently processed versions of the same underlying data. These findings support the interpretation that predictive modeling captures coordinated transcriptional programs associated with disease state, rather than relying on marginal expression shifts that may be sensitive to specific DEG pipelines. As such, predictive feature prioritization provides a complementary and, in some respects, more stable approach to identifying disease-relevant transcriptional structure in single-cell data.

3) There are now much larger well characterised AD datasets available from ROSMAP (i.e. Mathys et al 2024) and Allen Brain Atlas. I encourage the authors to use those datasets for increased power.

Using the Mathys 2023 single-cell dataset, we performed a differential expression analysis of n=427 in order to contextualize our findings. We then performed a concordance analysis between this DEG analysis and our predictive model, the results of which we report. We further pulled from 4 large-scale previously published Alzheimer's single cell studies, and performed a concordance analysis between our model identified predictive genes and these previously identified DEGs. We detail the results of this, in our main results section titled "Predictive genes capture disease signal beyond differential expression". In that section we write:

We first performed expanded donor-aware differential expression analyses in a larger ROSMAP cohort comprising 427 individuals and evaluated concordance between differential expression and predictive feature importance. Across cell types, 2–8% of predictor genes were differentially expressed, indicating limited but non-random overlap between predictive features and donor-aware DEGs. Consistent with this, predictors showed significant enrichment among DEGs in multiple cell types (odds ratios ranging from ~2.8 to 6.9 in enriched cell types), while no enrichment was observed in inhibitory neurons and OPCs (Supplementary Table 18). In parallel, predictive importance exhibited a statistically significant but weak association with differential expression effect sizes across five of six cell types, with the strongest associations observed in Microglia and Oligodendrocytes. Spearman correlations between predictor rank and absolute log fold change were modest in magnitude ($\rho \approx 0.02$-0.21), indicating that although TriSCOPE predictors are enriched among DEGs in a subset of cell types, their predictive importance is not reducible to transcriptional effect size alone. We observed a similar qualitative pattern when comparing TriSCOPE predictors to differential expression rankings from four previously published single-cell Alzheimer's disease studies spanning multiple brain regions, with modest rank concordance in glial cells (mean Spearman $\rho \approx 0.26$ in microglia, 0.18 in oligodendrocytes, 0.30 in OPCs) and moderate concordance in excitatory neurons (mean Spearman $\rho = 0.235$)—and weaker correlations in inhibitory neurons and astrocytes (Supplementary Table 18).

Minor:

1. The manuscript file lacks page numbers and line numbers, so it's difficult to point out the concerns page by page.

We have updated the manuscript to include page numbers and line numbers.

2. In the TriScope method section, the authors mentioned "Genes with low mean expression or high sparsity were excluded prior to feature selection." Please report how many genes were left after this filtering.

The number of genes left after filtering varied on a per-cell type basis. We have updated our methods section to include the number of genes left after filtering for each cell type. Specifically we write in the methods:

After cell-type–specific expression and missingness filtering, the final gene sets used for modeling comprised 2,971 genes in astrocytes (Ast), 1,339 genes in microglia (Mic), 3,824 genes in

oligodendrocyte precursor cells (Opc), 1,751 genes in oligodendrocytes (Oli), 5,637 genes in inhibitory neurons (In), and 7,591 genes in excitatory neurons (Ex).

3. Had the authors identified any limitations, they should report that in the limitation section.

We have modified our discussion section to make the limitations of our study clear. We now have an explicit section that begins with "Our study has several limitations," which describes in detail the limitations of our work. We now write:

Our study has several limitations: firstly, our framework is predictive in design, but its primary aim is feature discovery rather than clinical prognostication. The AUCs reflect the use of post-mortem, cross-sectional data, where prediction cannot be the endpoint; instead, the value lies in identification of key genes and cell states that may reflect AD vulnerability independent of known risk factors, and thus warrant further investigation. Importantly, the cross-sectional nature of the data precludes inference about temporal ordering, such that we cannot determine whether the identified transcriptional signatures precede disease onset, track disease progression, or represent late-stage reactive responses to neuropathology. Longitudinal inference, approximated through integration with longitudinal clinical or imaging trajectories or via matched multi-region sampling within the same individuals across brain regions affected at different stages of Alzheimer's disease, would better align molecular variation with disease stage, potentially strengthening signal-to-noise and improving predictive discrimination. As such, we believe that perturbation experiments will be essential to disentangle whether these transcriptional signatures represent protective adaptations, pathogenic drivers, or reactive epiphenomena. These perturbation experiments, when coupled with our subcluster-level findings, open up avenues for more targeted therapeutic studies. Additionally, we chose to focus on the prefrontal cortex in AD, since it represents a brain region most directly responsible for executive function, where we expected see transcriptional changes associated both with Alzheimer's neuropathology and cognitive decline, however, this focus limits the generalizability of our findings to other brain regions that are affected earlier or differently over the course of disease progression, such as the hippocampus, entorhinal cortex, or subcortical nuclei. Lastly, at present there remains a paucity of high-powered cohort data to examine case-control single cell status of AD [56], meaning future directions include expanding the current resources in sample size to further explore and resolve heterogeneity.

Reviewer #1 (Remarks on code availability):

N/A

Reviewer #2 (Remarks to the Author):

The authors train a predictive model of AD case control status using a single cell data-set from the ROSMAP cohort (the Mathys et al. data) and identify cell type specific predictors of AD status which they validate in an independent data-set (from Lau et al). They then show that the most predictive features from the cell type expression predictor of AD are enriched in known AD genetic loci. They also apply pseudotime inference to the single cell RNA-seq data and show enrichment for relevant AD pathways (e.g. Amyloid processing).

The study is focused on machine learning approaches to explore the relationship between variation in single cell expression and disease diagnosis (particularly with the focus on disease state classification). I have some major concerns with the design and novelty of the study that I've outlined below along with some minor concerns. Overall the paper could use some aggressive editing and streamlining to focus on what is novel about their approach compared to what has already been published on single-cell RNAseq analyses in AD.

Major Concerns

1. Why use such a small training (n=48) and validation data-set (n=21)? And also why not use some more recently published data as well? Both studies they use are more than five years old. There are now publicly available single cell data-sets on hundreds if not thousands of unique participants from various sources derived including SEA-AD (https://doi.org/10.1038/s41593-024-01774-5), additional ROSMAP data (https://doi.org/10.1038/s41588-024-01685-y & https://doi.org/10.1016/j.cell.2023.08.039), MSBB (https://doi.org/10.1038/s41597-023-02507-2), AZSAND (https://doi.org/10.1038/s41467-024-49790-0), and others.

We thank the reviewer for their feedback.

We agree that recent large-scale datasets substantially strengthen the evaluation of our approach. In response to this comment, we have extended our analyses to incorporate more recent and substantially larger cohorts. Specifically, we performed differential expression analyses using the two expanded ROSMAP single-cell datasets (Mathys et al., 2023 which has n = 427 donors and 2.3 million cells, and Mathys et al., 2024, which has n = 48 donors and 254,721 cells) and assessed concordance between genes identified by our predictive model and DEGs derived from this larger cohort. We further evaluated overlap with genes reported across multiple large-scale Alzheimer's disease single-cell studies, and now report both concordance and discordance in the main text and Supplementary Information. We would like to clarify that the primary goal of this study is not to optimize predictive performance on any single dataset, but rather to identify robust, disease-relevant gene predictors that persist across cohorts, preprocessing choices, and analytical assumptions. We note, that retraining full machine-learning models independently on each external dataset may also conflate biological signal with cohort-specific technical and experimental variation (e.g., sequencing chemistry, cell annotation strategies, and QC thresholds), making direct comparison of learned predictors difficult to interpret. Instead, our evaluation strategy focuses on cross-cohort robustness and generalization of predictive features, assessed through (i) concordance with donor-aware differential expression in substantially larger ROSMAP cohorts, (ii) overlap with genes reported across multiple independent single-cell Alzheimer's disease studies, (iii) external validation of classifier performance and feature overlap in an independent held-out dataset, and (iv) orthogonal biological validation via enrichment for AD GWAS–linked genes using conditional colocalization analyses and consistency with disease-associated transcriptional trajectories inferred from pseudotime analyses. This design allows us to test whether genes prioritized by TriSCOPE capture disease-relevant signals that are stable across datasets, rather than whether a model can be re-fit to maximize performance within a given cohort. We note that retraining models on each dataset is an important but distinct objective, and we view large-scale cross-study model training as a valuable direction for future work as harmonized, multi-cohort single-cell resources continue to mature. *Still, to address concerns related to small n, we incorporate expanded cohorts into our analysis, designed to test biological robustness and generalizability:*

In the results section titled "**Predictive genes capture disease signal beyond differential expression**" we write:

To assess whether machine learning–derived predictors capture disease-relevant signal beyond that identified by differential expression, we measured their concordance with donor-aware DE analyses in an expanded ROSMAP cohorts and across multiple independent Alzheimer's disease datasets. Across these, our analysis included over 540 unique individuals, with additional external validation across results from four independent studies (n=94). We first performed expanded donor-aware differential expression analyses in a larger ROSMAP cohort comprising 427 individuals and evaluated concordance between differential expression and predictive feature importance. Across cell types, 2–8% of predictor genes were differentially expressed, indicating limited but non-random overlap between predictive features and donor-aware DEGs. Consistent with this, predictors showed significant enrichment among DEGs in multiple cell types (odds ratios ranging from ~2.8 to 6.9 in enriched cell types), while no enrichment was observed in inhibitory neurons and OPCs (Supplementary Table 18). In parallel, predictive importance exhibited a statistically significant but weak association with differential expression effect sizes across five of six cell types, with the strongest associations observed in Microglia and Oligodendrocytes. Spearman correlations between predictor rank and absolute log fold change were modest in magnitude ($\rho \approx$ 0.02-0.21), indicating that although TriSCOPE predictors are enriched among DEGs in a subset of cell types, their predictive importance is not reducible to transcriptional effect size alone. We observed a similar qualitative pattern when comparing TriSCOPE predictors to differential expression rankings from four previously published single-cell Alzheimer's disease studies spanning multiple brain regions, with modest rank concordance in glial cells (mean Spearman $\rho \approx$ 0.26 in microglia, 0.18 in oligodendrocytes, 0.30 in OPCs) and moderate concordance in excitatory neurons (mean Spearman $\rho = 0.235$)—and weaker correlations in inhibitory neurons and astrocytes (Supplementary Table 18).

In the results section titled "**<u>Generalization of Predictive Genes</u>**" we write: We next evaluated cross-cohort generalization of cell-type-specific classifiers using an independent validation dataset comprising 12 clinical AD and 9 clinical control individuals, totaling 169,496 nuclei across Microglia, Oligodendrocytes, Excitatory Neurons, Inhibitory Neurons, and Astrocytes (Supplementary Table 9). Using one-shot classifiers from our original dataset, we showed consistent predictive performance across cell types suggesting the generalizability of our models (Supplementary Fig. 1a). Gene-only one shot classifiers showed mean AUCs ranging from 0.56 (Microglia) - 0.67 (Astrocytes) (Supplementary Fig. 1b). To assess the concordance of feature importance in both datasets, we fit autoML models from scratch on our validation dataset. We found strong overlap in predictive genes (FDR adjusted Fisher's $p < 0.05$ in all cell types), including 40 overlapping predictors in Astrocytes (OR = 30.28) and 106 overlapping in Oligodendrocytes (OR = 21.78) (Supplemental Fig. 1a, Supplemental Table 11).

2. They focused primarily on a machine learning approach to identify features of interest. I suspect that the most correlated features with disease state within each cell population will be very similar to the most predictive features in the GBM approach - why isn't there a direct comparison of the ordering of predictive features and the ordering of differentially expressed genes? Apologies if it is there, I looked for it but could not find it.

We performed a Spearman's rank correlation between our predictors and differentially expressed genes (as identified using the Random-effects model) and have included the results in the Supplemental Results under a section titled "DEG Correlation Analysis". This section further includes results from correlation analysis of our DEGs with validation DEGs. Following our n=427 validation analysis, we performed the same Spearman rank correlation, this time between predictors identified in our initial dataset, and DEGs identified in our n=427 validation dataset. These results are reported under the section "Predictive genes capture disease signal beyond differential expression" in the main results.

Below is the included result from our Supplemental Results: We first assessed concordance between genes identified as predictive by TriSCOPE and genes identified as differentially expressed via a random effects model in our n = 48 cohort. Across all major cell types, predictive gene rankings were positively and significantly correlated with DEG rankings defined using FDR-adjusted p-values (Spearman \rho = 0.04-0.48; all FDR < 0.005), with the strongest agreement observed in microglia. Notably, the magnitude of correlation varied substantially by cell type, ranging from weak but significant concordance in inhibitory neurons to moderate–strong concordance in glial populations. This partial alignment indicates that while TriSCOPE-derived predictors are biologically anchored in differential expression, predictive modeling captures multivariate disease-associated structure that is not explained by univariate differential expression alone.

Below is the included result from our Main Results:
To assess whether machine learning–derived predictors capture disease-relevant signal beyond that identified by differential expression, we measured their concordance with donor-aware DE analyses in an expanded ROSMAP cohorts and across multiple independent Alzheimer's disease datasets. Across these, our analysis included over 540 unique individuals, with additional external validation using results from four independent studies (n=94). We first performed expanded donor-aware differential expression analyses in a larger ROSMAP cohort comprising 427 individuals and evaluated concordance between differential expression and predictive feature importance. Across cell types, 2–8% of predictor genes were differentially expressed, indicating limited but non-random overlap between predictive features and donor-aware DEGs. Consistent with this, predictors showed significant enrichment among DEGs in multiple cell types (odds ratios ranging from ~2.8 to 6.9 in enriched cell types), while no enrichment was observed in inhibitory neurons and OPCs (Supplementary Table 18). In parallel, predictive importance exhibited a statistically significant but weak association with differential expression effect sizes across five of six cell types, with the strongest associations observed in Microglia and Oligodendrocytes. Spearman correlations between predictor rank and absolute log fold change were modest in magnitude ($\rho \approx$ 0.02-0.21), indicating that although TriSCOPE predictors are enriched among DEGs in a subset of cell types, their predictive importance is not reducible to transcriptional effect size alone. We observed a similar qualitative pattern when comparing TriSCOPE predictors to differential expression rankings from four previously published single-cell Alzheimer's disease studies spanning multiple brain regions, with modest rank concordance in glial cells (mean Spearman $\rho \approx$ 0.26 in microglia, 0.18 in oligodendrocytes, 0.30 in OPCs) and moderate concordance in excitatory neurons (mean Spearman $\rho = 0.235$)—and weaker correlations in inhibitory neurons and astrocytes (Supplementary Table 18).

3. There are snRNA-seq papers showing enrichment for genetic loci in genes that are correlated with disease status from single cell data. Specific examples include APOE (https://doi.org/10.1038/s41593-024-01774-5, https://doi.org/10.1038/s41588-024-01685-y), CLU (https://doi.org/10.1186/s13073-022-01136-5), BIN1 (https://doi.org/10.1038/s41593-019-0539-4) and particularly Mathys et al. 2024 (https://doi.org/10.1038/s41586-024-07606-7) overall. How are their results materially different from these previous observations?

Our analysis differs in a few key ways from these previous works. Firstly, TriSCOPE is a multivariate, phenotype-predictive framework, which is distinct from the univariate disease-associated gene analyses that have previously been leveraged. We show that our predictors, though they do have overlap with DEGs, are in many cases unique from DEGs, and furthermore that predictors often represent a much smaller subset of genes than DEGs. Furthermore, our study focuses on these genes both from a clinical AD standpoint and a neuropathological AD standpoint, which is distinct from many previous AD atlas works, which blend these phenotypes. We include two sections that discuss this distinction, titled "**Clinical and Neuropathological Predictors Diverge Across Cell Types**" and "**Transcriptional Analysis of Divergent clinical outcomes under matched neuropathology**". These sections use both our predictive modeling approach and a more traditional differential expression analysis approach.

Our paper also performs a conditional colocalization, where we perform colocalization restricted to TriSCOPE-prioritized predictors. This is not "GWAS enrichment among DEGs/correlated genes", but rather a more stringent post-prioritization test asking: within an ML-derived predictive set, which genes show shared causal architecture with AD GWAS via brain eQTLs. We make note of genes that are not differentially expressed but become stronger candidates after conditional colocalization. For example, we write in our Discussion section: For example, *USP6NL* and *EGFR* were not significantly differentially expressed but were identified as modest predictors in expression-based models; our conditional GWAS–eQTL colocalization then further strengthened their genetic anchoring, elevating them as genetically supported, cell-type–specific candidates whose regulatory variation likely contributes to AD risk but would be missed by differential expression–based prioritization alone.

Lastly, the use of an integrated three-fold approach for gene prioritization is distinct from previous papers that have focused solely on Differential expression analysis or solely on pseudotime analysis. By performing three independent analyses across multiple cohorts and studies, we show that genes prioritized by TriSCOPE are reproducible across analytical paradigms and datasets, whereas many previous studies have relied on just one of these analytical approaches to establish biological plausibility.

We appreciate the reviewer's question regarding novelty and agree that clarifying the distinction between bulk and single-cell scale is essential. While several prior Alzheimer's disease studies have analyzed hundreds of donors using bulk RNA-seq, single-cell and single-nucleus studies to date have been substantially smaller in both cellular and donor scale. To our knowledge, no prior study of Alzheimer's disease has performed cell-level, donor-held-out predictive modeling at the scale examined here. In aggregate, our study analyzes 2,797,869 single nuclei across 544 unique

donors, all at single-cell resolution. By contrast, many of the large-cohort studies of AD (e.g., Mathys et al. 2023, n ≈ 427 donors) are single-nucleus RNA-seq analyzed primarily at the pseudobulk level and therefore do not resolve cell-type–specific transcriptional programs at the cell level or permit cell-level predictive modeling. Existing single-cell Alzheimer's studies typically profile fewer than 200,000 cells and fewer than 100 donors (e.g., Mathys et al. 2019, n = 48 donors, ~80,000 nuclei; Morabito et al., ~70,000 nuclei; Lau et al., ~120,000 nuclei), and most do not implement donor-aware random-effects differential expression models. The primary novelty of our work lies in bridging donor-scale inference and single-cell resolution. We perform multivariate, donor-held-out predictive modeling and donor-aware differential expression across millions of nuclei, enabling robust identification of cell-type–specific transcriptional programs associated with clinical and neuropathological Alzheimer's disease. We further integrate these results with pseudotime analysis and conditional GWAS–eQTL colocalization to prioritize reproducible, genetically supported candidates. Thus, rather than replicating prior bulk or small to medium-scale single-cell analyses, our study establishes a framework for donor-aware, cell-type–resolved disease modeling at unprecedented single-cell scale, which we believe represents a substantive advance over existing approaches.

In brief, standard DE answers which genes differ marginally; TriSCOPE aims to answer which genes still matter when everything else is known. To make this distinction clear we now include an explicit "Why Machine Learning?" section in our supplemental discussion section. Furthermore in our main discussion section we write: A central strength of TriSCOPE lies in its use of supervised learning to prioritize a concise, stable set of disease-relevant genes that contribute jointly to disease discrimination, including genes that do not meet conventional univariate significance thresholds but are consistently informative in multivariate models. This capability is essential in high-dimensional single-cell data, where marginal testing alone cannot resolve redundancy among correlated transcripts or identify features that are conditionally informative given the broader transcriptional context. Differential expression alone often yields thousands of marginally associated genes per cell type, many of which reflect correlated or redundant signals. By contrast, TriSCOPE's supervised framework introduces competition among correlated features, prioritizing genes that remain informative when modeled jointly across donors and covariates. Empirically, this compresses candidate lists by ~5–10× relative to donor-aware DE while retaining reproducible disease signal, enabling downstream genetic anchoring and experimental prioritization.

4. The disease pseudotime aspect of the paper is confusing to me - what exactly is it adding to the story that is not already captured in the predictive disease features and differential expressed genes? Why not have a direct comparison between all of their analyses somewhere for the reader to understand what is similar and what is different? Also how is it materially different than previous pseudotime (or pseudotrajectory) observations made in other snRNA-seq AD papers (e.g. from SEA-AD)?

We agree that disease pseudotime can be confusing without a clear articulation of what it adds beyond predictive modeling and differential expression, and we appreciate the opportunity to clarify its role. Firstly, to make the distinct roles of predictive modeling, differential expression, and pseudotime explicit, we now include an overview schematic (Supplementary Fig. 1f) that contrasts the inputs, outputs, primary questions, and typical use cases of each analytical axis. Additionally, in the final section of our results, titled: "**Integrated TriSCOPE identifies convergent, cell-type–specific Alzheimer's disease programs**" in which we justify why our analysis integrates these three approaches, and what each arm of our approach contributes.

Diffusion pseudotime provides an ordering of cells along a continuous disease-associated trajectory, allowing us to ask which genes exhibit monotonic, progressive behavior aligned with disease advancement, rather than only static case–control differences. In this sense, pseudotime addresses a distinct biological question: which genes explain gradual, trajectory-aligned cellular transitions along a continuous disease-associated axis. This allows us to treat pseudotime as a third, orthogonal axis for gene nomination. We can then ask whether genes that explain these continuous transitions overlap with genes independently identified by our two static case–control approaches—predictive modeling and differential expression.

With regard to how pseudotime provides an independent analysis of disease, we use pseudotime as an unsupervised gene-prioritization tool by extracting genes with the largest loadings on the principal component most correlated with pseudotime; importantly, these gene rankings are computed after trajectory inference and are not used to construct or orient the trajectories. Furthermore, in our updated HVG based pipeline, neither predictive modeling nor differential expression influences the genes used in pseudotime analysis, meaning that identified genes are nominated via an orthogonal axis. We have revised our methods section, under "Pseudotime inference and trajectory analysis," to make this distinction and approach clear.

Pseudotime inference and trajectory analysis To characterize continuous disease-associated transcriptional trajectories within defined cell subclusters, we performed pseudotime analysis using a non-circular, unsupervised feature selection strategy anchored to control-like cellular states. All analyses were conducted independently within each annotated subcluster to avoid confounding by broad cell-type differences. Among the 41 subclusters identified by Mathys et al. (2019), we analyzed those containing at least 50 cells and representation from both Alzheimer's disease (AD) and control individuals. For each eligible subcluster, we constructed a subcluster-specific expression matrix from the normalized single-nucleus RNA-seq count matrix. Expression values were library-size normalized to a target sum of 10,000 counts per cell and log-transformed prior to downstream analysis. To avoid circularity between trajectory inference and disease association, we selected features for pseudotime construction using highly variable genes (HVGs) within each subcluster, rather than differentially expressed genes or model-derived predictors. Specifically, within each subcluster, we identified the top 2,000 HVGs using the Seurat variance-stabilization method as implemented in Scanpy, or all genes if fewer than 2,000 were available. Only HVGs were used for dimensionality reduction and trajectory inference. Principal component analysis (PCA) was performed on the HVG-restricted expression matrix, retaining up to 30 principal components (or fewer if limited by gene number). A k-nearest neighbor graph was constructed using these components ($k = 10$), followed by diffusion map embedding to capture continuous cellular transitions. Diffusion pseudotime (DPT) was used to infer continuous trajectories within each subcluster. To orient trajectories in a biologically interpretable manner while avoiding disease-label leakage, root cells were selected based on neuropathological and cognitive severity measures rather than transcriptomic similarity or classifier outputs. For each subcluster and each random seed, candidate root cells were restricted to those with available CERAD neuritic plaque scores and cognitive diagnosis values. Among these, the root was chosen as a cell with the maximal CERAD score (least pathological burden) and minimal cognitive impairment score, corresponding to the most control-like pathological state. When multiple cells satisfied these criteria, one was selected at random using a fixed seed. This procedure ensured consistent anchoring of pseudotime to a putative healthy baseline while allowing robustness assessment. Diffusion pseudotime was computed separately for each seed using the selected root cell. To assess the stability of inferred

trajectories, pseudotime inference was repeated across multiple random seeds per subcluster. For each seed, diffusion pseudotime values were computed for all cells, and the association between pseudotime and disease status was evaluated using receiver operating characteristic (ROC) analysis and logistic regression. To aggregate pseudotime estimates across seeds while preserving relative ordering, pseudotime values were rank-normalized within each seed and averaged across seeds to obtain a mean pseudotime score per cell. This aggregated pseudotime was used for all downstream analyses, including visualization and gene association testing. To identify genes most strongly associated with progression along the inferred trajectory, we examined the relationship between aggregated pseudotime and principal component structure. For each subcluster, we identified the principal component whose scores exhibited the strongest Spearman correlation (by absolute value) with mean pseudotime. Gene loadings from this component were interpreted as trajectory-influencing weights. Genes were ranked by the absolute value of their loadings on the selected component. For each subcluster, we defined the top 20 genes by absolute loading as trajectory-influencing genes, corresponding approximately to the top 1% of genes contributing to the pseudotime-aligned transcriptional axis. These genes were recorded for downstream aggregation and validation analyses. To identify target genes repeatedly implicated by trajectory analysis, we quantified the frequency with which each gene appeared among the top trajectory-influencing genes across all subclusters. These analyses were performed strictly as post hoc summaries of trajectory structure and did not influence pseudotime construction, feature selection, or thresholding. The relationship between pseudotime and disease status was quantified using logistic regression models and ROC analysis. For clinical diagnosis, AD status was defined based on dfcdx_lv, while a separate analysis used CERAD plaque burden to define neuropathology-based disease status. Models were fit independently for each seed and aggregated across seeds using Fisher's method for p-value combination and inverse-variance aggregation for effect sizes. Trajectory inference and gene ranking were performed without reference to disease labels or model-derived predictors.

5. The authors use very strong language to describe the genes that they prioritize with their analysis e.g. from the abstract - "our study nominates high-confidence, genetically anchored molecular drivers of AD and prioritizes them for mechanistic investigation and therapeutic targeting in age-related neurodegenerative disease". I am not convinced that the authors have shown anything but some evidence that transcriptional regulation of a given locus is associated with AD status and is dysregulated in AD brains within a certain cell popluation. A great example of this is ARL17B - a more parsimonious possible explanation given what we know about the role of tau pathology in AD may be that MAPT is the causal gene within the locus but MAPT protein expression (or perhaps phopho-tau expression) is co-regulated with ARL17B mRNA expression and so is invisible in the expression data view. The authors should tone down the language with this sort of caveat.

We have toned down the strong language that we previously used to describe the genes prioritized by our integrative framework, and now emphasize that these genes should be interpreted as high-confidence candidates supported by convergent evidence, rather than definitive causal drivers. Importantly, our claims are grounded not in transcriptional association alone, but in the convergence of four orthogonal signals—multivariate predictive relevance, donor-aware differential expression, pseudotime-based trajectory inference, and conditional GWAS–eQTL colocalization. In this context, loci

such as ARL17B are not asserted to be the sole causal effectors; rather, our analyses provide strong evidence that genetic risk at the locus is mediated through transcriptional regulation captured at the single-cell level, even in the presence of alternative mechanistic explanations (e.g., co-regulation with MAPT protein or tau pathology). We therefore frame these genes as genetically anchored molecular candidates that warrant mechanistic follow-up, while explicitly acknowledging that causal resolution will require perturbational and proteomic validation.

Minor Concerns

1. Did they try other approaches for prediction of disease state than a GBM? Does model selection matter?

Yes. To assess whether our findings depend on a particular modeling choice, we performed a systematic benchmarking of multiple commonly used classifiers for disease-state prediction. As described in the new Supplementary Results section *"Benchmarking of Model Architectures"*, we evaluated four architectures—gradient boosted decision trees (GBM), LASSO-regularized logistic regression, elastic net regression, and random forest—using identical preprocessing, donor-aware train/test splits, feature filtering, and evaluation protocols across all six major cell types.
Across cell types, the optimal architecture varied, with GBM achieving the highest mean AUC in oligodendrocytes and microglia, LASSO performing best in astrocytes and excitatory neurons, and random forest performing best in inhibitory neurons and OPCs. Importantly, however, no single model architecture consistently outperformed the others, and when GBM was not the top-performing model, the absolute performance differences were modest (ΔAUC = 0.021–0.077) (Supplemental Table 23). This indicates that the predictive signal captured by TriSCOPE is robust across modeling paradigms and not driven by a specific classifier.

We therefore focus on GBM models in the main text not because they uniquely maximize performance, but because they offer a favorable balance of competitive accuracy, the ability to model nonlinear feature interactions, and stable, interpretable feature-importance estimates that integrate naturally with downstream differential expression, genetic anchoring, and pseudotime analyses. Together, these benchmarking results demonstrate that model selection does not materially affect the biological conclusions of our study.

2. Did they try to fit a model with multiple cell types to predict disease state? Is a multi cell type model more predictive of disease state?

In the Results subsection titled, "Predicting AD using Demographics, APOE genotype, and gene expression", we train a multi–cell type ("all cells") baseline model over >230,000 nuclei by pooling all cell types together, integrating demographics + APOE + gene expression. That pooled model achieves mean AUC = 0.545 ± 0.118, compared to 0.528 ± 0.138 for demographics+APOE alone. We then show that cell type–specific models are substantially more predictive: after stratifying into the six major populations and training within each, performance increases to mean AUC 0.620 ± 0.072 to 0.695 ± 0.052 (gene expression + APOE), which is materially higher than the pooled baseline. Furthermore, we show that predictive AUCs further increase at the subcluster resolution we detail in the results section titled: "**Subcluster Level Expression Improves AD Prediction**". In our discussion section we write: Applying TriSCOPE across increasing levels of cellular resolution revealed a structured increase in disease-discriminative signal, with modest performance at global levels and the strongest signal emerging within biologically coherent subclusters.

3. I wasn't able to open the supplementary tables.

We apologize for the issue in opening the supplementary tables. We have reattached all tables with our revised submission.

Reviewer #2 (Remarks on code availability):

I did not try to run the code, but I did look through all of the attached jupyter notebooks and the approach looks very reasonable and should work.

Reviewer #3 (Remarks to the Author):

This manuscript introduces TriSCOPE as an integrative framework that combines multivariate machine learning, differential expression, and pseudotime analysis to identify cell-type and subcluster-specific programs associated with clinical and neuropathological Alzheimer's disease, with cross-cohort validation and genetic anchoring to GWAS/eQTL signals. The study is thoughtfully motivated and biologically plausible, recovering known AD features while nominating underexplored candidates. It usefully contrasts clinical vs pathology readouts across cell types. At the same time, important clarifications and additions would strengthen impact and adoptions; specifically clearer articulation and empirical benchmarking of TriSCOPE;s added value over standard pipelines (univariate DE, pseudotime/GLMs or widely used integrative models), broader validation and sensitivity analyses to solidify robustness and reproducibility/usability improvements (open code, tutorials and compute scaling). In short, the work while promising and lose to broadly useful, still requires additional evidence and clearer communication of when and why TriSCOPE should be preferred over "normal analysis". As a prospective used, I still find it not fully clear what unique decision(s) TriSCOPE enables beyond standard DE + pathway + pseudotime and the manuscript should explicitly define the core use-case and demonstrate a measurable gain over baselines on both cohorts.

We thank the reviewer for their critique and feedback.

Major Comments
1. It is strongly advised to the authors to benchmark TriSCOPE against alternative integrative methods (e.g., LASSO/logistic, MOFA+, scVI/scANVI, deep embeddings) and report comparative performance with confidence intervals.

We have now systematically benchmarked TriSCOPE's predictive modeling component against three additional, widely used supervised learning architectures: LASSO-regularized logistic regression, elastic net regression, and random forest, using identical preprocessing, donor-aware splits, feature filtering, and evaluation protocols. Across six major brain cell types, we found that model architecture did not have a material effect on predictive performance or downstream biological conclusions, with different architectures performing marginally better in different cell types but no single model consistently outperforming the others. When GBM was not the

top-performing model, the absolute performance differences were small (ΔAUC = 0.021–0.077), indicating that TriSCOPE's signal is robust across modeling paradigms rather than driven by a specific classifier. Full benchmarking results are provided in the new Supplementary Results section, *"Benchmarking of Model Architectures."*

We respectfully note that methods such as MOFA+, scVI/scANVI, and deep embedding approaches address fundamentally different objectives from those of TriSCOPE. These methods are primarily designed for unsupervised or semi-supervised latent factor discovery, batch correction, or representation learning, and do not natively optimize disease-state prediction, yield directly interpretable gene-level importance scores, or integrate naturally with donor-aware supervised evaluation. In contrast, TriSCOPE is explicitly designed as a supervised, phenotype-predictive framework whose central goal is interpretable gene prioritization conditioned on disease status, followed by integration with differential expression, pseudotime, and genetic anchoring. As such, direct performance comparisons to unsupervised latent embedding models would not be methodologically appropriate or informative for the questions addressed in this study.

Taken together, our expanded benchmarking demonstrates that TriSCOPE's conclusions are not dependent on a particular classifier choice, and that the framework's biological insights arise from the integration of complementary analytical axes rather than from any single modeling architecture. The fully rewritten supplemental results section, "Benchmarking of Model Architectures" is presented below:

Benchmarking of Model Architectures

At a cell-type level, the best-performing architecture varied. In Astrocytes, LASSO achieved the highest mean AUC (0.589 ± 0.055), followed closely by elastic net (0.575 ± 0.050) and GBM (0.568 ± 0.064). In Excitatory neurons: LASSO again performed best (0.607 ± 0.054), with elastic net yielding comparable performance (0.603 ± 0.054), while GBM achieved a lower mean AUC (0.530 ± 0.042). In Inhibitory neurons Random forest performed best (0.656 ± 0.043), followed by GBM (0.628 ± 0.074). In Oligodendrocytes: GBM achieved the highest performance (0.652 ± 0.096), outperforming random forest (0.619 ± 0.077) and linear models. Oligodendrocyte progenitor cells (OPCs): Random forest achieved the highest mean AUC (0.569 ± 0.065), with elastic net and LASSO performing similarly. Microglia: GBM yielded the strongest performance (0.653 ± 0.085), marginally exceeding LASSO (0.651 ± 0.066).

Overall, GBM achieved the highest mean AUC in two of six cell types (Oligodendrocytes and Microglia), while LASSO and random forest were optimal in other cell populations. Importantly, when GBM was not the top-performing model, the absolute performance gap between GBM and the best model was small (ΔAUC ranging from 0.021 to 0.077), indicating that no single architecture dramatically outperformed others. These results demonstrate that TriSCOPE's predictive signal is robust across multiple modeling paradigms and that downstream biological conclusions do not depend on a specific classifier. We therefore focus on GBM models in the main text, as they balance predictive performance, nonlinear modeling capacity, and stable feature-importance estimates suitable for interpretation and integration with differential expression and pseudotime analyses.

2. It would be also important to expand the validation beyond the PFC by testing cross-region and cross-cohort generalizability (e.g., hippocampus, other public datasets).

We appreciate the reviewer's suggestion to expand validation across brain regions and cohorts. We now clarify in the Introduction and Discussion why we focused on the dorsolateral prefrontal cortex (PFC) and have added additional cross-study validation analyses to address generalizability.

The PFC was selected *a priori* because it is a brain region centrally involved in executive function, one of the cognitive domains most strongly affected in clinically manifest Alzheimer's disease. Importantly, relative to regions such as the hippocampus or entorhinal cortex—which are affected earlier in the disease course—the PFC is typically involved at later stages of AD progression. We therefore hypothesized that transcriptional changes in the PFC would be particularly informative for studying the divergence between neuropathological burden and clinical symptom manifestation, as damage to the PFC is more likely to coincide with overt clinical diagnosis. This makes the PFC a biologically appropriate region for interrogating molecular programs associated with clinical AD status, rather than early pathological changes alone.

To address concerns about generalizability beyond the PFC, we have now expanded our validation analyses by comparing TriSCOPE-prioritized genes to results from independent single-nucleus RNA-seq studies profiling other brain regions, including datasets focusing on the hippocampus and entorhinal cortex. Specifically, we performed concordance analyses with published single-cell studies from Morabito et al. and Grubman et al., which profile medial temporal lobe regions affected earlier in AD. Across these comparisons, we observed significant but partial overlap, particularly in glial populations, supporting the robustness of a subset of prioritized genes across regions while also highlighting region-specific transcriptional programs. Specifically in our results we write:

We observed a similar qualitative pattern when comparing TriSCOPE predictors to differential expression rankings from four previously published single-cell Alzheimer's disease studies spanning multiple brain regions, with modest rank concordance in glial cells (mean Spearman $\rho \approx 0.26$ in microglia, 0.18 in oligodendrocytes, 0.30 in OPCs) and moderate concordance in excitatory neurons (mean Spearman $\rho = 0.235$)—and weaker correlations in inhibitory neurons and astrocytes (Supplementary Table 18).

We interpret the divergence in correlation between glial and neuronal results as reflecting region- and stage-specific neuronal transcriptional programs, whereby neuronal DE in hippocampal and entorhinal cortex datasets likely captures earlier, region-localized pathological processes that may not strongly overlap with PFC-derived predictors optimized for clinically manifest disease.

Together, these analyses support a model in which TriSCOPE identifies both shared, cross-region molecular signals and region-specific programs linked to disease stage and clinical manifestation. While our primary analyses focus on the PFC to address questions of clinical–pathological dissociation, we acknowledge that extending this framework to additional brain regions is an important direction for future work as larger, well-powered single-cell datasets become available for those regions.

3. I suggest the authors should perform sensitivity analyses that explicitly control for donor and technical confounders (PMI/RIN/batch/co-pathologies) and show robustness of features and AUCs under these controls.

We agree that sensitivity analyses controlling for donor and technical confounders are critical for establishing robustness. Accordingly, we performed multiple complementary analyses to assess the impact of donor structure and technical covariates on both feature identification and effect-size estimation.

First, all primary differential expression (DE) analyses were conducted using donor-aware mixed-effects models that include a donor-specific random intercept. This random effect is intended to capture donor-linked sources of variation, including post-mortem interval (PMI), RNA integrity (RIN), batch effects, and other unmeasured technical or biological factors (such as co-pathologies) that are shared across all cells from a given individual. Because PMI and RIN are constant within donor, they are perfectly collinear with donor identity. In the mixed-effects models with a donor-specific random intercept that we now implement, donor-level covariates—including PMI, RIN, batch, and other shared technical factors—are accounted by the random effect. By modeling donor as a random effect, these sources of variation are absorbed at the donor level rather than attributed to disease status, thereby reducing confounding arising from the nested structure of single-nucleus RNA-seq data.

Second, as an explicit sensitivity analysis, we independently fit two fixed-effects DE models controlling for covariates. The first controlled for three donor-level covariates: age at death, sex, and post-mortem interval (PMI), without donor random effects. The second controlled for five donor-level covariates: age at death, sex, APOE genotype, sequencing batch, and post-mortem interval (PMI), without donor random effects. We then compared effect-size estimates between the fixed-effects and random-effects specifications. Across all major cell types, $\log_2$ fold-change estimates were highly concordant between the approaches (as we now report in the supplemental results section titled “Fixed-Effect DEG Analysis”), indicating that inferred transcriptional effect sizes are robust to model specification, while the inclusion of donor random effects primarily regulates statistical significance rather than the direction or magnitude of effects. RNA integrity number (RIN) values were not available in the publicly released ROSMAP single-nucleus RNA-seq metadata (Synapse ID: syn18485175) and are therefore listed as NA. Because RNA integrity is a donor-level property and cannot be independently estimated in the absence of donor-specific measurements, we addressed potential RNA-quality–related confounding using donor-aware mixed-effects models, in which donor-level technical variation is absorbed by the random intercept

Third, we evaluated robustness of predictive performance (AUC) to donor and technical confounding using a strictly donor-aware evaluation design. All predictive models were trained and evaluated using donor-held-out splits, such that no cells from the same individual were ever present in both the training and test sets. This prevents leakage of donor-specific technical effects—including batch, PMI, RIN, or other donor-linked artifacts—into performance estimates and ensures that reported AUCs reflect generalization to *previously unseen donors* rather than memorization of donor identity. To further assess robustness, this donor-held-out evaluation was repeated across five independent randomized train–test splits, each with a distinct random seed and stratification by disease status and sex at the donor level. In addition, within each training split, model selection and hyperparameter optimization were performed using group-aware cross-validation (StratifiedGroupKFold), where folds were defined by donor identity. This ensures that donor structure is respected both during model fitting and during internal validation, and that AUC estimates are not inflated by donor- or batch-driven correlations. Together, this combination of donor-level splitting, repeated resampling, and group-aware cross-validation provides a conservative assessment of predictive performance and demonstrates that reported AUCs are robust to donor- and cohort-specific technical confounders.

Fourthly, we evaluated potential technical confounding in the predictive modeling framework. PMI did not differ significantly between AD and control donors in either the discovery or validation cohorts and was therefore not included as a predictive covariate. Importantly, unlike variables such as sex or APOE genotype—which take on a small number of shared values across many donors—PMI typically assumes a near-continuous, donor-specific value. As a result, including PMI in a cell-level predictive model can inadvertently encode donor identity, since all cells from a given donor share the same PMI value. In this setting, a classifier could spuriously associate PMI with disease status even in the absence of a true biological effect. To further guard against donor- or batch-driven artifacts, all predictive models employed strict donor-level train/test splits, ensuring that no cells from the same individual appeared in both training and testing sets and thereby preventing leakage of donor-specific signal into performance estimates.

Finally, robustness to cohort- and processing-specific effects is further supported by Spearman correlation analyses between our predictive modeling results and multiple independent cohorts and previously published single-cell AD studies generated using distinct experimental and analytical pipelines. The observation that partially overlapping disease-associated signals emerge despite differences in brain region, cohort composition, sequencing technology, and modeling framework provides additional evidence that the prioritized features reflect reproducible disease biology rather than cohort- or batch-specific artifacts.

4. It would be interesting to see if the pseudotime analysis is affected by circularity due to DE gene anchors. The authors should address circularity in pseudotime by constructing trajectories independent of DE gene selection and validating trajectory direction with orthogonal signals (e.g., amyloid burden per donor, pathology staging).

We thank the reviewer for highlighting the important issue of potential circularity in pseudotime analyses. We agree that our prior analysis—which constructed pseudotime trajectories using genes identified as differentially expressed with respect to disease—could, in principle, introduce bias and confound downstream interpretations.

In response, we have revised our pseudotime framework to explicitly eliminate this source of circularity. In the updated analysis, pseudotime trajectories are constructed *independently of differential expression testing and predictive feature selection*. Specifically, trajectories are inferred within each cell subcluster using highly variable genes (HVGs) selected solely based on expression variability using a rank-based procedure (top 2,000 genes by variance-normalized dispersion), without reference to disease status, DEG results, or TriSCOPE-prioritized predictors. No explicit variance threshold or disease-informed filtering is applied. This ensures that trajectory inference is driven by intrinsic transcriptional heterogeneity rather than disease-associated signals introduced a priori. Importantly, differential expression, predictive modeling, and genetic anchoring are now conducted independently of pseudotime construction, allowing us to test whether disease-associated genes—identified without reference to pseudotime—align with progression along an unsupervised trajectory, rather than enforcing such alignment by construction. This modification directly addresses concerns about circularity and allows pseudotime to serve as an orthogonal axis of evidence.

Finally, we emphasize that pseudotime in our revised framework is not used to *define* disease-relevant genes, but rather to contextualize when and how independently identified molecular programs vary along a continuous cellular progression. We have updated the Methods and Results to clarify this distinction and to explicitly acknowledge the limitations of our earlier DEG-anchored approach.

5. It would be suitable to provide APOE- and sex-stratified models and SHAP summaries to demonstrate genotype- and sex-specific predictor stability and divergence.

Supplementary Figure 4 provides APOE-stratified feature importance plots, and shows the stability/importance of various predictors across APOE genotypes. We have now included a figure showing sex-stratified feature importances as Supplementary Figure 4c (we have replaced the previous Supplementary Figure 4c, which featured Radar plots summarizing APOE-stratified SHAP values for selected top genes within each major cell type). Supplementary Figure 4b still shows heatmaps displaying per-gene SHAP ranks across APOE genotype groups.

6. Although the authors utilize the GWAS/eQTL signal, it might be important to show the integration of genetic evidence more formally by testing colocalization (using tools such as coloc, eCAVIAR) between AD GWAS and brain eQTLs for top predictors to substantiate the "genetically anchored" claim.

We thank the reviewer for this suggestion and agree that a more formal integration of genetic evidence strengthens the interpretation of our results.

In response, we have now performed a formal GWAS–eQTL colocalization analysis between Alzheimer's disease GWAS loci and brain eQTLs for genes prioritized by our predictive modeling framework. Specifically, we applied Bayesian colocalization methods (coloc) using large-scale AD GWAS summary statistics and GTEx brain tissue eQTLs to test whether disease-associated variants and expression quantitative trait loci share a common causal signal.

This analysis revealed multiple loci in which AD GWAS signals colocalize with cis-eQTLs regulating the expression of top predictive genes in a cell-type–specific manner, providing direct genetic support for a subset of TriSCOPE-prioritized features. Importantly, colocalization analyses were restricted to genes identified independently by predictive modeling, ensuring that the reported enrichment reflects genetic support for disease-relevant transcriptional programs rather than genome-wide colocalization rates. While these results do not imply direct causality, they provide strong evidence that inherited AD risk and transcriptional dysregulation converge on shared genetic variants, thereby formally substantiating the "genetically anchored" nature of the identified predictors. We have rewritten the "Genetic colocalization anchors cell-type–specific transcriptional predictors" section of our results to focus on the results of this colocalization analysis, while moving our previous integration of eQTL data to the supplementary results. We have also described our colocalization methods in detail in the methods section titled "GWAS–eQTL Colocalization Analysis". Below are the rewritten results and methods section, respectively:

Modified results section: To more formally link genetic risk to gene regulation, we performed GWAS-eQTL colocalization analyses using brain tissue expression quantitative trait loci [41]. Importantly, colocalization analyses were restricted to genes previously identified as predictive by TriSCOPE, such that the reported proportions reflect the fraction of predictive genes with genetic

support rather than genome-wide colocalization rates. We quantified PP.H4, the posterior probability under a Bayesian colocalization model that a shared causal variant underlies both the GWAS and eQTL association signals at a locus. Across cell types, we identified multiple loci where AD GWAS signals colocalized with cis-eQTLs regulating expression of predictive genes, indicating a shared genetic architecture underlying disease risk and transcriptional dysregulation (Supplemental Fig. 5d). Specifically, strong colocalization was observed for 1.9% of Microglial predictors, 1.1% of Astrocyte predictors, 1.9% of Inhibitory-neuron predictors, 1.0% of Oligodendrocyte predictors, 1.0% of Oligodendrocyte progenitor cell predictors, and 1.9% of Excitatory-neuron predictors (Supplemental Table X). Although the absolute fraction of predictive genes with strong colocalization was modest (≈1–2%), this rate reflects enrichment within a pre-selected, multivariately predictive gene set rather than genome-wide colocalization frequencies, and therefore represents a stringent test of shared genetic architecture.Across 1,107 independent gene-locus pairs, we identified 144 gene-tissue pairs with moderate or stronger evidence of colocalization (PP.H4 ≥ 0.5) and 105 loci with strong evidence of a shared causal variant (PP.H4 $\geq$ 0.8) (Supplementary Table 17). Notably, many of these loci corresponded to genes independently prioritized by our predictive modeling framework, providing genetic support for their relevance to clinical AD. Among the strongest signals, ARL17B demonstrated near-complete colocalization (PP.H4 ≈ 0.99) across multiple brain regions and cell types, indicating that genetic variants influencing ARL17B expression are highly likely to underlie the associated AD GWAS signal. We additionally observed robust colocalization for CLU in excitatory neurons, oligodendrocytes, and oligodendrocyte progenitor cells. Cell-type-specific colocalization was also observed for EGFR in OPCs, USP6NL in microglia, and mitochondrial-associated genes such as NDUFAF6 in oligodendrocytes, suggesting again that distinct genetic regulatory programs contribute to disease risk across cellular compartments. Importantly, these colocalization results do not assert direct causality, but rather provide strong evidence that AD-associated genetic variants and gene expression changes identified by TriSCOPE share a common genetic origin. Together, these findings genetically anchor a subset of TriSCOPE predictors and support a model in which cell-type–specific transcriptional regulation mediates inherited risk for clinical Alzheimer's disease.

Modified methods section: To assess whether cell-type–specific predictive genes were supported by shared genetic regulation between Alzheimer's disease (AD) risk loci and brain gene expression, we performed Bayesian colocalization analysis between AD GWAS summary statistics and brain eQTLs using the coloc framework. We used genome-wide association summary statistics from a large-scale AD GWAS (GCST90027158), aligned to the GRCh38 reference genome. GWAS variants were filtered to autosomal chromosomes (1–22) and harmonized to ensure consistent allele representation. When odds ratios were provided, effect sizes were converted to log-odds ($\beta = \log(OR)$). Variants were deduplicated by genomic position (chr:pos), retaining the most significant association per position to avoid duplicated SNPs. Total sample size and case proportion were specified explicitly (111,326 cases; 677,663 controls), and the GWAS was modeled as a case–control trait within the colocalization framework. Cis-eQTL summary statistics were obtained from GTEx v10 for all available brain tissues. For each tissue, significant variant–gene pairs were loaded from preprocessed eQTL files. Ensembl gene identifiers were harmonized by removing version suffixes, and variant identifiers were parsed to extract chromosome, position, reference allele, and alternate allele. eQTL variants were deduplicated by genomic position (chr:pos), retaining a single representative variant per position. For each tissue, the effective sample size was

approximated using the maximum reported number of samples contributing to eQTL estimation. Colocalization was restricted to genes previously identified as predictive by the supervised learning models. For each major brain cell type (Astrocytes, Microglia, Inhibitory neurons, Excitatory neurons, Oligodendrocytes, and Oligodendrocyte progenitor cells), predictive genes were defined as those with non-zero feature importance in at least two of five donor-aware cross-validation splits. Gene symbols were standardized to uppercase and mapped to Ensembl gene identifiers using BioMart (GRCh38), retaining only genes located on autosomes or sex chromosomes. Genomic coordinates (chromosome, transcription start, and end positions) were used to define gene-centered regions for colocalization testing. For each predictive gene, colocalization was performed independently for every brain tissue. A symmetric genomic window extending ±250 kb from the gene boundaries was defined. Within each region, GWAS and eQTL datasets were subset to variants falling inside the window. Regions were tested only if both datasets contained at least 10 variants and shared at least 10 overlapping SNPs after harmonization by genomic position. Bayesian colocalization was performed using approximate Bayes factors as implemented in the coloc.abf function. GWAS data were modeled as a case–control trait, and eQTL data were modeled as a quantitative trait. For each gene–cell type–tissue combination, posterior probabilities were computed for the five standard hypotheses: no association (H0), GWAS-only association (H1), eQTL-only association (H2), two distinct causal variants (H3), or a shared causal variant (H4). The number of SNPs contributing to each test was recorded. Colocalization results were aggregated across all tested genes, cell types, and brain tissues. To summarize evidence for shared genetic regulation, posterior probability for hypothesis H4 (PP.H4) was used as the primary metric. Results were filtered using predefined thresholds (PP.H4 ≥ 0.5 for moderate evidence and PP.H4 ≥ 0.8 for strong evidence of colocalization). For downstream summaries, genes were considered "anchored" if they (i) were predictive in a given cell type, (ii) were tested in colocalization analysis, and (iii) exhibited PP.H4 ≥ 0.8 in at least one brain tissue. All filtering and aggregation steps were applied using fixed, pre-specified criteria and were not adjusted based on downstream predictive performance, effect size, or prior biological knowledge.

7. The authors might clarify translational relevance by nominating a short, ranked target list with mechanism, tractability, and proposed assays (e.g., LINGO1 inhibition, CRYAB modulation in myelination assays) as part of their results.

We thank the reviewer for the opportunity to clarify the translational relevance of our nominated genes. Across our analysis, we identify 7 genes ARL17B, RASGEF1B, CRYAB, LINGO1, TNFRSF1B, IFI44L, and MT3 as genes that we believe are strong candidates for mechanistic follow-up. We have discussed these in our supplemental discussion section titled "**Proposed Translational Target List and Validation Assays**" and attached it below:

For ARL17B, validation can be performed by CRISPR knockout or overexpression in human iPSC-derived neurons followed by established APP trafficking and processing assays, including ELISA quantification of secreted Aβ and immunoblotting for intracellular APP C-terminal fragments and phospho-tau, directly testing whether altered vesicle trafficking modulates AD-relevant protein handling. For RASGEF1B, neuronal relevance can be evaluated by siRNA or CRISPR perturbation in iPSC-derived excitatory neurons, followed by Ras–ERK pathway activation assays (pERK immunoblotting), dendritic spine density quantification, and synaptic

activity measurements, assays routinely used to assess Ras-dependent neuronal vulnerability in neurodegenerative contexts. For CRYAB, tractable validation consists of gain- or loss-of-function in OPC–neuron co-culture myelination assays, with quantification of myelin basic protein (MBP) expression and internode formation, complemented by cuprizone or AD mouse models to assess white-matter integrity (Ousman et al. 2007). LINGO1 is directly actionable using existing antagonistic antibodies; validation can be performed by LINGO1 blockade in APP/PS1 mice or OPC differentiation assays, measuring remyelination and synaptic preservation (Mi et al. 2005). For MT3 (metallothionein-3), functional validation can be achieved by neuronal overexpression or knockdown followed by oxidative stress and metal-toxicity assays (e.g., hydrogen peroxide or zinc exposure), with readouts including neuronal survival and reactive oxygen species accumulation, leveraging established paradigms demonstrating MT3-dependent modulation of neuronal vulnerability (Hashimoto et al. 2011). For TNFRSF1B (TNFR2), validation can be performed using selective TNFR2 agonists or microglia-specific genetic deletion in AD mouse models, followed by assessment of neuroinflammation, neuronal survival, and amyloid/tau burden, directly testing the neuroprotective TNF signaling axis (Hübschmann et al. 2022). For IFI44L, validation consists of inducing type-I interferon signaling in iPSC-derived microglia (e.g., IFN-β treatment) and suppressing the pathway using IFNAR-blocking antibodies or JAK inhibitors, with readouts including Aβ phagocytosis and inflammatory cytokine production.

To further address the author's question, we performed one additional analysis, which we report both in the Supplemental Results and in Supplemental Table 19. Specifically, we systematically annotated model-identified predictive genes against the full DrugBank database to assess the extent to which high-confidence, cell-type–specific predictors map to existing pharmacologic compounds and are therefore amenable to direct experimental perturbation. We report these results in our supplementary results section titled "DrugBank annotation of predictive genes. We detail the section below:

To further assess the experimental tractability of model-identified genes, we systematically annotated predictive genes against the full DrugBank database. Predictive genes were defined per cell type as those assigned non-zero feature importance in at least two of five independent donor-held-out model splits, yielding 485 unique gene–cell-type pairs. These genes were queried against DrugBank v5 using the complete XML schema, capturing human drug–gene relationships across targets, enzymes, transporters, and carriers. Across cell types, 18–30% of predictive genes had at least one DrugBank annotation, with the highest coverage observed in excitatory neurons (29.6%) and astrocytes (22.9%) (Supplemental Table 19). Among manuscript-emphasized genes, eight showed DrugBank associations, including *EGFR, HSP90AA1, FTH1, APOE, ABCA1, CLU, MAPT,* and *TNFRSF1B*, spanning multiple cell types. Notably, *EGFR* and *HSP90AA1* were linked to extensive pharmacologic repertoires, while *APOE, ABCA1, CLU, MAPT*, and *TNFRSF1B* mapped to smaller but well-defined sets of compounds. These findings indicate that although many predictive genes lack existing drug annotations, a subset of high-confidence genes is directly amenable to pharmacologic perturbation, enabling future targeted experimental validation of model-derived hypotheses.

8. It is also strongly advised to supply a minimal example (subset of cells/genes) demonstrating the entire TriSCOPE workflow including feature selection, AutoML training, grouped CV by donor, SHAP interpretation, DE, pseudotime, so that future users can

adopt without re-engineering. Including inputs/outputs for each stage and expected metrics is essential.

We thank the reviewer for this suggestion. Although we are unable to share the ROSMAP data due to restrictions imposed by the Synapse data use agreement, the TriSCOPE pipeline is fully compatible with publicly available single-cell datasets (e.g., GEO/GSE accessions). To facilitate reproducibility while respecting data use agreements, we have now added a minimal, fully executable TriSCOPE example based on a synthetic microglial dataset. This synthetic dataset preserves the dimensionality, donor structure, and marginal distributional properties of the original data, while containing no real expression values or patient-level information. In our GitHub repository, we provide a compressed archive (triscope_synthetic_demo.zip) that includes a minimal expression matrix and donor-level metadata suitable for direct model training. Separately, we provide within the codebase a complete, runnable Jupyter notebook that uses this dataset to demonstrate feature selection, model training, evaluation, and downstream visualization, including feature importance and ROC curve analyses. Our example dataset is structured to be compatible with the differential expression and pseudotime analysis modules provided in our codebase, enabling users to apply these components without modification if desired. Likewise, outputs generated from these analyses follow the expected format for downstream interpretation modules (e.g., SHAP-based analyses), which are also included in the repository. Our ReadMe now provides the expected output for each major analysis. Together, these resources provide an end-to-end, reproducible example of the TriSCOPE workflow without requiring access to restricted datasets.

9. It would be beneficial to report runtime, peak memory, and hardware for each stage (feature selection, FLAML/LightGBM training, SHAP, DE, pseudotime) as a function of cells × genes for both datasets and include detailed tables in the Supplement.

We now provide a supplemental data file titled Runtime_analysis which reports this information. All modeling components, including Machine Learning, Differential Expression, Pseudotime Analysis, and Colocalization are reported in the Supplemental file. We report in each case data for the largest cohort profiled, along with cells x genes profiled, in that step of the analysis, including runtime and peak memory. We note that we used a Slurm-managed HPC cluster and used single-node jobs for all analysis. Steps such as SHAP, and feature importance analysis were performed downstream of modeling and were performed in interactive notebooks and we therefore provide approximate values.

10. The authors should clearly state typical AUC ranges and variance across splits (CIs), emphasizing that predictive gains are modest at whole-dataset level but stronger at cell type / subcluster resolution; this sets realistic expectations for users deciding when TriSCOPE is beneficial and should be clearly communicated.

We now include an explicit statement in our discussion section: “Applying TriSCOPE across increasing levels of cellular resolution revealed a structured increase in disease-discriminative signal, with modest performance at global levels and the strongest signal emerging within biologically coherent subclusters. This behavior reflects the multivariate nature of Alzheimer’s disease transcriptional programs, which are diluted at coarse resolution but become detectable when conditionally modeled within more homogeneous cellular states”. Secondly, we now include

Supplementary Figure 1f, which provides an overview to guide readers on when application of TriSCOPE is most informative.

11. It is suggested to the authors to provide an explicit negative-control analysis (such as permuted labels, shuffled donor blocks) to bound false-positive discovery rates and report the results at least in Supplement.

We thank the reviewer for this helpful comment. We have now provided an explicit negative-control analysis involving permuted labels, and repeated this across various donor splits (5 seeds following an identical procedure to our previous analysis). We shuffled labels at the level of the donor. Across all cell types we found that the permuted labels negative controls had AUC ranging from 0.46 to 0.49, and underperformed all benchmarked models, including LASSO, GBM, Elastic Net, and Random Forest. We report the results of this analysis in our main results section titled "Cell Type-Specific Biomarker Discovery beyond Demographics and APOE genotype":
Across cell types, all tested architectures achieved relatively concordant performance above chance, while donor-level label permutation controls consistently yielded AUCs below 0.50, confirming that the predictive signal was not driven by data leakage or overfitting. GBM models exceeded permuted controls by a mean ΔAUC of 0.114 ± 0.056 across cell types and achieved the highest performance of the four benchmarked models in two of six populations (Supplementary Table 16).

12. The manuscript notes convergence issues for mixed-effects models and proceeds without a donor random intercept. Given the hierarchical structure of snRNA-seq (many cells nested within donors), the lack of a random effect risks anti-conservative inference (inflated significance from pseudo-replication). Please explicitly discuss this limitation and its expected impact and add robustness checks demonstrating that key DE findings are not driven by within-donor correlation. Concretely, I recommend one or more of the following.

We thank the reviewer for highlighting the risk of pseudo-replication arising from the hierarchical structure of snRNA-seq data and agree that donor-aware modeling is essential for appropriate inference. In our initial analyses, we encountered convergence issues when fitting donor random-effects models, a challenge commonly observed in large, sparse single-cell datasets and exacerbated by the limited number of donors in our original validation cohort (n = 21). In response, we re-implemented donor-aware mixed-effects models using refined preprocessing and normalization consistent with established ROSMAP pipelines, including correction for unwanted technical variation, which stabilizes variance estimation without introducing disease-associated signal. With this refinement and the inclusion of expanded cohorts (n = 48 and n = 427 donors), donor random-intercept models converged for the large majority of genes across all major cell types, and all primary differential expression results reported in the revised manuscript are derived from these donor-aware analyses. To address issues related to pseudo-replication, we now emphasize two key points:

First, we note that all primary differential expression (DE) results reported in the revised manuscript are now derived from donor-aware mixed-effects models that include a donor-specific random intercept. These models explicitly account for the hierarchical structure of the data (cells nested within donors) and are therefore robust to within-donor correlation. The previous fixed-effects DE analyses that omitted a donor random effect—performed initially due to

convergence limitations in our analyses—have been moved entirely to the Supplementary Information and are no longer used to support any primary conclusions.

Secondly, to directly assess whether key findings are driven by within-donor correlation, we performed multiple robustness analyses, now reported in the Supplementary Results: (1) Fixed-effects sensitivity analyses: We fit two alternative fixed-effects DE models that controlled for donor-level covariates without a donor random intercept: (i) age at death, sex, and post-mortem interval (PMI); and (ii) age, sex, APOE genotype, sequencing batch, and PMI. We then compared $\log_2$ fold-change estimates from these models to those obtained from the donor random-effects model. Across all major cell types, effect-size estimates were highly concordant (as we report in our supplemental results), indicating that donor-aware modeling primarily regulates statistical significance rather than the direction or magnitude of transcriptional effects. (2) Predictive modeling safeguards: Although distinct from DE, we further note that all predictive modeling analyses employ strict donor-held-out train/test splits and group-aware cross-validation, ensuring that donor-level correlation cannot inflate predictive performance or downstream gene prioritization. This provides an orthogonal line of evidence that key signals generalize across individuals rather than reflecting within-donor artifacts.

Minor Comments
- The authors should improve figure readability with a single overview panel that maps top predictors per cell type and indicates GWAS/eQTL support.

We have replaced figure 1d, with an explicit figure that shows predictors per cell type that were supported by GWAS/eQTL support following our conditional colocalization analysis, which we now describe in the main results and main methods section.

- It is essential to report uncertainty on all metrics (AUC CIs, feature importance variability across seeds/splits) and include calibration plots for classifiers.

We now explicitly report uncertainty across all evaluation dimensions, as requested. Confidence intervals for ROC AUC are shown directly on all ROC plots in the figure legends and are also provided in the supplemental tables. In addition, we provide comprehensive supplementary results detailing feature importance values and their variability across all five randomized seeds for all predictive models. We have also added probabilistic calibration analyses, including calibration plots and Brier score summaries (Supplemental Table 23), presented in Supplementary Figure 4d and described in a new Supplementary Results section titled “Calibration and Brier Score Analysis.” Together, these additions provide a complete assessment of both discrimination performance and uncertainty across models.

- The authors might want to compare pathway results across methods (GSEA on predictors vs DEGs vs ORA) and report convergent vs unique terms.

We have now added an explicit supplemental results section titled: “**<u>Concordance between machine learning–derived pathway enrichment and differential expression–based pathway analysis”</u>**. Both analyses were performed using an identical pre-ranked GSEA framework, differing

only in the underlying gene sets interrogated—predictive genes derived from machine learning models versus genes identified through differential expression analysis. This design enables a direct comparison of pathway-level signals across complementary statistical perspectives. The supplemental analyses highlight both convergent and method-specific pathways, providing evidence that a subset of biological processes is robustly identified across approaches, while others appear preferentially captured by either predictive or marginal effect–based analyses. We now include figures for both the ML-derived pathway results and the DEG-derived pathway results. This section states the following: To evaluate whether pathways identified by machine-learning–derived feature importance reflected underlying disease biology rather than model-specific artifacts, we compared gene set enrichment results obtained from ML-based GSEA (Supplementary Fig. 1d) with enrichment results derived independently from differentially expressed genes (Supplementary Fig. 1e). Both analyses used an identical pre-ranked GSEA framework and only differ in the underlying gene sets being interrogated. Across cell types, the two approaches showed substantial qualitative concordance in the functional categories identified, despite relying on orthogonal signals—predictive importance across randomized splits versus random effects modeling-based differential expression. In multiple cell types, both analyses converged on RNA-binding and transcription-related functions, as well as metal-ion and zinc-ion binding pathways, suggesting shared regulatory and post-transcriptional processes associated with disease state. Ubiquitin-related activities and protein modification pathways were observed in both ML-derived and DEG-based analyses in neurons, oligodendrocytes, and OPCs, while kinase- and GTPase-related functions appeared consistently in microglia across both frameworks. Importantly, while the relative ranking and statistical strength of individual terms differed—as expected given differences in gene selection criteria and statistical modeling—the recurrence of overlapping functional themes across independent analytical strategies supports the robustness of these biological signals. Together, this concordance indicates that the pathways highlighted by the classifiers largely reflect genuine disease-associated molecular programs captured by differential expression, rather than spurious correlations introduced by model fitting.

- The authors should discuss limitations of cross-sectional prediction (moderate AUCs, lack of temporality) and outline how longitudinal or matched multi-region sampling could resolve them.

We have made our limitations section more explicit. We now provide a discussion section that is as follows:

Our study has several limitations: firstly, our framework is predictive in design, but its primary aim is feature discovery rather than clinical prognostication. The AUCs reflect the use of post-mortem, cross-sectional data, where prediction cannot be the endpoint; instead, the value lies in identification of key genes and cell states that may reflect AD vulnerability independent of known risk factors, and thus warrant further investigation. Importantly, the cross-sectional nature of the data precludes inference about temporal ordering, such that we cannot determine whether the identified transcriptional signatures precede disease onset, track disease progression, or represent late-stage reactive responses to neuropathology. Longitudinal inference, approximated through integration with longitudinal clinical or imaging trajectories or via matched multi-region sampling within the same individuals across brain regions affected at different stages of Alzheimer's disease, would better align molecular variation with disease stage, potentially strengthening signal-to-noise

and improving predictive discrimination. As such, we believe that perturbation experiments will be essential to disentangle whether these transcriptional signatures represent protective adaptations, pathogenic drivers, or reactive epiphenomena. These perturbation experiments, when coupled with our subcluster-level findings, open up avenues for more targeted therapeutic studies. Additionally, we chose to focus on the prefrontal cortex in AD, since it represents a brain region most directly responsible for executive function, where we expected see transcriptional changes associated both with Alzheimer's neuropathology and cognitive decline, however, this focus limits the generalizability of our findings to other brain regions that are affected earlier or differently over the course of disease progression, such as the hippocampus, entorhinal cortex, or subcortical nuclei. Lastly, at present there remains a paucity of high-powered cohort data to examine case-control single cell status of AD [56], meaning future directions include expanding the current resources in sample size to further explore and resolve heterogeneity.

Point-by-Point Response to Reviewers

Reviewer #1

Reviewer #1: *"I thank the authors for meticulously revising the manuscript to consider the reviewers' comments."*

Response: We thank Reviewer #1 for their thorough engagement across both rounds of review, and are grateful for the positive assessment of our revisions. No further changes were requested. Regarding the confidential concern about novelty and scope, we respectfully direct attention to the editorial response above, where we lay out the biological insights that distinguish this work from a methods-only contribution. We emphasize that the manuscript identifies specific, novel, genetically anchored disease genes (e.g., the 27 coloc-PEGs not DEGs), cell-type-specific discordance between clinical and pathological signatures, and convergent disease programs that could not have been obtained by using any of the three modalities we used (differential expression, machine learning prediction, pseudotime analysis)alone.

Reviewer #3

Reviewer #3: *"All the comments were successfully addressed."*

Response: We thank Reviewer #3 for confirming that our prior revisions fully addressed their concerns.

Reviewer #2

We thank Reviewer #2 for their continued engagement and the substantive feedback that has strengthened this manuscript. Below, we address each point in full.

Reviewer #2, Opening Comment

Reviewer #2: *"While the authors acknowledged some aspects of the feedback from the first round of revisions, I do think there are still a couple areas that could be improved to make it very clear what novel insights into potential new avenues of AD biology the triscope method is illuminating as well as clearly disclosing the potential limitations of the approach. That being said, I particularly appreciated the observations about USP6NL and EGFR as genes that were not differentially expressed but looked very promising when considering the GWAS-eQTL colocalization analysis. This sort of observation is what potentially sets the authors' analysis apart from previously reported analyses. Are there other examples like this (or even an updated figure) to show this so it's very clear to the reader what the potentially new and biologically relevant observations actually are?"*

Response:

We thank the reviewer for this comment and for highlighting USP6NL and EGFR as examples of the type of observation that distinguishes TriSCOPE from prior work. We have now substantially expanded the presentation of these "genetically anchored predictors not identified by differential expression" — genes with strong GWAS–eQTL colocalization (PP.H4 ≥ 0.8) and predictive importance in $\geq 2/5$ splits, but no significant differential expression.

In the revised manuscript, we now report the full set of 27 such genes across cell types in the main text Results and provide biological context for the most informative examples. We write in the Results:

> "Across cell types, we identified 27 such genes (Supplementary Table 17). ARL17B was the most prominent, appearing in four cell types (Astrocytes, Microglia, Inhibitory Neurons, and Oligodendrocytes). In Microglia, an additional seven genes met this criterion, including JAZF1, a transcription factor at a genome-wide significant AD locus implicated in lipid metabolism and insulin signaling; SYK, a tyrosine kinase central to microglial phagocytosis and neuroinflammatory signaling; and RIN3, an endocytic adaptor protein at the BIN1/RIN3 AD risk locus. In OPCs, seven genes were identified, and in Oligodendrocytes, five genes including PLEKHA1, linking genetic risk to phosphoinositide signaling. ERC2, involved in neurotransmitter release at active zones, appeared in three cell types (Microglia, OPCs, and Excitatory Neurons), representing a cross-cell-type genetically anchored predictor invisible to differential expression."

Additionally, we now include a colocalization heatmap (Fig. 2d) that displays PP.H4 values for TriSCOPE-prioritized predictive genes across cell types, making the genetically anchored candidates immediately visible to the reader.

Critically, in the new SEA-AD validation analyses, we demonstrate that two of these coloc-PEGs not DEGs — *ARL17B* and *JAZF1* — independently replicate in the SEA-AD cohort. We write in the Results:

> "ARL17B, the most prominent of these in our discovery analyses, was again identified as a predictor in five cell types in SEA-AD (Astrocytes, Microglia, Inhibitory Neurons, Excitatory Neurons, and Oligodendrocytes), confirming that this genetically supported signal is reproducible across cohorts. Additionally, JAZF1 replicated in Microglia as a colocalized predictor not identified by differential expression, providing further evidence that a subset of genetically anchored, predictive genes capture disease-relevant signal that is systematically missed by differential expression."

Reviewer #2, Major Note 1: Non-ROSMAP validation

Reviewer #2: *"I have new concerns regarding their feedback to my first comment from the previous review ('Why use such a small training (n=48) and validation data-set (n=21)? And also why not use some more recently published data as well? Both studies they use are more than five years old….'): First, now they have 3 ROSMAP data-sets, and 1 validation data-set (Lau et al.). I pointed out multiple other data-sets that are not derived from the ROSMAP cohort (e.g. SEA-AD, MSBB, etc…) that are publicly available - why did they only add ROSMAP samples? If the triscope method is truly more robust as the authors claim, shouldn't the results generalize to other large scale cohorts?"*

Response:

We fully agree with the reviewer's concern that validation within ROSMAP alone was insufficient to establish generalizability. In the revised manuscript, we have now performed a comprehensive, independent validation of all three branches of TriSCOPE in the Seattle Alzheimer's Disease Brain Cell Atlas (SEA-AD), an entirely non-ROSMAP cohort comprising 1,395,601 nuclei from the prefrontal cortex of 83 donors (41 controls, 39 dementia, 3 neurotypical), generated by the Allen Institute for Brain Science. No donors overlap with any ROSMAP cohort. This dataset was specifically chosen because the reviewer explicitly named it as a publicly available non-ROSMAP resource.

Branch 1 — Predictive modeling replicates in SEA-AD. We trained cell-type-specific classifiers on SEA-AD using identical modeling procedures. Consistent with ROSMAP, models incorporating gene expression outperformed demographics-only baselines across all six cell types. We write in the Results:

> "In five of six major cell types, classifiers using APOE genotype and gene expression achieved the highest mean AUC: Microglia (0.738 ± 0.031), Inhibitory Neurons (0.709 ± 0.043), Excitatory Neurons (0.670 ± 0.039), Astrocytes (0.657 ± 0.138), and Oligodendrocytes (Genes-only: 0.660 ± 0.052)."

Cross-cohort predictor overlap was highly significant in all six cell types (Fisher's exact tests, background = 19,011 genes):

> "Microglia shared 81 predictive genes (OR = 7.95, $p = 1.86 \times 10^{-39}$), including APOE, ARL17B, BIN1, CTSB, FKBP5, and SPP1. Oligodendrocytes shared 11 predictors (OR = 23.67, $p = 1.22 \times 10^{-10}$), Astrocytes shared 7 (OR = 23.74, $p = 7.17 \times 10^{-8}$), OPCs shared 7 (OR = 6.98, $p = 2.05 \times 10^{-4}$), Excitatory Neurons shared 4 (OR = 39.74, $p = 8.54 \times 10^{-6}$), and Inhibitory Neurons shared 2 (OR = 16.19, $p = 8.13 \times 10^{-3}$)."

Branch 2 — Differential expression replicates in SEA-AD. Donor-aware DE analysis in SEA-AD using the same Poisson GLMM framework identified DEGs that significantly overlapped with ROSMAP-derived DEGs in all six cell types:

> "The strongest enrichment was observed in Oligodendrocytes (72 shared DEGs; OR = 6.33, $p = 7.22 \times 10^{-30}$) and Excitatory Neurons (155 shared DEGs; OR = 2.36, $p = 5.02 \times 10^{-18}$), followed by Microglia (19 shared; OR = 13.47, $p = 1.94 \times 10^{-14}$), Astrocytes (34 shared; OR = 5.55, $p = 1.11 \times 10^{-13}$), OPCs (10 shared; OR = 18.38, $p = 1.17 \times 10^{-9}$), and Inhibitory Neurons (27 shared; OR = 4.29, $p = 3.36 \times 10^{-9}$)."

Branch 3 — Pseudotime trajectories replicate in SEA-AD. We performed pseudotime analysis in SEA-AD and assessed cross-cohort overlap of trajectory-influencing genes:

> "Cross-cohort overlap of trajectory-influencing genes was strongest in Inhibitory Neurons (20 shared genes; OR = 64.60, $p = 2.69 \times 10^{-27}$) and Excitatory Neurons (14 shared genes; OR = 37.49, $p = 7.85 \times 10^{-17}$), with shared genes including DCC, RELN, GRIA4, GRID2, and UNC5D in Inhibitory Neurons, and NRG1, ROBO2, RORA, and CDH13 in Excitatory Neurons."

Genetically anchored predictors not DEGs replicate. As noted above, *ARL17B* replicated as a predictor in five cell types in SEA-AD while remaining absent from DEG lists, and *JAZF1* replicated in Microglia.

Together, these analyses — spanning predictive modeling, differential expression, and pseudotime inference across an entirely independent, non-ROSMAP cohort — directly address the reviewer's concern about generalizability. The full SEA-AD validation is presented as a dedicated main text section ("Independent validation of TriSCOPE") and an expanded Supplementary Results section with per-cell-type statistics for all three branches.

Reviewer #2, Major Note 2: Why only DE on ROSMAP-427 and dataset clarity

Reviewer #2: *"Second, maybe I'm confused as to the analyses they performed, but why did they just do a differential expression analysis in the larger ROSMAP cohort (n=427), and not test and report their cell type specific predictive model generalization performance in this data-set as well? Was this due to computational reasons, or something to do with this vague statement 'We note that retraining models on each dataset is an important but distinct objective, and we view large-scale cross-study model training as a valuable direction for*

future work as harmonized, multi-cohort single-cell resources continue to mature.'? Why couldn't they just test their Mathys 2019 model prediction performance in the Mathys et al 2023 n=427 data-set (without retraining)? Does that mean they did try it and the results did not generalize? If so, they should report this discrepancy and discuss what this means regarding the quality of the relevant data-sets, as readers should know if there are in fact limitations of particular data-sets or potential concerns regarding model generalizability when considering larger data-sets."

Response:

We appreciate this question and agree that predictive modeling on the ROSMAP-427 cohort was an important gap. In the revised manuscript, we have now addressed this directly: we have run TriSCOPE's predictive modeling branch on the full ROSMAP-427 cohort (2.3 million nuclei, 427 donors) and additionally performed comprehensive independent validation of all three TriSCOPE branches (differential expression, machine learning prediction, pseudotime analysis) in SEA-AD.

Predictive modeling on ROSMAP-427. We trained cell-type-specific classifiers on the ROSMAP-427 cohort using the same modeling framework applied to Mathys-48 and SEA-AD. To ensure computational tractability at this scale, we applied per-donor downsampling (see Methods). To assess whether the same molecular programs emerge at 10x the cohort scale, we quantified PEG overlap between Mathys-48-derived and ROSMAP-427-derived predictor sets. Significant overlap was observed in all six cell types (Fisher's exact test, FDR-corrected): Microglia shared 85 predictive genes (fold = 3.43, $p = 2.64 \times 10^{-26}$), Oligodendrocytes shared 62 (fold = 3.02, $p = 2.14 \times 10^{-16}$), OPCs shared 45 (fold = 2.77, $p = 1.00 \times 10^{-10}$), Astrocytes shared 40 (fold = 3.53, $p = 1.19 \times 10^{-12}$), Excitatory Neurons shared 5 (fold = 32.89, $p = 2.46 \times 10^{-7}$), and Inhibitory Neurons shared 2 (fold = 17.96, $p = 5.21 \times 10^{-3}$). Among shared predictors were key genes from our discovery analyses, including *APOE*, *BIN1*, *FKBP5*, *IFI44L*, *HS3ST4*, and *HSPA1A* in Microglia; *ARL17B*, *CRYAB*, and *LINGO1* in Oligodendrocytes; *EGFR* in OPCs; and metallothioneins (*MT1E*, *MT1G*, *MT1M*, *MT3*) in Astrocytes. These results demonstrate that the same cell-type-specific PEGs nominated in our 48-donor discovery cohort are independently recovered when the pipeline is scaled to the full 427-donor ROSMAP release. Full AUC statistics across four model configurations and detailed PEG overlap with gene lists are reported in a new Supplementary Results section, "Predictor Validation in ROSMAP-427".

Independent validation in SEA-AD. In parallel, we performed full TriSCOPE validation in SEA-AD, a completely independent, non-ROSMAP cohort (83 donors, 1.4 million nuclei), as detailed in Major Note 1 above. The highly significant cross-cohort predictor overlap in all six cell types (p-values ranging from 10^{-3} to 10^{-39}) demonstrates that PEG programs generalize across truly independent cohorts.

Complementary roles of ROSMAP-427 and SEA-AD. The two validation datasets address distinct questions. ROSMAP-427 tests within-cohort stability — whether scaling from 48 to 427 donors within the same study recovers the same molecular programs. SEA-AD tests cross-cohort generalizability — whether the same programs emerge in an entirely different institution, brain bank, and donor population. Together, they demonstrate that TriSCOPE-nominated PEGs are both scale-stable and cross-cohort generalizable.

The ROSMAP-427 dataset additionally served as a high-powered resource for donor-aware differential expression. We report in the Results:

> "When evaluated against an independent ROSMAP cohort of 427 donors, only 2–8% of PEGs were differentially expressed, and Spearman correlations between predictor rank and absolute $\log_2$ fold change

were modest (ρ ≈ 0.02–0.21), with similar patterns observed across four independent single-cell AD studies."

This reinforces one of our central findings that PEGs capture multivariate disease-associated structure not reducible to marginal transcriptional effect size — a pattern now confirmed across both DE and predictive modeling in the 427-donor cohort.

On the dataset table: As the reviewer suggested, we have added a main text Table 1 that clearly lists all datasets used, their sample sizes, brain region, and which TriSCOPE analyses were applied to each. We have also ensured that every dataset reference throughout the manuscript is accompanied by a citation and short name (Mathys-48, Lau-21, ROSMAP-427, Murphy-48, ROSMAP-48b, SEA-AD) for unambiguous identification.

On dataset citation clarity: The reviewer noted a specific example on page 37 where the Lau dataset was referenced without explicit citation. We have now ensured that all dataset references throughout the manuscript include the dataset short name and citation, such as "Lau-21 (Lau et al. 2020)" and "SEA-AD (Gabitto et al. 2024)."

Reviewer #2, Major Note 3: ARL17B interpretation and multi-omic evidence

Reviewer #2: *"My second concern is again around the interpretation of the ARL17B result which they still emphasize very strongly in the abstract and later in the manuscript."*

"Again, I would encourage the authors to be cautious with their interpretation of the results of their analyses. While a conditional GWAS-eQTL analysis combined with donor aware cell type differential expression, feature importance in a cell type aware disease diagnosis predictive model, and cell type specific pseudotime is potentially more powerful than a differential expression/eQTL analysis alone for identifying potential mRNAs/genes of interest in disease, any insights are still fundamentally limited by the nature of the instrument variables that you are measuring and analyzing (e.g. mRNA transcript abundance at a fixed point in time in a post mortem tissue across a heterogenous cohort of cases and controls) with regards to a dynamic multi-cellular multi-scale process such as is happening in neurodegeneration. If the authors really want to strengthen their argument, they may consider layering in additional multi-omic evidence that is orthogonal to mRNA expression for some top candidate novel targets (e.g. proteomics or methylation for their short list of high value targets). Furthermore, as noted in their discussion in the MAPT (ARL17B) locus there is a large scale genomic inversion, so I would tread very carefully with the interpretation of strong eQTL within that locus, because there are unusually high levels of linkage disequilibrium throughout the locus that may be resistant even to careful conditional colocalization analysis."

Response:

We fully agree with the reviewer's caution and have made three substantive changes in response. Specifically, we (1) revised the Discussion to explicitly address the MAPT inversion and LD structure at the 17q21.31 locus, (2) added orthogonal multi-omic evidence, including AD-associated methylation changes at KANSL1 and convergent methylation and expression evidence for PICALM, to support genes at this locus, and (3) investigated the reviewer's proteomics suggestion using UKB and ROSMAP plasma proteomic datasets.

1. We have substantially revised the Discussion to explicitly address the MAPT inversion/LD concern. We now write:

"Among pan-cell-type predictors, ARL17B emerged consistently across multiple cell types, aligning with growing evidence that the 17q21.31 MAPT inversion region exerts broad regulatory effects on brain phenotypes. Although ARL17B itself is poorly characterized, it tracks with the inversion haplotype that also modulates KANSL1 and LRRC37A (Pedicone et al. 2024), genes repeatedly implicated in neurodegeneration and AD GWASs. Prior work has similarly linked ARL17B expression in the frontal cortex to genetic risk and cortical thickness, supporting its interpretation as a haplotype-anchored transcriptional marker of disease vulnerability rather than a definitive causal effector. We note that the 17q21.31 locus harbors a large-scale (~1 Mb) genomic inversion that suppresses recombination, producing unusually high linkage disequilibrium among SNPs throughout the region (Pedicone et al. 2024), which may resist even careful conditional colocalization analysis. ARL17B's near-complete colocalization (PP.H4 ≈ 0.99) should therefore be interpreted cautiously, as it may reflect the broad regulatory influence of the inversion haplotype rather than implicating ARL17B as a singular causal effector."

This language directly incorporates the reviewer's point about the inversion and LD structure.

2. We have added methylation evidence for genes at the 17q21 locus. We now write in the Discussion:

"Consistent with multi-omic support for genes at this locus, KANSL1 has been linked to AD-associated DNA methylation changes, and PICALM shows convergent methylation and expression evidence (Ohlei et al. 2023; Eulalio et al. 2024)."

This provides the orthogonal multi-omic evidence the reviewer requested, specifically for the 17q21 locus where it is most relevant. We note that Ohlei et al. (2023) identified KANSL1 as showing highly significant evidence for a putative causal relationship with AD risk through Mendelian randomization integrating peripheral tissue meQTL data with AD GWAS summary statistics, with CpG sites reaching study-wide significance. Critically, their analysis showed that AD- and PD-associated CpGs at 17q21 did not overlap — a dissociation attributed in part to distinct causal variants in high LD driving the associations separately, which reinforces the reviewer's point about interpretive challenges at this locus regardless of molecular modality.

3. Regarding the reviewer's suggestion to layer in proteomics: We investigated this directly. We applied a minimal adaptation of our predictive framework to two independent proteomic datasets: UKB plasma proteomics (Olink Explore panel, ~2,900 proteins; 743 AD cases vs. ~50,169 controls, 80/20 stratified train-test split, LightGBM) and ROSMAP plasma SomaScan (7k aptamer panel; 953 plasma visits [507 NCI, 262 MCI, 167 AD, 17 AD+], one-vs-rest classification per diagnostic class with group-aware train-test splits to prevent donor-level leakage across repeated visits). A substantial fraction of TriSCOPE's nominated genes — including ARL17B, KANSL1, SORL1, and PICALM — are not annotated or measurable in either plasma proteomic panel, reflecting the well-known gap between the plasma proteome and brain-expressed transcripts. For ARL17B specifically, neither MAPT nor any other 17q21 locus protein showed meaningful predictive signal in either dataset. This null result is unsurprising given that plasma proteomics is distal from the brain tissue context of our primary analysis, and is consistent with the reviewer's caution. Where proteomic signal was detectable, we observed convergence for several candidates — for example, PLXDC2 and SPP1, both nominated by TriSCOPE in microglia, showed non-zero predictive importance in ROSMAP plasma proteomics. We have chosen not to include proteomic analyses in the manuscript, as a systematic cross-modal overlap analysis would constitute a distinct study; however, we note these results here to demonstrate that we investigated the reviewer's suggestion thoroughly.

4. We have added proposed experimental validation assays for high-priority targets. In the Supplementary Discussion, we now include a "Proposed Translational Target List and Validation Assays" section providing

specific perturbation experiments for *ARL17B* (CRISPR in iPSC-derived neurons with APP trafficking assays), *RASGEF1B* (siRNA/CRISPR with Ras–ERK pathway assays), *CRYAB* (gain/loss-of-function in OPC–neuron co-culture myelination assays), *LINGO1* (antagonistic antibody blockade in APP/PS1 mice), *MT3* (oxidative stress and metal-toxicity assays), *TNFRSF1B* (selective TNFR2 agonists in AD mouse models), and *IFI44L* (type-I interferon pathway manipulation in iPSC-derived microglia). These proposals directly address the reviewer's broader point about the interpretive limits of transcriptomic association by specifying how causal hypotheses can be tested experimentally.

5. On the general interpretive caution: We agree with the reviewer that all insights from transcriptomic analyses — whether by DE, ML, or any other approach — are fundamentally limited by the nature of the measurements (mRNA abundance at a fixed post-mortem time point). We have substantially expanded the limitations section of the Discussion to address this explicitly. We now write:

> "Importantly, the cross-sectional nature of the data precludes inference about temporal ordering, such that we cannot determine whether the identified transcriptional signatures precede disease onset, track disease progression, or represent late-stage reactive responses to neuropathology. [...] As such, we believe that perturbation experiments will be essential to disentangle whether these transcriptional signatures represent protective adaptations, pathogenic drivers, or reactive epiphenomena."

And:

> "Lastly, our colocalization results do not assert direct causality, but rather provide evidence that AD-associated genetic variants and gene expression changes share a common genetic origin; causal resolution will require perturbational validation."

Reviewer #2, Minor Note 1: Grammar and readability

Reviewer #2: *"The whole manuscript could use a thorough read through to improve grammar and readability (an illustrative example here): pg. 47, Line 1048, 'We found are pan-cell type predictors of ARL17B, LINGO1, RASGEF1B, and SLC26A3 to be predictive across cell types, and found cell type specificity for PLXDC2 in Microglia, GRID2 in Oligodendrocytes, and GFAP in Astrocytes. We additionally found predictive performance to be likewise similar.'"*

Response:

We have performed a thorough readability pass of the entire manuscript. The specific sentence the reviewer flagged has been revised. The Methods section describing the Murphy/Skene QC validation now reads:

> "We found our results to be robust to this higher standard quality-control method, further validating our findings."

The grammatical errors ("are" instead of "our," "likewise similar") and the overly detailed feature-level claims have been replaced with a concise summary that accurately reflects the validation finding without the readability issues the reviewer identified.

In addition, we have reorganized the Supplementary Results for improved logical flow, resolved duplicate section headings, corrected typographical errors, and ensured consistent terminology (e.g., consistent use of "predictively expressed genes (PEGs)" throughout).

Reviewer #2, Minor Note 2: Code availability

Reviewer #2: *"I could not access the code at: https://github.com/AdiVM/TriSCOPE_RNASeq_Alzheimers_Analysis I got a 404 error."*

Response:

We apologize for this issue. All code was included as an attached zip file with our submission, and the repository was set to private at the time of review. The repository is now publicly accessible at https://github.com/AdiVM/TriSCOPE_RNASeq_Alzheimers_Analysis and has been verified to load correctly. The repository includes all predictive modeling, differential expression, pseudotime analysis, GWAS–eQTL colocalization, simulation, and SEA-AD validation code.
