# Supplementary material for: Single-cell machine learning uncovers genetically anchored, cell-type specific programs of Alzheimer’s disease
Source: Nat Commun. 2026 Nov 9;17:11124. doi: 10.1038/s41467-026-77651-5 (PMC13748584; doi:10.1038/s41467-026-77651-5)
Supplement: Supplementary file 4 [file 41467_2026_77651_MOESM2_ESM.pdf]

Description of Additional Supplementary Files

Title: Supplementary Data 1
Description: Feature importance values per cell type across the five experimental splits for a model that used demographics, APOE genotype, and gene expression data to predict clinical AD.

Title: Supplementary Data 2
Description: Feature importance values per cell type across the five experimental splits for a model that used gene expression data to predict clinical AD.

Title: Supplementary Data 3
Description: Feature importance values per cell type across the five experimental splits for a model that used gene expression data to predict AD neuropathology.

Title: Supplementary Data 4
Description: AUC summary on a per cell type level basis, 4 for clinical AD, 4 for AD neuropathology, 2 for the validation dataset.

Title: Supplementary Data 5
Description: Overlap between the GWAS catalog mapped genes and predictively expressed clinical AD genes on a per cell type basis. Enrichment was assessed using two-sided Fisher's exact tests against a background of 17,926 expressed genes, with Benjamini–Hochberg correction applied across cell types.

Title: Supplementary Data 6
Description: Overlap between the GWAS catalog mapped genes and predictively expressed in AD neuropathology genes on a per cell type basis. Enrichment was assessed using two-sided Fisher's exact tests against a background of 17,926 expressed genes, with Benjamini–Hochberg correction applied across cell types.

Title: Supplementary Data 7
Description: Fisher's exact test results quantifying overlap between predictive gene sets (AD vs CERAD) and differential expression gene sets (AD vs control; CERAD-associated), computed per cell type. Tests were one-sided (alternative = greater) against a background of 17,926 expressed genes. Q-values reflect Benjamini–Hochberg correction applied per test type across cell types.

Title: Supplementary Data 8
Description: Differential expression results for clinical AD versus controls across all major brain cell types. Includes $\log_2$ fold changes and FDR-adjusted p-values. Differential expression was

assessed using a donor-aware Poisson generalized linear mixed model with a log link, a library-size offset, RUV covariates as fixed effects, and a random intercept for donor; p-values are from two-sided Wald tests and were adjusted using the Benjamini–Hochberg procedure.

Title: Supplementary Data 9
Description: Differential expression results for AD neuropathology across all major brain cell types. Includes $\log_2$ fold changes and FDR-adjusted p-values. Differential expression was assessed using a donor-aware Poisson generalized linear mixed model with a log link, a library-size offset, RUV covariates as fixed effects, and a random intercept for donor; p-values are from two-sided Wald tests and were adjusted using the Benjamini–Hochberg procedure.

Title: Supplementary Data 10
Description: Overlap of predictive AD genes between the Mathys and Lau datasets, reported on a per-cell-type basis. Genes that emerged as predictive in both datasets are highlighted. Overlap was assessed using one-sided Fisher's exact tests (alternative = greater) against a background of 17,926 expressed genes; p-values are unadjusted.

Title: Supplementary Data 11
Description: Fisher's exact test results for validation analyses, assessing enrichment of overlapping predictive and DE genes between the Mathys and Lau datasets. Tests were one-sided (alternative = greater) against a background of 17,926 expressed genes; p-values are unadjusted.

Title: Supplementary Data 12
Description: Differential expression results for the validation dataset (Mathys et al. 2023), reported per cell type. Includes $\log_2$ fold changes and FDR-adjusted p-values. Differential expression was assessed using a donor-aware Poisson generalized linear mixed model with a log link, a library-size offset, RUV covariates as fixed effects, and a random intercept for donor; p-values are from two-sided Wald tests and were adjusted using the Benjamini–Hochberg procedure.

Title: Supplementary Data 13
Description: Feature importance values per subcluster across the five experimental splits for models trained on gene expression to predict clinical AD. Highlights fine-grained subpopulation-level predictors.

Title: Supplementary Data 14
Description: Summary statistics from pseudotime analyses. The association between pseudotime and disease status was assessed by logistic regression and receiver operating characteristic analysis independently for each random seed; p-values were combined across seeds using Fisher's method and adjusted using the Benjamini–Hochberg procedure.

Title: Supplementary Data 15
Description: Donor-level metadata across donor cohorts analyzed (Mathys-48, Lau-21, ROSMAP-48b, ROSMAP-427, SEA-AD), including sex, APOE genotype, clinical diagnosis and CERAD score. Age at death, years of education, and post-mortem interval are reported as ranges, and participant identifiers have been removed to limit the risk of re-identification.

Title: Supplementary Data 16
Description: Comparison of predictive performance (ROC AUC) across multiple classifier architectures and permuted label baseline.

Title: Supplementary Data 17
Description: Results of GWAS–eQTL colocalization analyses between Alzheimer's disease GWAS loci and brain eQTLs for genes prioritized by predictive modeling, including posterior probabilities for shared causal variants.

Title: Supplementary Data 18
Description: Rank concordance and overlap analyses comparing predictive feature importance rankings, DEG rankings, and differential expression effect sizes across cell types and datasets (internal and external). Rank concordance was assessed using two-sided Spearman rank correlations; overlap between predictor and differentially expressed gene sets was assessed using two-sided Fisher's exact tests. All p-values were adjusted using the Benjamini–Hochberg procedure.

Title: Supplementary Data 19
Description: Drugs annotated in DrugBank that interact with genes identified as predictive by gene expression–based classifiers, stratified by cell type. Drug–gene relationships include targets, enzymes, transporters, and carriers extracted from the full DrugBank XML database.

Title: Supplementary Data 20
Description: Differential expression results comparing clinical AD and control samples under matched levels of neuropathological burden for Microglia and Oligodendrocytes. Differential expression was assessed using a donor-aware Poisson generalized linear mixed model with a log link, a library-size offset, RUV covariates as fixed effects, and a random intercept for donor; p-values are from two-sided Wald tests and were adjusted using the Benjamini–Hochberg procedure.

Title: Supplementary Data 21
Description: Fixed-effects differential expression results controlling for donor-level covariates, provided as a sensitivity analysis to assess robustness of transcriptional effect sizes to model

specification. Differential expression was assessed using a zero-inflated Poisson generalized linear model (glmmTMB) including age at death, sex, post-mortem interval, APOE genotype and sequencing batch as fixed effects; p-values are from two-sided Wald tests and were adjusted using the Benjamini–Hochberg procedure.

Title: Supplementary Data 22
Description: Runtime and peak memory usage for major analytical components of the TriSCOPE pipeline, reported as a function of dataset size (cells × genes) and analysis stage.

Title: Supplementary Data 23
Description: Brier scores summarizing probabilistic calibration of predictive models for clinical AD across cell types.

Title: Supplementary Data 24
Description: Cross-cohort validation results from the Seattle Alzheimer's Disease Brain Cell Atlas (SEA-AD), including per-cell-type Fisher's exact test statistics for overlap of predictive genes, differentially expressed genes, and pseudotime trajectory-influencing genes between ROSMAP-derived and SEA-AD-derived analyses across all six major brain cell types. Additionally, includes PEG results for ROSMAP-427. All Fisher's exact tests were one-sided (alternative = greater) against a background of approximately 19,011 expressed genes, with Benjamini–Hochberg correction applied within each modality.
